# Supplementary material for: OpenMM 7: Rapid development of high performance algorithms for molecular dynamics
Source: PLoS Comput Biol. 2017 Jul 26;13(7):e1005659. doi: 10.1371/journal.pcbi.1005659 (PMC5549999; doi:10.1371/journal.pcbi.1005659)
Supplement: S1 Source Code — (ZIP) [file pcbi.1005659.s002.zip › OpenMM-7.0.1-Source/docs/OpenMMUsersGuide.pdf]

OpenMM Users Guide

Release 7.0

Peter Eastman

May 25, 2016

CONTENTS

1 Introduction **2**
- 1.1 Online Resources . . . 2
- 1.2 Referencing OpenMM . . . 2
- 1.3 Acknowledgments . . . 2

2 The OpenMM Application Layer: Introduction **3**

3 Installing OpenMM **4**
- 3.1 Installing with Conda . . . 4
- 3.2 Installing on Mac OS X . . . 4
- 3.3 Installing on Linux . . . 5
- 3.4 Installing on Windows . . . 6

4 Running Simulations **8**
- 4.1 A First Example . . . 8
- 4.2 Using AMBER Files . . . 10
- 4.3 Using Gromacs Files . . . 11
- 4.4 Using CHARMM Files . . . 12
- 4.5 The Script Builder Application . . . 13
- 4.6 Simulation Parameters . . . 13
  - 4.6.1 Platforms . . . 13
  - 4.6.2 Force Fields . . . 15
  - 4.6.3 AMBER Implicit Solvent . . . 16
  - 4.6.4 Nonbonded Interactions . . . 17
    - Nonbonded Forces for AMOEBA . . . 17
  - 4.6.5 Constraints . . . 18
  - 4.6.6 Heavy Hydrogens . . . 19
  - 4.6.7 Integrators . . . 19
    - Langevin Integrator . . . 19
    - Leapfrog Verlet Integrator . . . 19
    - Brownian Integrator . . . 19
    - Variable Time Step Langevin Integrator . . . 20
    - Variable Time Step Leapfrog Verlet Integrator . . . 20
    - Multiple Time Step Integrator . . . 20
    - aMD Integrator . . . 20
    - Compound Integrator . . . 20
  - 4.6.8 Temperature Coupling . . . 21
  - 4.6.9 Pressure Coupling . . . 21
  - 4.6.10 Energy Minimization . . . 22
  - 4.6.11 Removing Center of Mass Motion . . . 22
  - 4.6.12 Writing Trajectories . . . 22

4.6.13 Recording Other Data . . . 23
4.6.14 Saving Simulation Progress and Results . . . 23

5 Model Building and Editing **25**
5.1 Adding Hydrogens . . . 25
5.2 Adding Solvent . . . 26
5.3 Adding or Removing Extra Particles . . . 27
5.4 Removing Water . . . 27
5.5 Saving The Results . . . 27

6 Advanced Simulation Examples **29**
6.1 Simulated Annealing . . . 29
6.2 Applying an External Force to Particles: a Spherical Container . . . 30
6.3 Extracting and Reporting Forces (and other data) . . . 30
6.4 Computing Energies . . . 31

7 Creating Force Fields **33**
7.1 Basic Concepts . . . 33
7.1.1 Atom Types and Atom Classes . . . 33
7.1.2 Residue Templates . . . 33
7.1.3 Forces . . . 33
7.2 Writing the XML File . . . 34
7.2.1 <AtomTypes> . . . 34
7.2.2 <Residues> . . . 34
7.2.3 Missing residue templates . . . 35
7.2.4 <HarmonicBondForce> . . . 36
7.2.5 <HarmonicAngleForce> . . . 36
7.2.6 <PeriodicTorsionForce> . . . 37
7.2.7 <RBTorsionForce> . . . 37
7.2.8 <CMAPTorsionForce> . . . 38
7.2.9 <NonbondedForce> . . . 39
7.2.10 <GBSAOBCForce> . . . 39
7.2.11 <CustomBondForce> . . . 39
7.2.12 <CustomAngleForce> . . . 40
7.2.13 <CustomTorsionForce> . . . 40
7.2.14 <CustomNonbondedForce> . . . 41
7.2.15 <CustomGBForce> . . . 42
7.2.16 <CustomManyParticleForce> . . . 42
7.2.17 Writing Custom Expressions . . . 43
7.2.18 Tabulated Functions . . . 44
7.2.19 Residue Template Parameters . . . 44
7.3 Using Multiple Files . . . 45
7.4 Extending ForceField . . . 45
7.4.1 Adding new force types . . . 45
7.4.2 Adding residue template generators . . . 46

8 The OpenMM Library: Introduction **48**
8.1 What Is the OpenMM Library? . . . 48
8.1.1 How to get started . . . 48
8.1.2 License . . . 48
8.2 Design Principles . . . 49
8.3 Choice of Language . . . 49
8.4 Architectural Overview . . . 50
8.5 The OpenMM Public API . . . 51
8.6 The OpenMM Low Level API . . . 52

8.7 Platforms 52

9 Compiling OpenMM from Source Code 54
9.1 Prerequisites 54
9.1.1 Get a C++ compiler 54
Mac and Linux: clang or gcc 54
Windows: Visual Studio 54
9.1.2 Install CMake 54
9.1.3 Get the OpenMM source code 55
9.1.4 Other Required Software 55
9.2 Step 1: Configure with CMake 56
9.2.1 Build and source directories 56
9.2.2 Starting CMake 56
Mac and Linux 56
Windows 56
All platforms 56
9.3 Step 2: Generate Build Files with CMake 57
9.3.1 Windows 57
9.3.2 Mac and Linux 57
9.4 Step 3: Build OpenMM 57
9.4.1 Windows 57
9.4.2 Mac and Linux 57
9.5 Step 4: Install OpenMM 58
9.5.1 Windows 58
9.5.2 Mac and Linux 58
9.6 Step 5: Install the Python API 58
9.6.1 Windows 58
9.6.2 Mac and Linux 58
9.7 Step 6: Test your build 58
9.7.1 Windows 58
9.7.2 Mac and Linux 58
9.8 Building the Documentation (Optional) 59
9.8.1 User Guide and Developer Guide 59
9.8.2 Python and C++ API Documentation 59

10 OpenMM Tutorials 61
10.1 Example Files Overview 61
10.2 Running Example Files 61
10.2.1 Visual Studio 62
Determining the platform being used 64
Visualizing the results 64
10.2.2 Mac OS X/Linux 64
Determining the platform being used 65
Visualizing the results 65
Compiling Fortran and C examples 65
10.3 HelloArgon Program 65
10.3.1 Including OpenMM-defined functions 66
10.3.2 Running a program on GPU platforms 66
10.3.3 Running a simulation using the OpenMM public API 66
10.3.4 Error handling for OpenMM 68
10.3.5 Writing out PDB files 68
10.3.6 HelloArgon output 68
10.4 HelloSodiumChloride Program 69
10.4.1 Simple molecular dynamics system 69

10.4.2 Interface routines . . . 70
Units . . . 72
Lennard-Jones potential . . . 72
Opaque handle MyOpenMMData . . . 72
myInitializeOpenMM . . . 73
myGetOpenMMState . . . 75
myStepWithOpenMM . . . 75
myTerminateOpenMM . . . 75
10.5 HelloEthane Program . . . 76

11 Platform-Specific Properties **78**
11.1 OpenCL Platform . . . 78
11.2 CUDA Platform . . . 79
11.3 CPU Platform . . . 79
11.4 Determinism . . . 80

12 Using OpenMM with Software Written in Languages Other than C++ **81**
12.1 C API . . . 81
12.1.1 Mechanics of using the C API . . . 82
12.1.2 Mapping from the C++ API to the C API . . . 82
12.1.3 Exceptions . . . 83
12.1.4 OpenMM_Vec3 helper type . . . 84
12.1.5 Array helper types . . . 84
OpenMM_DoubleArray . . . 85
OpenMM_StringArray . . . 85
OpenMM_Vec3Array . . . 85
OpenMM_BondArray . . . 85
OpenMM_ParameterArray . . . 85
12.2 Fortran 95 API . . . 86
12.2.1 Mechanics of using the Fortran API . . . 86
12.2.2 Mapping from the C++ API to the Fortran API . . . 86
12.2.3 OpenMM_Vec3 helper type . . . 88
12.2.4 Array helper types . . . 88
OpenMM_DoubleArray . . . 89
OpenMM_StringArray . . . 90
OpenMM_Vec3Array . . . 90
OpenMM_BondArray . . . 91
OpenMM_ParameterArray . . . 91
12.3 Python API . . . 92
12.3.1 Installing the Python API . . . 92
Installing on Windows . . . 92
Installing on Linux and Mac . . . 92
12.3.2 Mapping from the C++ API to the Python API . . . 92
12.3.3 Mechanics of using the Python API . . . 93
12.3.4 Units and dimensional analysis . . . 93
Why does the Python API include units? . . . 93
Quantities, units, and dimensions . . . 94
Units examples . . . 94
Arithmetic with units . . . 95
Atomic scale mass and energy units are "per amount" . . . 96
SI prefixes . . . 96
Converting to different units . . . 96
Lists, tuples, vectors, numpy arrays, and Units . . . 96

13 Examples of OpenMM Integration 98
13.1 GROMACS 98
13.2 TINKER-OpenMM 98

14 Testing and Validation of OpenMM 100
14.1 Description of Tests 100
14.1.1 Unit tests 100
14.1.2 System tests 101
14.1.3 Direct comparisons between OpenMM and other programs 101
14.2 Test Results 101
14.2.1 Comparison to Reference Platform 101
14.2.2 Energy Conservation 102
14.2.3 Comparison to Gromacs 103

15 AMOEBA Plugin 104
15.1 OpenMM AMOEBA Supported Forces and Options 104
15.1.1 Supported Forces and Options 104
15.1.2 Supported Integrators 106
15.2 TINKER-OpenMM 106
15.2.1 Building TINKER-OpenMM (Linux) 106
15.2.2 Using TINKER-OpenMM 107
Available outputs 107
Setting the frequency of output data updates 107
Specify the GPU board to use 107
Running comparison tests between TINKER and OpenMM routines 107
Turning off OpenMM / Reverting to TINKER routines 109
15.3 OpenMM AMOEBA Validation 109

16 Ring Polymer Molecular Dynamics (RPMD) Plugin 110

17 Drude Plugin 111

18 The Theory Behind OpenMM: Introduction 112
18.1 Overview 112
18.2 Units 112

19 Standard Forces 114
19.1 HarmonicBondForce 114
19.2 HarmonicAngleForce 114
19.3 PeriodicTorsionForce 114
19.4 RBTorsionForce 115
19.5 CMAPTorsionForce 115
19.6 NonbondedForce 115
19.6.1 Lennard-Jones Interaction 115
19.6.2 Coulomb Interaction Without Cutoff 116
19.6.3 Coulomb Interaction With Cutoff 116
19.6.4 Coulomb Interaction With Ewald Summation 117
19.6.5 Coulomb Interaction With Particle Mesh Ewald 118
19.7 GBSAOBCForce 118
19.7.1 Generalized Born Term 118
19.7.2 Surface Area Term 119
19.8 AndersenThermostat 119
19.9 MonteCarloBarostat 119
19.10 MonteCarloAnisotropicBarostat 120
19.11 MonteCarloMembraneBarostat 120

19.12 CMMotionRemover . . . 121

20 Custom Forces **122**
20.1 CustomBondForce . . . 122
20.2 CustomAngleForce . . . 122
20.3 CustomTorsionForce . . . 123
20.4 CustomNonbondedForce . . . 123
20.5 CustomExternalForce . . . 124
20.6 CustomCompoundBondForce . . . 124
20.7 CustomCentroidBondForce . . . 125
20.8 CustomManyParticleForce . . . 125
20.9 CustomGBForce . . . 125
20.10 CustomHbondForce . . . 127
20.11 Writing Custom Expressions . . . 127

21 Integrators **128**
21.1 VerletIntegrator . . . 128
21.2 LangevinIntegrator . . . 128
21.3 BrownianIntegrator . . . 128
21.4 VariableVerletIntegrator . . . 129
21.5 VariableLangevinIntegrator . . . 129
21.6 CustomIntegrator . . . 130

22 Other Features **131**
22.1 Periodic Boundary Conditions . . . 131
22.2 LocalEnergyMinimizer . . . 131
22.3 XMLSerializer . . . 132
22.4 Force Groups . . . 132
22.5 Virtual Sites . . . 132
22.6 Random Numbers with Stochastic Integrators and Forces . . . 133

Bibliography **135**

Portions copyright (c) 2008-2016 Stanford University and the Authors

Contributors: Kyle Beauchamp, Christopher Bruns, John Chodera, Peter Eastman, Mark Friedrichs, Joy P. Ku, Tom Markland, Vijay Pande, Randy Radmer, Michael Sherman, Jason Swails, Lee-Ping Wang

Permission is hereby granted, free of charge, to any person obtaining a copy of this document (the "Document"), to deal in the Document without restriction, including without limitation the rights to use, copy, modify, merge, publish, distribute, sublicense, and/or sell copies of the Document, and to permit persons to whom the Document is furnished to do so, subject to the following conditions:

This copyright and permission notice shall be included in all copies or substantial portions of the Document.

THE DOCUMENT IS PROVIDED "AS IS", WITHOUT WARRANTY OF ANY KIND, EXPRESS OR IMPLIED, INCLUDING BUT NOT LIMITED TO THE WARRANTIES OF MERCHANTABILITY, FITNESS FOR A PARTICULAR PURPOSE AND NONINFRINGEMENT. IN NO EVENT SHALL THE AUTHORS, CONTRIBUTORS OR COPYRIGHT HOLDERS BE LIABLE FOR ANY CLAIM, DAMAGES OR OTHER LIABILITY, WHETHER IN AN ACTION OF CONTRACT, TORT OR OTHERWISE, ARISING FROM, OUT OF OR IN CONNECTION WITH THE DOCUMENT OR THE USE OR OTHER DEALINGS IN THE DOCUMENT.

CHAPTER

ONE

INTRODUCTION

OpenMM consists of two parts:

1. A set of libraries that lets programmers easily add molecular simulation features to their programs
2. An "application layer" that exposes those features to end users who just want to run simulations

This guide is divided into three sections:

- Part I (Chapters *2*-*7*) describes the application layer. It is relevant to all users, but especially relevant to people who want to use OpenMM as a stand-alone application for running simulations.
- Part II (Chapters *8*-*17*) describes how to use the OpenMM libraries within your own applications. It is primarily relevant to programmers who want to write simulation applications.
- Part III (Chapters *18*-*22*) describes the mathematical theory behind the features found in OpenMM. It is relevant to all users.

1.1 Online Resources

You can find more documentation and other material at our website http://simtk.org/home/openmm. Among other things there is a discussion forum, a wiki, and videos of lectures on using OpenMM.

1.2 Referencing OpenMM

Any work that uses OpenMM should cite the following publication:

P. Eastman, M. S. Friedrichs, J. D. Chodera, R. J. Radmer, C. M. Bruns, J. P. Ku, K. A. Beauchamp, T. J. Lane, L.-P. Wang, D. Shukla, T. Tye, M. Houston, T. Stich, C. Klein, M. R. Shirts, and V. S. Pande. "OpenMM 4: A Reusable, Extensible, Hardware Independent Library for High Performance Molecular Simulation." J. Chem. Theor. Comput. 9(1): 461-469. (2013).

We depend on academic research grants to fund the OpenMM development efforts; citations of our publication will help demonstrate the value of OpenMM.

1.3 Acknowledgments

OpenMM software and all related activities, such as this manual, are funded by the Simbios National Center for Biomedical Computing through the National Institutes of Health Roadmap for Medical Research, Grant U54 GM072970. Information on the National Centers can be found at http://nihroadmap.nih.gov/bioinformatics.

CHAPTER TWO

THE OPENMM APPLICATION LAYER: INTRODUCTION

The first thing to understand about the OpenMM "application layer" is that it is not exactly an application in the traditional sense: there is no program called "OpenMM" that you run. Rather, it is a collection of libraries written in the Python programming language. Those libraries can easily be chained together to create Python programs that run simulations. But don't worry! You don't need to know anything about Python programming (or programming at all) to use it. Nearly all molecular simulation applications ask you to write some sort of "script" that specifies the details of the simulation to run. With OpenMM, that script happens to be written in Python. But it is no harder to write than those for most other applications, and this guide will teach you everything you need to know. There is even a graphical interface that can write the script for you based on a simple set of options (see Section *4.5*), so you never need to type a single line of code!

On the other hand, if you don't mind doing a little programming, this approach gives you enormous power and flexibility. Your script has complete access to the entire OpenMM application programming interface (API), as well as the full power of the Python language and libraries. You have complete control over every detail of the simulation, from defining the molecular system to analyzing the results.

CHAPTER

THREE

INSTALLING OPENMM

Follow these instructions to install OpenMM. There also is an online troubleshooting guide that describes common problems and how to fix them (http://wiki.simtk.org/openmm/FAQApp).

There are two ways to install OpenMM: using the Conda package manager (http://conda.pydata.org), or with standalone installers. (A third option is to compile it from source, which is discussed in Chapter 9.) Using Conda is the easier method, and is recommended for most users. It is described first, and then the following sections describe how to use the standalone installers for various platforms.

3.1 Installing with Conda

Conda is included as part of the Anaconda Python distribution, which you can download from http://docs.continuum.io/anaconda/install. This is a Python distribution specifically designed for scientific applications, with many of the most popular mathematical and scientific packages preinstalled. Alternatively you can use Miniconda (available from http://conda.pydata.org/miniconda.html), which includes only Python itself, plus the Conda package manager. That offers a much smaller initial download, with the ability to then install only the packages you want.

1. Begin by installing the most recent 64 bit, Python 3.x version of either Anaconda or Miniconda.

   2. (Optional) If you want to run OpenMM on a GPU, install CUDA and/or OpenCL.

   - If you have an Nvidia GPU, download CUDA 7.5 from https://developer.nvidia.com/cuda-downloads. Be sure to install both the drivers and toolkit. OpenCL is included with the CUDA drivers.
   - If you have an AMD GPU and are using Linux or Windows, download the latest version of the Catalyst driver from http://support.amd.com. On OS X, OpenCL is included with the operating system and is supported on OS X 10.10.3 or later.

3. Open a command line terminal and type the following command

```
conda install -c omnia openmm
```

4. Verify your installation by typing the following command:

```
python -m simtk.testInstallation
```

This command confirms that OpenMM is installed, checks whether GPU acceleration is available (via the OpenCL and/or CUDA platforms), and verifies that all platforms produce consistent results.

3.2 Installing on Mac OS X

OpenMM works on Mac OS X 10.7 or later. OpenCL is supported on OS X 10.10.3 or later.

1. Download the pre-compiled binary of OpenMM for Mac OS X from https://simtk.org/project/xml/downloads.xml?group_id=161, then double click the .zip file to expand it.

2. If you have not already done so, install Apple's Xcode developer tools from the App Store. They are required to use OpenMM. (With Xcode 4.3 and later, you must then launch Xcode, open the Preferences window, go to the Downloads tab, and tell it to install the command line tools. With Xcode 4.2 and earlier, the command line tools are automatically installed when you install Xcode.)

3. (Optional) If you have an Nvidia GPU and want to use the CUDA platform, download CUDA 7.5 from https://developer.nvidia.com/cuda-downloads. Be sure to install both the drivers and toolkit.

4. (Optional) If you plan to use the CPU platform, it is recommended that you install FFTW, available from http://www.fftw.org. When configuring it, be sure to specify single precision and multiple threads (the `--enable-float` and `--enable-threads` options). OpenMM will still work without FFTW, but the performance of particle mesh Ewald (PME) will be much worse.

5. Launch the Terminal application. Change to the OpenMM directory by typing

```
cd <openmm_directory>
```

where `<openmm_directory>` is the path to the OpenMM folder. Then run the install script by typing

```
sudo ./install.sh
```

It will prompt you for an install location and the path to the python executable. Unless you are certain you know what you are doing, accept the defaults for both options.

6. (Optional) To use the CUDA platform on an Nvidia GPU, you must add the CUDA libraries to your library path so your computer knows where to find them. You can do this by typing

```
export DYLD_LIBRARY_PATH=/usr/local/cuda/lib
```

This will affect only the particular Terminal window you type it into. If you want to run OpenMM in another Terminal window, you must type the above command in the new window.

7. Verify your installation by typing the following command:

```
python -m simtk.testInstallation
```

This command confirms that OpenMM is installed, checks whether GPU acceleration is available (via the OpenCL and/or CUDA platforms), and verifies that all platforms produce consistent results.

Important Note: Some Mac laptops have two GPUs, only one of which is capable of running OpenMM. If you have a laptop, open the System Preferences and go to the Energy Saver panel. There will be a checkbox labeled "Automatic graphics switching", which should be disabled. Otherwise, trying to run OpenMM may produce an error. You will only see this option if your laptop has two GPUs

3.3 Installing on Linux

1. Download the pre-compiled binary of OpenMM for Linux from https://simtk.org/project/xml/downloads.xml?group_id=161, then double click the .zip file to expand it.

2. Make sure you have Python 2.6 or higher (earlier versions will not work) and a C++ compiler (typically **gcc** or **clang**) installed on your computer. You can check what version of Python is installed by typing `python --version` into a console window.

3. (Optional) If you want to run OpenMM on a GPU, install CUDA and/or OpenCL.

- If you have an Nvidia GPU, download CUDA 7.5 from https://developer.nvidia.com/cuda-downloads. Be sure to install both the drivers and toolkit. OpenCL is included with the CUDA drivers.

- If you have an AMD GPU, download the latest version of the Catalyst driver from http://support.amd.com.

4. (Optional) If you plan to use the CPU platform, it is recommended that you install FFTW. It is probably available through your system's package manager such as **yum** or **apt-get**. Alternatively, you can download it from http://www.fftw.org. When configuring it, be sure to specify single precision and multiple threads (the `--enable-float` and `--enable-threads` options). OpenMM will still work without FFTW, but the performance of particle mesh Ewald (PME) will be much worse.

5. In a console window, change to the OpenMM directory by typing

```
cd <openmm_directory>
```

where `<openmm_directory>` is the path to the OpenMM folder. Then run the install script by typing

```
sudo ./install.sh
```

It will prompt you for an install location and the path to the python executable. Unless you are certain you know what you are doing, accept the defaults for both options.

6. (Optional) To use the CUDA platform on an Nvidia GPU, you must add the CUDA libraries to your library path so your computer knows where to find them. You can do this by typing

```
export LD_LIBRARY_PATH=/usr/local/cuda/lib
```

This will affect only the particular console window you type it into. If you want to run OpenMM in another console window, you must type the above command in the new window.

7. Verify your installation by typing the following command:

```
python -m simtk.testInstallation
```

This command confirms that OpenMM is installed, checks whether GPU acceleration is available (via the OpenCL and/or CUDA platforms), and verifies that all platforms produce consistent results.

3.4 Installing on Windows

1. Download the pre-compiled binary of OpenMM for Windows from https://simtk.org/project/xml/downloads.xml?group_id=161, then double click the .zip file to expand it. Move the files to `C:\Program Files\OpenMM`.

2. Make sure you have the 64-bit version of Python 3.3 or 3.4 (other versions will not work) installed on your computer. To do this, launch the Python program (either the command line version or the GUI version). The first line in the Python window will indicate the version you have, as well as whether you have a 32-bit or 64-bit version.

3. Double click the Python API Installer to install the Python components. (On some versions of Windows, a "Program Compatibility Assistant" window may appear with the warning, "This program might not have installed correctly." This is just Microsoft trying to scare you. Click "This program installed correctly" and ignore it.)

4. (Optional) If you want to run OpenMM on a GPU, install CUDA and/or OpenCL.

- If you have an Nvidia GPU, download CUDA 7.5 from https://developer.nvidia.com/cuda-downloads. Be sure to install both the drivers and toolkit. OpenCL is included with the CUDA drivers.
- If you have an AMD GPU, download the latest version of the Catalyst driver from http://support.amd.com.

5. (Optional) If you plan to use the CPU platform, it is recommended that you install FFTW. Precompiled binaries are available from http://www.fftw.org. OpenMM will still work without FFTW, but the performance of particle mesh Ewald (PME) will be much worse.

6. Before running OpenMM, you must add the OpenMM and FFTW libraries to your PATH environment variable. You may also need to add the Python executable to your PATH.

- To find out if the Python executable is already in your PATH, open a command prompt window by clicking on *Start* → *Programs* → *Accessories* → *Command Prompt*. (On Windows 7, select *Start* → *All Programs* → *Accessories* → *Command Prompt*). Type

```
python
```

  If you get an error message, such as "'python' is not recognized as an internal or external command, operable program or batch file," then you need to add Python to your PATH. To do so, locate it by typing

```
dir C:\py*
```

  The files are typically located in a directory like `C:\Python33`. Remember this location. You will need to enter it, along with the location of the OpenMM libraries, later in this process.

- Click on *Start* → *Control Panel* → *System* (On Windows 7, select *Start* → *Control Panel* → *System and Security* → *System*)
- Click on the *Advanced* tab or the *Advanced system settings* link
- Click *Environment Variables*
- Under *System variables*, select the line for *Path* and click *Edit...*
- Add `C:\Program Files\OpenMM\lib` and `C:\Program Files\OpenMM\lib\plugins` to the "Variable value". If you also need to add Python or FFTW to your PATH, enter their directory locations here. Directory locations need to be separated by semi-colons (;).

  If you installed OpenMM somewhere other than the default location, you must also set `OPENMM_PLUGIN_DIR` to point to the plugins directory. If this variable is not set, it will assume plugins are in the default location (`C:\Program Files\OpenMM\lib\plugins`).

7. Verify your installation by typing the following command:

```
python -m simtk.testInstallation
```

This command confirms that OpenMM is installed, checks whether GPU acceleration is available (via the OpenCL and/or CUDA platforms), and verifies that all platforms produce consistent results.

CHAPTER

FOUR

RUNNING SIMULATIONS

4.1 A First Example

Let's begin with our first example of an OpenMM script. It loads a PDB file called `input.pdb` that defines a biomolecular system, parameterizes it using the Amber99SB force field and TIP3P water model, energy minimizes it, simulates it for 10,000 steps with a Langevin integrator, and saves a snapshot frame to a PDB file called `output.pdb` every 1000 time steps.

```
from simtk.openmm.app import *
from simtk.openmm import *
from simtk.unit import *
from sys import stdout

pdb = PDBFile('input.pdb')
forcefield = ForceField('amber99sb.xml', 'tip3p.xml')
system = forcefield.createSystem(pdb.topology, nonbondedMethod=PME,
        nonbondedCutoff=1*nanometer, constraints=HBonds)
integrator = LangevinIntegrator(300*kelvin, 1/picosecond, 0.002*picoseconds)
simulation = Simulation(pdb.topology, system, integrator)
simulation.context.setPositions(pdb.positions)
simulation.minimizeEnergy()
simulation.reporters.append(PDBReporter('output.pdb', 1000))
simulation.reporters.append(StateDataReporter(stdout, 1000, step=True,
        potentialEnergy=True, temperature=True))
simulation.step(10000)
```

Example 4-1

You can find this script in the `examples` folder of your OpenMM installation. It is called `simulatePdb.py`. To execute it from a command line, go to your terminal/console/command prompt window (see Section *3* on setting up the window to use OpenMM). Navigate to the `examples` folder by typing

```
cd <examples_directory>
```

where the typical directory is `/usr/local/openmm/examples` on Linux and Mac machines and `C:\Program Files\OpenMM\examples` on Windows machines.

Then type

```
python simulatePdb.py
```

You can name your own scripts whatever you want. Let's go through the script line by line and see how it works.

```
from simtk.openmm.app import *
from simtk.openmm import *
from simtk.unit import *
from sys import stdout
```

These lines are just telling the Python interpreter about some libraries we will be using. Don't worry about exactly what they mean. Just include them at the start of your scripts.

```
pdb = PDBFile('input.pdb')
```

This line loads the PDB file from disk. (The `input.pdb` file in the `examples` directory contains the villin headpiece in explicit solvent.) More precisely, it creates a `PDBFile` object, passes the file name `input.pdb` to it as an argument, and assigns the object to a variable called `pdb`. The `PDBFile` object contains the information that was read from the file: the molecular topology and atom positions. Your file need not be called `input.pdb`. Feel free to change this line to specify any file you want, though it must contain all of the atoms needed by the force field. (More information on how to add missing atoms and residues using OpenMM tools can be found in Chapter *5*.) Make sure you include the single quotes around the file name. OpenMM also can load files in the newer PDBx/mmCIF format: just change `PDBFile` to `PDBxFile`.

```
forcefield = ForceField('amber99sb.xml', 'tip3p.xml')
```

This line specifies the force field to use for the simulation. Force fields are defined by XML files. OpenMM includes XML files defining lots of standard force fields (see Section *4.6.2*). If you find you need to extend the repertoire of force fields available, you can find more information on how to create these XML files in Chapter *7*. In this case we load two of those files: `amber99sb.xml`, which contains the Amber99SB force field, and `tip3p.xml`, which contains the TIP3P water model. The `ForceField` object is assigned to a variable called `forcefield`.

```
system = forcefield.createSystem(pdb.topology, nonbondedMethod=PME,
        nonbondedCutoff=1*nanometer, constraints=HBonds)
```

This line combines the force field with the molecular topology loaded from the PDB file to create a complete mathematical description of the system we want to simulate. (More precisely, we invoke the `ForceField` object's `createSystem()` function. It creates a `System` object, which we assign to the variable `system`.) It specifies some additional options about how to do that: use particle mesh Ewald for the long range electrostatic interactions (`nonbondedMethod=PME`), use a 1 nm cutoff for the direct space interactions (`nonbondedCutoff=1*nanometer`), and constrain the length of all bonds that involve a hydrogen atom (`constraints=HBonds`). Note the way we specified the cutoff distance 1 nm using `1*nanometer`: This is an example of the powerful units tracking and automatic conversion facility built into the OpenMM Python API that makes specifying unit-bearing quantities convenient and less error-prone. We could have equivalently specified `10*angstrom` instead of `1*nanometer` and achieved the same result. The units system will be described in more detail later, in Section *12.3.4*.

```
integrator = LangevinIntegrator(300*kelvin, 1/picosecond, 0.002*picoseconds)
```

This line creates the integrator to use for advancing the equations of motion. It specifies a `LangevinIntegrator`, which performs Langevin dynamics, and assigns it to a variable called `integrator`. It also specifies the values of three parameters that are specific to Langevin dynamics: the simulation temperature (300 K), the friction coefficient (1 ps^{-1}), and the step size (0.002 ps).

```
simulation = Simulation(pdb.topology, system, integrator)
```

This line combines the molecular topology, system, and integrator to begin a new simulation. It creates a `Simulation` object and assigns it to a variable called `simulation`. A `Simulation` object manages all the processes involved in running a simulation, such as advancing time and writing output.

```
simulation.context.setPositions(pdb.positions)
```

This line specifies the initial atom positions for the simulation: in this case, the positions that were loaded from the PDB file.

```
simulation.minimizeEnergy()
```

This line tells OpenMM to perform a local energy minimization. It is usually a good idea to do this at the start of a simulation, since the coordinates in the PDB file might produce very large forces.

```
simulation.reporters.append(PDBReporter('output.pdb', 1000))
```

This line creates a "reporter" to generate output during the simulation, and adds it to the `Simulation` object's list of reporters. A `PDBReporter` writes structures to a PDB file. We specify that the output file should be called `output.pdb`, and that a structure should be written every 1000 time steps.

```
simulation.reporters.append(StateDataReporter(stdout, 1000, step=True,
        potentialEnergy=True, temperature=True))
```

It can be useful to get regular status reports as a simulation runs so you can monitor its progress. This line adds another reporter to print out some basic information every 1000 time steps: the current step index, the potential energy of the system, and the temperature. We specify `stdout` (not in quotes) as the output file, which means to write the results to the console. We also could have given a file name (in quotes), just as we did for the `PDBReporter`, to write the information to a file.

```
simulation.step(10000)
```

Finally, we run the simulation, integrating the equations of motion for 10,000 time steps. Once it is finished, you can load the PDB file into any program you want for analysis and visualization (VMD, PyMol, AmberTools, etc.).

4.2 Using AMBER Files

OpenMM can build a system in several different ways. One option, as shown above, is to start with a PDB file and then select a force field with which to model it. Alternatively, you can use AmberTools to model your system. In that case, you provide a `prmtop` file and an `inpcrd` file. OpenMM loads the files and creates a `System` from them. This is illustrated in the following script. It can be found in OpenMM's `examples` folder with the name `simulateAmber.py`.

```
from simtk.openmm.app import *
from simtk.openmm import *
from simtk.unit import *
from sys import stdout

prmtop = AmberPrmtopFile('input.prmtop')
inpcrd = AmberInpcrdFile('input.inpcrd')
system = prmtop.createSystem(nonbondedMethod=PME, nonbondedCutoff=1*nanometer,
        constraints=HBonds)
integrator = LangevinIntegrator(300*kelvin, 1/picosecond, 0.002*picoseconds)
simulation = Simulation(prmtop.topology, system, integrator)
simulation.context.setPositions(inpcrd.positions)
if inpcrd.boxVectors is not None:
    simulation.context.setPeriodicBoxVectors(*inpcrd.boxVectors)
simulation.minimizeEnergy()
simulation.reporters.append(PDBReporter('output.pdb', 1000))
simulation.reporters.append(StateDataReporter(stdout, 1000, step=True,
        potentialEnergy=True, temperature=True))
simulation.step(10000)
```

Example 4-2

This script is very similar to the previous one. There are just a few significant differences:

```
prmtop = AmberPrmtopFile('input.prmtop')
inpcrd = AmberInpcrdFile('input.inpcrd')
```

In these lines, we load the prmtop file and inpcrd file. More precisely, we create `AmberPrmtopFile` and `AmberInpcrdFile` objects and assign them to the variables `prmtop` and `inpcrd`, respectively. As before, you can change these lines to specify any files you want. Be sure to include the single quotes around the file names.

Note: The `AmberPrmtopFile` reader provided by OpenMM only supports "new-style" `prmtop` files introduced in AMBER 7. The AMBER distribution still contains a number of example files that are in the "old-style" `prmtop` format. These "old-style" files will not run in OpenMM.

Next, the `System` object is created in a different way:

```
system = prmtop.createSystem(nonbondedMethod=PME, nonbondedCutoff=1*nanometer,
        constraints=HBonds)
```

In the previous section, we loaded the topology from a PDB file and then had the force field create a system based on it. In this case, we don't need a force field; the `prmtop` file already contains the force field parameters, so it can create the system directly.

```
simulation = Simulation(prmtop.topology, system, integrator)
simulation.context.setPositions(inpcrd.positions)
```

Notice that we now get the topology from the `prmtop` file and the atom positions from the `inpcrd` file. In the previous section, both of these came from a PDB file, but AMBER puts the topology and positions in separate files. We also add the following lines:

```
if inpcrd.boxVectors is not None:
    simulation.context.setPeriodicBoxVectors(*inpcrd.boxVectors)
```

For periodic systems, the `prmtop` file specifies the periodic box vectors, just as a PDB file does. When we call `createSystem()`, it sets those as the default periodic box vectors, to be used automatically for all simulations. However, the `inpcrd` may *also* specify periodic box vectors, and if so we want to use those ones instead. For example, if the system has been equilibrated with a barostat, the box vectors may have changed during equilibration. We therefore check to see if the `inpcrd` file contained box vectors. If so, we call `setPeriodicBoxVectors()` to tell it to use those ones, overriding the default ones provided by the `System`.

4.3 Using Gromacs Files

A third option for creating your system is to use the Gromacs setup tools. They produce a `gro` file containing the coordinates and a `top` file containing the topology. OpenMM can load these exactly as it did the AMBER files. This is shown in the following script. It can be found in OpenMM's `examples` folder with the name `simulateGromacs.py`.

```
from simtk.openmm.app import *
from simtk.openmm import *
from simtk.unit import *
from sys import stdout

gro = GromacsGroFile('input.gro')
top = GromacsTopFile('input.top', periodicBoxVectors=gro.getPeriodicBoxVectors(),
        includeDir='/usr/local/gromacs/share/gromacs/top')
system = top.createSystem(nonbondedMethod=PME, nonbondedCutoff=1*nanometer,
        constraints=HBonds)
integrator = LangevinIntegrator(300*kelvin, 1/picosecond, 0.002*picoseconds)
simulation = Simulation(top.topology, system, integrator)
simulation.context.setPositions(gro.positions)
simulation.minimizeEnergy()
simulation.reporters.append(PDBReporter('output.pdb', 1000))
simulation.reporters.append(StateDataReporter(stdout, 1000, step=True,
        potentialEnergy=True, temperature=True))
simulation.step(10000)
```

Example 4-3

This script is nearly identical to the previous one, just replacing `AmberInpcrdFile` and `AmberPrmtopFile` with `GromacsGroFile` and `GromacsTopFile`. Note that when we create the `GromacsTopFile`, we specify values for two extra options. First, we specify `periodicBoxVectors=gro.getPeriodicBoxVectors()`. Unlike OpenMM and AMBER, which can store periodic unit cell information with the topology, Gromacs only stores it with the coordinates. To let `GromacsTopFile` create a `Topology` object, we therefore need to tell it the periodic box vectors that were loaded from the `gro` file. You only need to do this if you are simulating a periodic system. For implicit solvent simulations, it usually can be omitted.

Second, we specify `includeDir='/usr/local/gromacs/share/gromacs/top'`. Unlike AMBER, which stores all the force field parameters directly in a `prmtop` file, Gromacs just stores references to force field definition files that are installed with the Gromacs application. OpenMM needs to know where to find these files, so the `includeDir` parameter specifies the directory containing them. If you omit this parameter, OpenMM will assume the default location `/usr/local/gromacs/share/gromacs/top`, which is often where they are installed on Unix-like operating systems. So in Example 4-3 we actually could have omitted this parameter, but if the Gromacs files were installed in any other location, we would need to include it.

4.4 Using CHARMM Files

Yet another option is to load files created by the CHARMM setup tools, or other compatible tools such as VMD. Those include a `psf` file containing topology information, and an ordinary PDB file for the atomic coordinates. (Coordinates can also be loaded from CHARMM coordinate or restart files using the `CharmmCrdFile` and `CharmmRstFile` classes). In addition, you must provide a set of files containing the force field definition to use. This can involve several different files with varying formats and filename extensions such as `par`, `prm`, `top`, `rtf`, `inp`, and `str`. To do this, load all the definition files into a `CharmmParameterSet` object, then include that object as the first parameter when you call `createSystem()` on the `CharmmPsfFile`.

```
from simtk.openmm.app import *
from simtk.openmm import *
from simtk.unit import *
from sys import stdout, exit, stderr

psf = CharmmPsfFile('input.psf')
pdb = PDBFile('input.pdb')
params = CharmmParameterSet('charmm22.rtf', 'charmm22.prm')
system = psf.createSystem(params, nonbondedMethod=NoCutoff,
        nonbondedCutoff=1*nanometer, constraints=HBonds)
integrator = LangevinIntegrator(300*kelvin, 1/picosecond, 0.002*picoseconds)
simulation = Simulation(psf.topology, system, integrator)
simulation.context.setPositions(pdb.positions)
simulation.minimizeEnergy()
simulation.reporters.append(PDBReporter('output.pdb', 1000))
simulation.reporters.append(StateDataReporter(stdout, 1000, step=True,
        potentialEnergy=True, temperature=True))
simulation.step(10000)
```

Example 4-4

Note that both the CHARMM and XPLOR versions of the psf file format are supported.

4.5 The Script Builder Application

One option for writing your own scripts is to start with one of the examples given above (the one in Section *4.1* if you are starting from a PDB file, section *4.2* if you are starting from AMBER prmtop and inpcrd files, section *4.3* if you are starting from Gromacs gro and top files, or section *4.4* if you are starting from CHARMM files), then customize it to suit your needs. Another option is to use the OpenMM Script Builder application.

This is a web application available at https://builder.openmm.org. It provides a graphical interface with simple choices for all the most common simulation options, then automatically generates a script based on them. As you change the settings, the script is instantly updated to reflect them. Once everything is set the way you want, click the *Save Script* button to save it to disk, or simply copy and paste it into a text editor.

4.6 Simulation Parameters

Now let's consider lots of ways you might want to customize your script.

4.6.1 Platforms

When creating a Simulation, you can optionally tell it what Platform to use. OpenMM includes four platforms: Reference, CPU, CUDA, and OpenCL. For a description of the differences between them, see Section *8.7*. There are three ways in which the Platform can be chosen:

1. By default, OpenMM will try to select the fastest available Platform. Usually its choice will be reasonable, but sometimes you may want to change it.

2. Alternatively, you can set the OPENMM_DEFAULT_PLATFORM environment variable to the name of the Platform to use. This overrides the default logic.

3. Finally, you can explicitly specify a Platform object in your script when you create the Simulation. The following lines specify to use the CUDA platform:

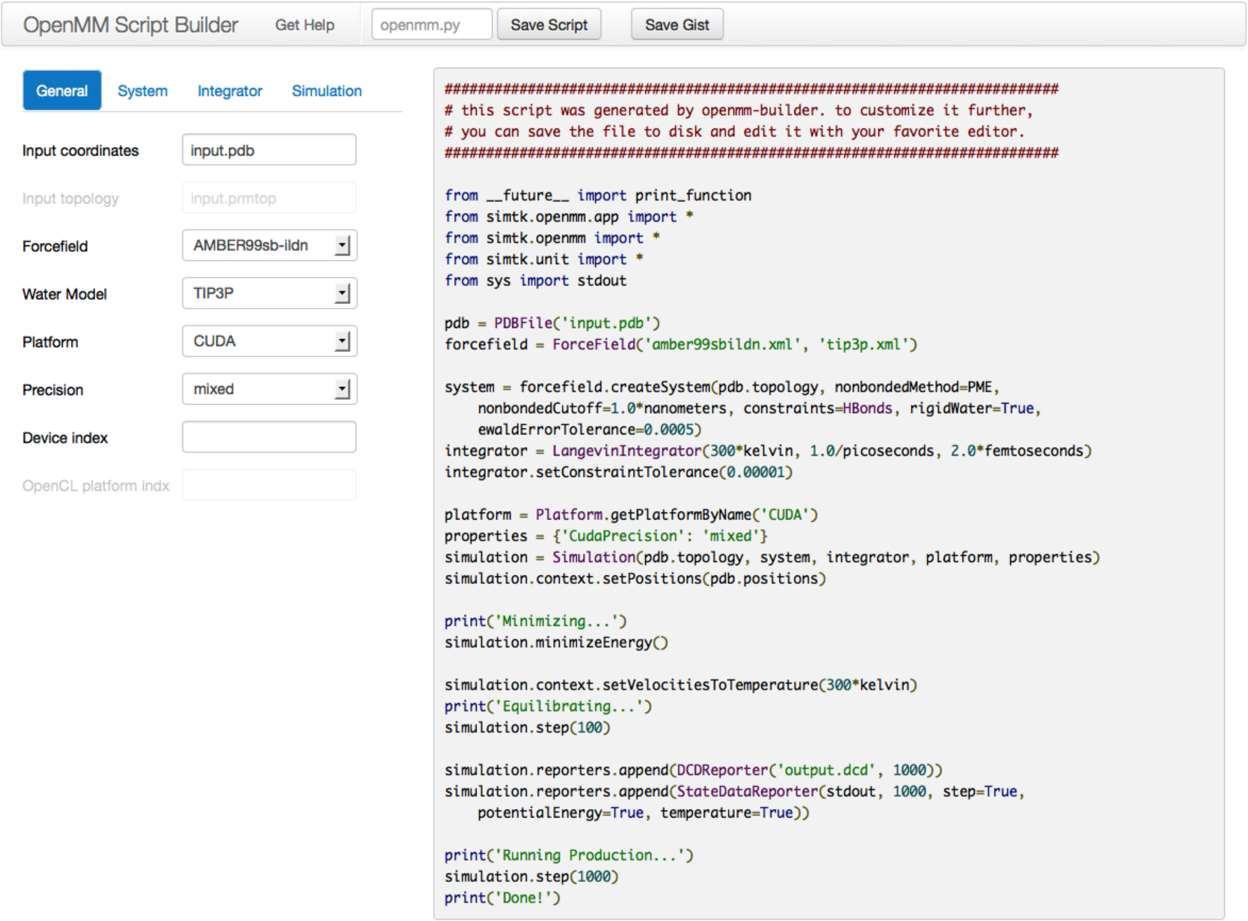

Figure 4-1: The Script Builder application

```
platform = Platform.getPlatformByName('CUDA')
simulation = Simulation(prmtop.topology, system, integrator, platform)
```

The platform name should be one of `OpenCL`, `CUDA`, `CPU`, or `Reference`.

You also can specify platform-specific properties that customize how calculations should be done. See Chapter *11* for details of the properties that each Platform supports. For example, the following lines specify to parallelize work across two different GPUs (CUDA devices 0 and 1), doing all computations in double precision:

```
platform = Platform.getPlatformByName('CUDA')
properties = {'CudaDeviceIndex': '0,1', 'CudaPrecision': 'double'}
simulation = Simulation(prmtop.topology, system, integrator, platform, properties)
```

4.6.2 Force Fields

When you create a force field, you specify one or more XML files from which to load the force field definition. Most often, there will be one file to define the main force field, and possibly a second file to define the water model (either implicit or explicit). For example:

```
forcefield = ForceField('amber99sb.xml', 'tip3p.xml')
```

For the main force field, OpenMM provides the following options:

File	Force Field
`amber96.xml`	Amber96*[1]*
`amber99sb.xml`	Amber99*[2]* with modified backbone torsions*[3]*
`amber99sbildn.xml`	Amber99SB plus improved side chain torsions*[4]*
`amber99sbnmr.xml`	Amber99SB with modifications to fit NMR data*[5]*
`amber03.xml`	Amber03*[6]*
`amber10.xml`	Amber10 (documented in the AmberTools manual as *ff10*)
`amoeba2009.xml`	AMOEBA 2009*[7]*. This force field is deprecated. It is recommended to use AMOEBA 2013 instead.
`amoeba2013.xml`	AMOEBA 2013*[8]*
`charmm_polar_2013.xml`	CHARMM 2013 polarizable force field*[9]*

The AMBER files do not include parameters for water molecules. This allows you to separately select which water model you want to use. For simulations that include explicit water molecules, you should also specify one of the following files:

File	Water Model
`tip3p.xml`	TIP3P water model*[10]*
`tip3pfb.xml`	TIP3P-FB water model*[11]*
`tip4pew.xml`	TIP4P-Ew water model*[12]*
`tip4pfb.xml`	TIP4P-FB water model*[11]*
`tip5p.xml`	TIP5P water model*[13]*
`spce.xml`	SPC/E water model*[14]*
`swm4ndp.xml`	SWM4-NDP water model*[15]*

For the polarizable force fields (AMOEBA and CHARMM), only one explicit water model is currently available and the water parameters are included in the same file as the macromolecule parameters. Also, the polarizable force fields only include parameters for amino acids and ions, not for nucleic acids.

If you want to include an implicit solvation model, you can also specify one of the following files:

File	Implicit Solvation Model
amber96_obc.xml	GBSA-OBC solvation model[16] for use with Amber96 force field
amber99_obc.xml	GBSA-OBC solvation model for use with Amber99 force fields
amber03_obc.xml	GBSA-OBC solvation model for use with Amber03 force field
amber10_obc.xml	GBSA-OBC solvation model for use with Amber10 force field
amoeba2009_gk.xml	Generalized Kirkwood solvation model[17] for use with AMOEBA 2009 force field
amoeba2013_gk.xml	Generalized Kirkwood solvation model for use with AMOEBA 2013 force field

For example, to use the GBSA-OBC solvation model with the Amber99SB force field, you would type:

```
forcefield = ForceField('amber99sb.xml', 'amber99_obc.xml')
```

Note that the GBSA-OBC parameters in these files are those used in TINKER.[18] They are designed for use with Amber force fields, but they are different from the parameters found in the AMBER application.

If you are running a vacuum simulation, you do not need to specify a water model. The following line specifies the Amber10 force field and no water model. If you try to use it with a PDB file that contains explicit water, it will produce an error since no water parameters are defined:

```
forcefield = ForceField('amber10.xml')
```

Be aware that some force fields and water models include "extra particles", such as lone pairs or Drude particles. Examples include the CHARMM polarizable force field and all of the 4 and 5 site water models. To use these force fields, you must first add the extra particles to the `Topology`. See section 5.3 for details.

4.6.3 AMBER Implicit Solvent

When creating a system from a prmtop file you do not specify force field files, so you need a different way to tell it to use implicit solvent. This is done with the `implicitSolvent` parameter:

```
system = prmtop.createSystem(implicitSolvent=OBC2)
```

OpenMM supports all of the Generalized Born models used by AMBER. Here are the allowed values for `implicitSolvent`:

Value	Meaning
None	No implicit solvent is used.
HCT	Hawkins-Cramer-Truhlar GBSA model[19] (corresponds to igb=1 in AMBER)
OBC1	Onufriev-Bashford-Case GBSA model[16] using the GB^{OBC}I parameters (corresponds to igb=2 in AMBER).
OBC2	Onufriev-Bashford-Case GBSA model[16] using the GB^{OBC}II parameters (corresponds to igb=5 in AMBER). This is the same model used by the GBSA-OBC files described in Section 4.6.2.
GBn	GBn solvation model[20] (corresponds to igb=7 in AMBER).
GBn2	GBn2 solvation model[21] (corresponds to igb=8 in AMBER).

You can further control the solvation model in a few ways. First, you can specify the dielectric constants to use for the solute and solvent:

```
system = prmtop.createSystem(implicitSolvent=OBC2, soluteDielectric=1.0,
        solventDielectric=80.0)
```

If they are not specified, the solute and solvent dielectrics default to 1.0 and 78.5, respectively. These values were chosen for consistency with AMBER, and are slightly different from those used elsewhere in OpenMM: when building a system from a force field, the solvent dielectric defaults to 78.3.

You also can model the effect of a non-zero salt concentration by specifying the Debye-Huckel screening parameter[22]:

```
system = prmtop.createSystem(implicitSolvent=OBC2, implicitSolventKappa=1.0/nanometer)
```

4.6.4 Nonbonded Interactions

When creating the system (either from a force field or a prmtop file), you can specify options about how nonbonded interactions should be treated:

```
system = prmtop.createSystem(nonbondedMethod=PME, nonbondedCutoff=1*nanometer)
```

The `nonbondedMethod` parameter can have any of the following values:

Value	Meaning
`NoCutoff`	No cutoff is applied.
`CutoffNonPeriodic`	The reaction field method is used to eliminate all interactions beyond a cutoff distance. Not valid for AMOEBA.
`CutoffPeriodic`	The reaction field method is used to eliminate all interactions beyond a cutoff distance. Periodic boundary conditions are applied, so each atom interacts only with the nearest periodic copy of every other atom. Not valid for AMOEBA.
`Ewald`	Periodic boundary conditions are applied. Ewald summation is used to compute long range interactions. (This option is rarely used, since PME is much faster for all but the smallest systems.) Not valid for AMOEBA.
`PME`	Periodic boundary conditions are applied. The Particle Mesh Ewald method is used to compute long range interactions.

When using any method other than `NoCutoff`, you should also specify a cutoff distance. Be sure to specify units, as shown in the examples above. For example, `nonbondedCutoff=1.5*nanometers` or `nonbondedCutoff=12*angstroms` are legal values.

When using `Ewald` or `PME`, you can optionally specify an error tolerance for the force computation. For example:

```
system = prmtop.createSystem(nonbondedMethod=PME, nonbondedCutoff=1*nanometer,
        ewaldErrorTolerance=0.00001)
```

The error tolerance is roughly equal to the fractional error in the forces due to truncating the Ewald summation. If you do not specify it, a default value of 0.0005 is used.

Nonbonded Forces for AMOEBA

For the AMOEBA force field, the valid values for the `nonbondedMethod` are `NoCutoff` and `PME`. The other nonbonded methods, `CutoffNonPeriodic`, `CutoffPeriodic`, and `Ewald` are unavailable for this force field.

For implicit solvent runs using AMOEBA, only the `nonbondedMethod` option `NoCutoff` is available.

Lennard-Jones Interaction Cutoff Value

In addition, for the AMOEBA force field a cutoff for the Lennard-Jones interaction independent of the value used for the electrostatic interactions may be specified using the keyword `vdwCutoff`.

```
system = forcefield.createSystem(nonbondedMethod=PME, nonbondedCutoff=1*nanometer,
        ewaldErrorTolerance=0.00001, vdwCutoff=1.2*nanometer)
```

If `vdwCutoff` is not specified, then the value of `nonbondedCutoff` is used for the Lennard-Jones interactions.

Specifying the Polarization Method

When using the AMOEBA force field, OpenMM allows the induced dipoles to be calculated in any of three different ways. The slowest but potentially most accurate method is to iterate the calculation until the dipoles converge to a specified tolerance. To select this, specify `polarization='mutual'`. Use the `mutualInducedTargetEpsilon` option to select the tolerance; for most situations, a value of 0.00001 works well. Alternatively you can specify `polarization='extrapolated'`. This uses an analytic approximation *[23]* to estimate what the fully converged dipoles will be without actually continuing the calculation to convergence. In many cases this can be significantly faster with only a small loss in accuracy. Finally, you can specify `polarization='direct'` to use the direct polarization approximation, in which induced dipoles depend only on the fixed multipoles, not on other induced dipoles. This is even faster, but it produces very different forces from mutual polarization, so it should only be used with force fields that have been specifically parameterized for use with this approximation.

Here are examples of using each method:

```
system = forcefield.createSystem(nonbondedMethod=PME, nonbondedCutoff=1*nanometer,
    vdwCutoff=1.2*nanometer, polarization='mutual', mutualInducedTargetEpsilon=0.00001)

system = forcefield.createSystem(nonbondedMethod=PME, nonbondedCutoff=1*nanometer,
    vdwCutoff=1.2*nanometer, polarization='extrapolated')

system = forcefield.createSystem(nonbondedMethod=PME, nonbondedCutoff=1*nanometer,
    vdwCutoff=1.2*nanometer, polarization='direct')
```

Implicit Solvent and Solute Dielectrics

For implicit solvent simulations using the AMOEBA force field, the `amoeba2013_gk.xml` file should be included in the initialization of the force field:

```
forcefield = ForceField('amoeba2009.xml', 'amoeba2009_gk.xml')
```

Only the `nonbondedMethod` option `NoCutoff` is available for implicit solvent runs using AMOEBA. In addition, the solvent and solute dielectric values can be specified for implicit solvent simulations:

```
system=forcefield.createSystem(nonbondedMethod=NoCutoff, soluteDielectric=2.0,
        solventDielectric=80.0)
```

The default values are 1.0 for the solute dielectric and 78.3 for the solvent dielectric.

4.6.5 Constraints

When creating the system (either from a force field or an AMBER `prmtop` file), you can optionally tell OpenMM to constrain certain bond lengths and angles. For example,

```
system = prmtop.createSystem(nonbondedMethod=NoCutoff, constraints=HBonds)
```

The `constraints` parameter can have any of the following values:

Value	Meaning
`None`	No constraints are applied. This is the default value.
`HBonds`	The lengths of all bonds that involve a hydrogen atom are constrained.
`AllBonds`	The lengths of all bonds are constrained.
`HAngles`	The lengths of all bonds are constrained. In addition, all angles of the form H-X-H or H-O-X (where X is an arbitrary atom) are constrained.

The main reason to use constraints is that it allows one to use a larger integration time step. With no constraints, one is typically limited to a time step of about 1 fs for typical biomolecular force fields like AMBER or CHARMM. With `HBonds` constraints, this can be increased to about 2 fs. With `HAngles`, it can be further increased to 3.5 or 4 fs.

Regardless of the value of this parameter, OpenMM makes water molecules completely rigid, constraining both their bond lengths and angles. You can disable this behavior with the `rigidWater` parameter:

```
system = prmtop.createSystem(nonbondedMethod=NoCutoff, constraints=None, rigidWater=False)
```

Be aware that flexible water may require you to further reduce the integration step size, typically to about 0.5 fs.

Note: The AMOEBA forcefield is intended to be used without constraints.

4.6.6 Heavy Hydrogens

When creating the system (either from a force field or an AMBER `prmtop` file), you can optionally tell OpenMM to increase the mass of hydrogen atoms. For example,

```
system = prmtop.createSystem(hydrogenMass=4*amu)
```

This applies only to hydrogens that are bonded to heavy atoms, and any mass added to the hydrogen is subtracted from the heavy atom. This keeps their total mass constant while slowing down the fast motions of hydrogens. When combined with constraints (typically `constraints=AllBonds`), this allows a further increase in integration step size.

4.6.7 Integrators

OpenMM offers a choice of several different integration methods. You select which one to use by creating an integrator object of the appropriate type.

Langevin Integrator

In the examples of the previous sections, we used Langevin integration:

```
integrator = LangevinIntegrator(300*kelvin, 1/picosecond, 0.002*picoseconds)
```

The three parameter values in this line are the simulation temperature (300 K), the friction coefficient (1 ps^{-1}), and the step size (0.002 ps). You are free to change these to whatever values you want. Be sure to specify units on all values. For example, the step size could be written either as `0.002*picoseconds` or `2*femtoseconds`. They are exactly equivalent.

Leapfrog Verlet Integrator

A leapfrog Verlet integrator can be used for running constant energy dynamics. The command for this is:

```
integrator = VerletIntegrator(0.002*picoseconds)
```

The only option is the step size.

Brownian Integrator

Brownian (diffusive) dynamics can be used by specifying the following:

```
integrator = BrownianIntegrator(300*kelvin, 1/picosecond, 0.002*picoseconds)
```

The parameters are the same as for Langevin dynamics: temperature (300 K), friction coefficient (1 ps^{-1}), and step size (0.002 ps).

Variable Time Step Langevin Integrator

A variable time step Langevin integrator continuously adjusts its step size to keep the integration error below a specified tolerance. In some cases, this can allow you to use a larger average step size than would be possible with a fixed step size integrator. It also is very useful in cases where you do not know in advance what step size will be stable, such as when first equilibrating a system. You create this integrator with the following command:

```
integrator = VariableLangevinIntegrator(300*kelvin, 1/picosecond, 0.001)
```

In place of a step size, you specify an integration error tolerance (0.001 in this example). It is best not to think of this value as having any absolute meaning. Just think of it as an adjustable parameter that affects the step size and integration accuracy. Smaller values will produce a smaller average step size. You should try different values to find the largest one that produces a trajectory sufficiently accurate for your purposes.

Variable Time Step Leapfrog Verlet Integrator

A variable time step leapfrog Verlet integrator works similarly to the variable time step Langevin integrator in that it continuously adjusts its step size to keep the integration error below a specified tolerance. The command for this integrator is:

```
integrator = VariableVerletIntegrator(0.001)
```

The parameter is the integration error tolerance (0.001), whose meaning is the same as for the Langevin integrator.

Multiple Time Step Integrator

The `MTSIntegrator` class implements the rRESPA multiple time step algorithm*[24]*. This allows some forces in the system to be evaluated more frequently than others. For details on how to use it, consult the API documentation.

aMD Integrator

There are three different integrator types that implement variations of the aMD*[25]* accelerated sampling algorithm: `AMDIntegrator`, `AMDForceGroupIntegrator`, and `DualAMDIntegrator`. They perform integration on a modified potential energy surface to allow much faster sampling of conformations. For details on how to use them, consult the API documentation.

Compound Integrator

The `CompoundIntegrator` class is useful for cases where you want to use multiple integration algorithms within a single simulation. It allows you to create multiple integrators, then switch back and forth between them. For details on how to use it, consult the API documentation.

4.6.8 Temperature Coupling

If you want to run a simulation at constant temperature, using a Langevin integrator (as shown in the examples above) is usually the best way to do it. OpenMM does provide an alternative, however: you can use a Verlet integrator, then add an Andersen thermostat to your system to provide temperature coupling.

To do this, we can add an `AndersenThermostat` object to the `System` as shown below.

```
...
system = prmtop.createSystem(nonbondedMethod=PME, nonbondedCutoff=1*nanometer,
        constraints=HBonds)
system.addForce(AndersenThermostat(300*kelvin, 1/picosecond))
integrator = VerletIntegrator(0.002*picoseconds)
...
```

The two parameters of the Andersen thermostat are the temperature (300 K) and collision frequency (1 ps^{-1}).

4.6.9 Pressure Coupling

All the examples so far have been constant volume simulations. If you want to run at constant pressure instead, add a Monte Carlo barostat to your system. You do this exactly the same way you added the Andersen thermostat in the previous section:

```
...
system = prmtop.createSystem(nonbondedMethod=PME, nonbondedCutoff=1*nanometer,
        constraints=HBonds)
system.addForce(MonteCarloBarostat(1*bar, 300*kelvin))
integrator = LangevinIntegrator(300*kelvin, 1/picosecond, 0.002*picoseconds)
...
```

The parameters of the Monte Carlo barostat are the pressure (1 bar) and temperature (300 K). The barostat assumes the simulation is being run at constant temperature, but it does not itself do anything to regulate the temperature.

Warning: It is therefore critical that you always use it along with a Langevin integrator or Andersen thermostat, and that you specify the same temperature for both the barostat and the integrator or thermostat. Otherwise, you will get incorrect results.

There also is an anisotropic barostat that scales each axis of the periodic box independently, allowing it to change shape. When using the anisotropic barostat, you can specify a different pressure for each axis. The following line applies a pressure of 1 bar along the X and Y axes, but a pressure of 2 bar along the Z axis:

```
system.addForce(MonteCarloAnisotropicBarostat((1, 1, 2)*bar, 300*kelvin))
```

Another feature of the anisotropic barostat is that it can be applied to only certain axes of the periodic box, keeping the size of the other axes fixed. This is done by passing three additional parameters that specify whether the barostat should be applied to each axis. The following line specifies that the X and Z axes of the periodic box should not be scaled, so only the Y axis can change size.

```
system.addForce(MonteCarloAnisotropicBarostat((1, 1, 1)*bar, 300*kelvin,
        False, True, False))
```

There is a third barostat designed specifically for simulations of membranes. It assumes the membrane lies in the XY plane, and treats the X and Y axes of the box differently from the Z axis. It also applies a uniform surface tension in the plane of the membrane. The following line adds a membrane barostat that applies a pressure of 1 bar and a surface tension of 200 bar*nm. It specifies that the X and Y axes are treated isotropically while the Z axis is free to change independently.

```
system.addForce(MonteCarloMembraneBarostat(1*bar, 200*bar*nanometer,
    MonteCarloMembraneBarostat.XYIsotropic, MonteCarloMembraneBarostat.ZFree, 300*kelvin))
```

See the API documentation for details about the allowed parameter values and their meanings.

4.6.10 Energy Minimization

As seen in the examples, performing a local energy minimization takes a single line in the script:

```
simulation.minimizeEnergy()
```

In most cases, that is all you need. There are two optional parameters you can specify if you want further control over the minimization. First, you can specify a tolerance for when the energy should be considered to have converged:

```
simulation.minimizeEnergy(tolerance=5*kilojoule/mole)
```

If you do not specify this parameter, a default tolerance of 10 kJ/mole is used.

Second, you can specify a maximum number of iterations:

```
simulation.minimizeEnergy(maxIterations=100)
```

The minimizer will exit once the specified number of iterations is reached, even if the energy has not yet converged. If you do not specify this parameter, the minimizer will continue until convergence is reached, no matter how many iterations it takes.

These options are independent. You can specify both if you want:

```
simulation.minimizeEnergy(tolerance=0.1*kilojoule/mole, maxIterations=500)
```

4.6.11 Removing Center of Mass Motion

By default, `System` objects created with the OpenMM application tools add a `CMMotionRemover` that removes all center of mass motion at every time step so the system as a whole does not drift with time. This is almost always what you want. In rare situations, you may want to allow the system to drift with time. You can do this by specifying the `removeCMMotion` parameter when you create the System:

```
system = forcefield.createSystem(pdb.topology, nonbondedMethod=NoCutoff,
        removeCMMotion=False)
```

4.6.12 Writing Trajectories

OpenMM can save simulation trajectories to disk in three formats: PDB, PDBx/mmCIF, and DCD. All of these are widely supported formats, so you should be able to read them into most analysis and visualization programs.

To save a trajectory, just add a "reporter" to the simulation, as shown in the example scripts above:

```
simulation.reporters.append(PDBReporter('output.pdb', 1000))
```

The two parameters of the `PDBReporter` are the output filename and how often (in number of time steps) output structures should be written. To use PDBx/mmCIF or DCD format, just replace `PDBReporter` with `PDBxReporter` or `DCDReporter`. The parameters represent the same values:

```
simulation.reporters.append(DCDReporter('output.dcd', 1000))
```

4.6.13 Recording Other Data

In addition to saving a trajectory, you may want to record other information over the course of a simulation, such as the potential energy or temperature. OpenMM provides a reporter for this purpose also. Create a `StateDataReporter` and add it to the simulation:

```
simulation.reporters.append(StateDataReporter('data.csv', 1000, time=True,
        kineticEnergy=True, potentialEnergy=True))
```

The first two parameters are the output filename and how often (in number of time steps) values should be written. The remaining arguments specify what values should be written at each report. The available options are `step` (the index of the current time step), `time`, `progress` (what percentage of the simulation has completed), `remainingTime` (an estimate of how long it will take the simulation to complete), `potentialEnergy`, `kineticEnergy`, `totalEnergy`, `temperature`, `volume` (the volume of the periodic box), `density` (the total system mass divided by the volume of the periodic box), and `speed` (an estimate of how quickly the simulation is running). If you include either the `progress` or `remainingTime` option, you must also include the `totalSteps` parameter to specify the total number of time steps that will be included in the simulation. One line is written to the file for each report containing the requested values. By default the values are written in comma-separated-value (CSV) format. You can use the `separator` parameter to choose a different separator. For example, the following line will cause values to be separated by spaces instead of commas:

```
simulation.reporters.append(StateDataReporter('data.txt', 1000, progress=True,
        temperature=True, totalSteps=10000, separator=' '))
```

4.6.14 Saving Simulation Progress and Results

There are three built-in ways to save the results of your simulation in OpenMM (additional methods can be written yourself or imported through other packages like mdtraj or parmed). If you are simply interested in saving the structure, you can write it out as a PDB file using `PDBFile.writeFile()`. You can see an example of this in the modeller section *5.5*.

If you are hoping to save more information than just positions, you can use `simulation.saveState()`. This will save the entire state of the simulation, including positions, velocities, box dimensions and much more in an XML file. This same file can be loaded back into OpenMM and used to continue the simulation. Importantly, because this file is a text file, it can be transfered between different platforms and different versions of OpenMM. A potential downside to this approach is that state files are often quite large, and may not fit all use cases. Here's an example of how to use it:

```
simulation.saveState('output.xml')
```

To load the simulation back in:

```
simulation.loadState('output.xml')
```

There is a third way to save your simulation, known as a checkpoint file, which will save the entire simulation as a binary file. It will allow you to exactly continue a simulation if the need arises (though whether the simulation is deterministic depends on platform and methods, see *11.4*). There are important caveats to this approach, however. This binary can only be used to restart simulations on machines with the same hardware and the same OpenMM version as the one that saved it. Therefore, it should only be used when it's clear that won't be an issue.

```
simulation.saveCheckpoint('state.chk')
```

And can be loaded back in like this:

```
simulation.loadCheckpoint('state.chk')
```

Finally, OpenMM comes with a built-in reporter for saving checkpoints, the `CheckpointReporter`, which can be helpful in restarting simulations that failed unexpectedly or due to outside reasons (e.g. server crash). To save a checkpoint file every 5,000 steps, for example:

```
simulation.reporters.append(CheckpointReporter('checkpnt.chk', 5000))
```

Note that the checkpoint reporter will overwrite the last checkpoint file.

CHAPTER

FIVE

MODEL BUILDING AND EDITING

Sometimes you have a PDB file that needs some work before you can simulate it. Maybe it doesn't contain hydrogen atoms (which is common for structures determined by X-ray crystallography), so you need to add them. Or perhaps you want to simulate the system in explicit water, but the PDB file doesn't contain water molecules. Or maybe it does contain water molecules, but they contain the wrong number of interaction sites for the water model you want to use. OpenMM's Modeller class can fix problems such as these.

To use it, create a `Modeller` object, providing the initial `Topology` and atom positions. You then can invoke various modelling functions on it. Each one modifies the system in some way, creating a new `Topology` and list of positions. When you are all done, you can retrieve them from the `Modeller` and use them as the starting point for your simulation:

```
...
pdb = PDBFile('input.pdb')
modeller = Modeller(pdb.topology, pdb.positions)
# ... Call some modelling functions here ...
system = forcefield.createSystem(modeller.topology, nonbondedMethod=PME)
simulation = Simulation(modeller.topology, system, integrator)
simulation.context.setPositions(modeller.positions)
```

Example 5-1

Now let's consider the particular functions you can call.

5.1 Adding Hydrogens

Call the `addHydrogens()` function to add missing hydrogen atoms:

```
modeller.addHydrogens(forcefield)
```

The force field is needed to determine the positions for the hydrogen atoms. If the system already contains some hydrogens but is missing others, that is fine. The Modeller will recognize the existing ones and figure out which ones need to be added.

Some residues can exist in different protonation states depending on the pH and on details of the local environment. By default it assumes pH 7, but you can specify a different value:

```
modeller.addHydrogens(forcefield, pH=5.0)
```

For each residue, it selects the protonation state that is most common at the specified pH. In the case of Cysteine residues, it also checks whether the residue participates in a disulfide bond when selecting the state to use. Histidine has two different protonation states that are equally likely at neutral pH. It therefore selects which one to use based on which will form a better hydrogen bond.

If you want more control, it is possible to specify exactly which protonation state to use for particular residues. For details, consult the API documentation for the Modeller class.

5.2 Adding Solvent

Call `addSolvent()` to create a box of solvent (water and ions) around the model:

```
modeller.addSolvent(forcefield)
```

This constructs a box of water around the solute, ensuring that no water molecule comes closer to any solute atom than the sum of their van der Waals radii. It also determines the charge of the solute, and adds enough positive or negative ions to make the system neutral.

When called as shown above, `addSolvent()` expects that periodic box dimensions were specified in the PDB file, and it uses them as the size for the water box. If your PDB file does not specify a box size, or if you want to use a different size, you can specify one:

```
modeller.addSolvent(forcefield, boxSize=Vec3(5.0, 3.5, 3.5)*nanometers)
```

This requests a 5 nm by 3.5 nm by 3.5 nm box. For a non-rectangular box, you can specify the three box vectors defining the unit cell:

```
modeller.addSolvent(forcefield, boxVectors=(avec, bvec, cvec))
```

Another option is to specify a padding distance:

```
modeller.addSolvent(forcefield, padding=1.0*nanometers)
```

This determines the largest size of the solute along any axis (x, y, or z). It then creates a cubic box of width (solute size)+2*(padding). The above line guarantees that no part of the solute comes closer than 1 nm to any edge of the box.

Finally, you can specify the exact number of solvent molecules (including both water and ions) to add. This is useful when you want to solvate several different conformations of the same molecule while guaranteeing they all have the same amount of solvent:

```
modeller.addSolvent(forcefield, numAdded=5000)
```

By default, `addSolvent()` creates TIP3P water molecules, but it also supports other water models:

```
modeller.addSolvent(forcefield, model='tip5p')
```

Allowed values for the `model` option are `'tip3p'`, `'tip3pfb'`, `'spce'`, `'tip4pew'`, `'tip4pfb'`, and `'tip5p'`. Be sure to include the single quotes around the value.

Another option is to add extra ion pairs to give a desired total ionic strength. For example:

```
modeller.addSolvent(forcefield, ionicStrength=0.1*molar)
```

This solvates the system with a salt solution whose ionic strength is 0.1 molar. Note that when computing the ionic strength, it does *not* consider the ions that were added to neutralize the solute. It assumes those are bound to the solute and do not contribute to the bulk ionic strength.

By default, Na^+ and Cl^- ions are used, but you can specify different ones using the `positiveIon` and `negativeIon` options. For example, this creates a potassium chloride solution:

```
modeller.addSolvent(forcefield, ionicStrength=0.1*molar, positiveIon='K+')
```

Allowed values for `positiveIon` are `'Cs+'`, `'K+'`, `'Li+'`, `'Na+'`, and `'Rb+'`. Allowed values for `negativeIon` are `'Cl-'`, `'Br-'`, `'F-'`, and `'I-'`. Be sure to include the single quotes around the value. Also be

aware some force fields do not include parameters for all of these ion types, so you need to use types that are supported by your chosen force field.

5.3 Adding or Removing Extra Particles

"Extra particles" are particles that do not represent ordinary atoms. This includes the virtual interaction sites used in many water models, Drude particles, etc. If you are using a force field that involves extra particles, you must add them to the `Topology`. To do this, call:

```
modeller.addExtraParticles(forcefield)
```

This looks at the force field to determine what extra particles are needed, then modifies each residue to include them. This function can remove extra particles as well as adding them.

5.4 Removing Water

Call deleteWater to remove all water molecules from the system:

```
modeller.deleteWater()
```

This is useful, for example, if you want to simulate it with implicit solvent. Be aware, though, that this only removes water molecules, not ions or other small molecules that might be considered "solvent".

5.5 Saving The Results

Once you have finished editing your model, you can immediately use the resulting `Topology` object and atom positions as the input to a `Simulation`. If you plan to simulate it many times, though, it is usually better to save the result to a new PDB file, then use that as the input for the simulations. This avoids the cost of repeating the modelling operations at the start of every simulation, and also ensures that all your simulations are really starting from exactly the same structure.

The following example loads a PDB file, adds missing hydrogens, builds a solvent box around it, performs an energy minimization, and saves the result to a new PDB file.

```
from simtk.openmm.app import *
from simtk.openmm import *
from simtk.unit import *

print('Loading...')
pdb = PDBFile('input.pdb')
forcefield = ForceField('amber99sb.xml', 'tip3p.xml')
modeller = Modeller(pdb.topology, pdb.positions)
print('Adding hydrogens...')
modeller.addHydrogens(forcefield)
print('Adding solvent...')
modeller.addSolvent(forcefield, model='tip3p', padding=1*nanometer)
print('Minimizing...')
system = forcefield.createSystem(modeller.topology, nonbondedMethod=PME)
integrator = VerletIntegrator(0.001*picoseconds)
simulation = Simulation(modeller.topology, system, integrator)
simulation.context.setPositions(modeller.positions)
simulation.minimizeEnergy(maxIterations=100)
print('Saving...')
positions = simulation.context.getState(getPositions=True).getPositions()
PDBFile.writeFile(simulation.topology, positions, open('output.pdb', 'w'))
print('Done')
```

Example 5-2

CHAPTER

SIX

ADVANCED SIMULATION EXAMPLES

In the previous chapter, we looked at some basic scripts for running simulations and saw lots of ways to customize them. If that is all you want to do—run straightforward molecular simulations—you already know everything you need to know. Just use the example scripts and customize them in the ways described in Section *4.6*.

OpenMM can do far more than that. Your script has the full OpenMM API at its disposal, along with all the power of the Python language and libraries. In this chapter, we will consider some examples that illustrate more advanced techniques. Remember that these are still only examples; it would be impossible to give an exhaustive list of everything OpenMM can do. Hopefully they will give you a sense of what is possible, and inspire you to experiment further on your own.

Starting in this section, we will assume some knowledge of programming, as well as familiarity with the OpenMM API. Consult the OpenMM Users Guide and API documentation if you are uncertain about how something works. You can also use the Python `help` command. For example,

```
help(Simulation)
```

will print detailed documentation on the `Simulation` class.

6.1 Simulated Annealing

Here is a very simple example of how to do simulated annealing. The following lines linearly reduce the temperature from 300 K to 0 K in 100 increments, executing 1000 time steps at each temperature:

```
...
simulation.context.setPositions(pdb.positions)
simulation.minimizeEnergy()
for i in range(100):
    integrator.setTemperature(3*(100-i)*kelvin)
    simulation.step(1000)
```

Example 6-1

This code needs very little explanation. The loop is executed 100 times. Each time through, it adjusts the temperature of the `LangevinIntegrator` and then calls `step(1000)` to take 1000 time steps.

6.2 Applying an External Force to Particles: a Spherical Container

In this example, we will simulate a non-periodic system contained inside a spherical container with radius 2 nm. We implement the container by applying a harmonic potential to every particle:

$$E(r) = \begin{cases} 0 & r \le 2 \\ 100(r-2)^2 & r > 2 \end{cases}$$

where r is the distance of the particle from the origin, measured in nm. We can easily do this using OpenMM's `CustomExternalForce` class. This class applies a force to some or all of the particles in the system, where the energy is an arbitrary function of each particle's (x, y, z) coordinates. Here is the code to do it:

```
...
system = forcefield.createSystem(pdb.topology, nonbondedMethod=CutoffNonPeriodic,
        nonbondedCutoff=1*nanometer, constraints=None)
force = CustomExternalForce('100*max(0, r-2)^2; r=sqrt(x*x+y*y+z*z)')
system.addForce(force)
for i in range(system.getNumParticles()):
    force.addParticle(i, [])
integrator = LangevinIntegrator(300*kelvin, 91/picosecond, 0.002*picoseconds)
...
```

Example 6-2

The first thing it does is create a `CustomExternalForce` object and add it to the `System`. The argument to `CustomExternalForce` is a mathematical expression specifying the potential energy of each particle. This can be any function of x, y, and z you want. It also can depend on global or per-particle parameters. A wide variety of restraints, steering forces, shearing forces, etc. can be implemented with this method.

Next it must specify which particles to apply the force to. In this case, we want it to affect every particle in the system, so we loop over them and call `addParticle()` once for each one. The two arguments are the index of the particle to affect, and the list of per-particle parameter values (an empty list in this case). If we had per-particle parameters, such as to make the force stronger for some particles than for others, this is where we would specify them.

Notice that we do all of this immediately after creating the `System`. That is not an arbitrary choice.

Warning: If you add new forces to a `System`, you must do so before creating the `Simulation`. Once you create a `Simulation`, modifying the `System` will have no effect on that `Simulation`.

6.3 Extracting and Reporting Forces (and other data)

OpenMM provides reporters for three output formats: PDB, PDBx/mmCIF and DCD. All of those formats store only positions, not velocities, forces, or other data. In this section, we create a new reporter that outputs forces. This illustrates two important things: how to write a reporter, and how to query the simulation for forces or other data.

Here is the definition of the `ForceReporter` class:

```
class ForceReporter(object):
    def __init__(self, file, reportInterval):
        self._out = open(file, 'w')
        self._reportInterval = reportInterval

    def __del__(self):
        self._out.close()

    def describeNextReport(self, simulation):
        steps = self._reportInterval - simulation.currentStep%self._reportInterval
        return (steps, False, False, True, False)

    def report(self, simulation, state):
        forces = state.getForces().value_in_unit(kilojoules/mole/nanometer)
        for f in forces:
            self._out.write('%g %g %g\n' % (f[0], f[1], f[2]))
```

Example 6-3

The constructor and destructor are straightforward. The arguments to the constructor are the output filename and the interval (in time steps) at which it should generate reports. It opens the output file for writing and records the reporting interval. The destructor closes the file.

We then have two methods that every reporter must implement: `describeNextReport()` and `report()`. A Simulation object periodically calls `describeNextReport()` on each of its reporters to find out when that reporter will next generate a report, and what information will be needed to generate it. The return value should be a five element tuple, whose elements are as follows:

- The number of time steps until the next report. We calculate this as *(report interval)-(current step)%(report interval)*. For example, if we want a report every 100 steps and the simulation is currently on step 530, we will return 100-(530%100) = 70.
- Whether the next report will need particle positions.
- Whether the next report will need particle velocities.
- Whether the next report will need forces.
- Whether the next report will need energies.

When the time comes for the next scheduled report, the `Simulation` calls `report()` to generate the report. The arguments are the `Simulation` object, and a `State` that is guaranteed to contain all the information that was requested by `describeNextReport()`. A State object contains a snapshot of information about the simulation, such as forces or particle positions. We call `getForces()` to retrieve the forces and convert them to the units we want to output (kJ/mole/nm). Then we loop over each value and write it to the file. To keep the example simple, we just print the values in text format, one line per particle. In a real program, you might choose a different output format.

Now that we have defined this class, we can use it exactly like any other reporter. For example,

```
simulation.reporters.append(ForceReporter('forces.txt', 100))
```

will output forces to a file called "forces.txt" every 100 time steps.

6.4 Computing Energies

This example illustrates a different sort of analysis. Instead of running a simulation, assume we have already identified a set of structures we are interested in. These structures are saved in a set of PDB files. We want to loop over all the

files in a directory, load them in one at a time, and compute the potential energy of each one. Assume we have already created our `System` and `Simulation`. The following lines perform the analysis:

```
import os
for file in os.listdir('structures'):
    pdb = PDBFile(os.path.join('structures', file))
    simulation.context.setPositions(pdb.positions)
    state = simulation.context.getState(getEnergy=True)
    print(file, state.getPotentialEnergy())
```

Example 6-4

We use Python's `listdir()` function to list all the files in the directory. We create a `PDBFile` object for each one and call `setPositions()` on the Context to specify the particle positions loaded from the PDB file. We then compute the energy by calling `getState()` with the option `getEnergy=True`, and print it to the console along with the name of the file.

CHAPTER

SEVEN

CREATING FORCE FIELDS

OpenMM uses a simple XML file format to describe force fields. It includes many common force fields, but you can also create your own. A force field can use all the standard OpenMM force classes, as well as the very flexible custom force classes. You can even extend the ForceField class to add support for completely new forces, such as ones defined in plugins. This makes it a powerful tool for force field development.

7.1 Basic Concepts

Let's start by considering how OpenMM defines a force field. There are a small number of basic concepts to understand.

7.1.1 Atom Types and Atom Classes

Force field parameters are assigned to atoms based on their "atom types". Atom types should be the most specific identification of an atom that will ever be needed. Two atoms should have the same type only if the force field will always treat them identically in every way.

Multiple atom types can be grouped together into "atom classes". In general, two types should be in the same class if the force field usually (but not necessarily always) treats them identically. For example, the α-carbon of an alanine residue will probably have a different atom type than the α-carbon of a leucine residue, but both of them will probably have the same atom class.

All force field parameters can be specified either by atom type or atom class. Classes exist as a convenience to make force field definitions more compact. If necessary, you could define everything in terms of atom types, but when many types all share the same parameters, it is convenient to only have to specify them once.

7.1.2 Residue Templates

Types are assigned to atoms by matching residues to templates. A template specifies a list of atoms, the type of each one, and the bonds between them. For each residue in the PDB file, the force field searches its list of templates for one that has an identical set of atoms with identical bonds between them. When matching templates, neither the order of the atoms nor their names matter; it only cares about their elements and the set of bonds between them. (The PDB file reader does care about names, of course, since it needs to figure out which atom each line of the file corresponds to.)

7.1.3 Forces

Once a force field has defined its atom types and residue templates, it must define its force field parameters. This generally involves one block of XML for each Force object that will be added to the System. The details are different for each Force, but it generally consists of a set of rules for adding interactions based on bonds and atom types

or classes. For example, when adding a HarmonicBondForce, the force field will loop over every pair of bonded atoms, check their types and classes, and see if they match any of its rules. If so, it will call `addBond()` on the HarmonicBondForce. If none of them match, it simply ignores that pair and continues.

7.2 Writing the XML File

The root element of the XML file must be a `<ForceField>` tag:

```
<ForceField>
...
</ForceField>
```

The `<ForceField>` tag contains the following children:

- An `<AtomTypes>` tag containing the atom type definitions
- A `<Residues>` tag containing the residue template definitions
- Zero or more tags defining specific forces

The order of these tags does not matter. They are described in detail below.

7.2.1 <AtomTypes>

The atom type definitions look like this:

```
<AtomTypes>
 <Type name="0" class="N" element="N" mass="14.00672"/>
 <Type name="1" class="H" element="H" mass="1.007947"/>
 <Type name="2" class="CT" element="C" mass="12.01078"/>
 ...
</AtomTypes>
```

There is one `<Type>` tag for each atom type. It specifies the name of the type, the name of the class it belongs to, the symbol for its element, and its mass in amu. The names are arbitrary strings: they need not be numbers, as in this example. The only requirement is that all types have unique names. The classes are also arbitrary strings, and in general will not be unique. Two types belong to the same class if they list the same value for the `class` attribute.

7.2.2 <Residues>

The residue template definitions look like this:

```
<Residues>
 <Residue name="ACE">
  <Atom name="HH31" type="710"/>
  <Atom name="CH3" type="711"/>
  <Atom name="HH32" type="710"/>
  <Atom name="HH33" type="710"/>
  <Atom name="C" type="712"/>
  <Atom name="O" type="713"/>
  <Bond atomName1="HH31" atomName2="CH3"/>
  <Bond atomName1="CH3" atomName2="HH32"/>
  <Bond atomName1="CH3" atomName2="HH33"/>
  <Bond atomName1="CH3" atomName2="C"/>
  <Bond atomName1="C" atomName2="O"/>
  <ExternalBond atomName="C"/>
```

```xml
  </Residue>
  <Residue name="ALA">
   ...
  </Residue>
  ...
</Residues>
```

There is one <Residue> tag for each residue template. That in turn contains the following tags:

- An <Atom> tag for each atom in the residue. This specifies the name of the atom and its atom type.
- A <Bond> tag for each pair of atoms that are bonded to each other. The `atomName1` and `atomName2` attributes are the names of the two bonded atoms. (Some older force fields use the alternate tags `to` and `from` to specify the atoms by index instead of name. This is still supported for backward compatibility, but specifying atoms by name is recommended, since it makes the residue definition much easier to understand.)
- An <ExternalBond> tag for each atom that will be bonded to an atom of a different residue. `atomName` is the name of the atom. (Alternatively, the deprecated `from` tag may indicate the atom by index instead of name.)

The <Residue> tag may also contain <VirtualSite> tags, as in the following example:

```xml
<Residue name="HOH">
  <Atom name="O" type="tip4pew-O"/>
  <Atom name="H1" type="tip4pew-H"/>
  <Atom name="H2" type="tip4pew-H"/>
  <Atom name="M" type="tip4pew-M"/>
  <VirtualSite type="average3" siteName="M" atomName1="O" atomName2="H1" atomName3="H2"
      weight1="0.786646558" weight2="0.106676721" weight3="0.106676721"/>
  <Bond atomName1="O" atomName2="H1"/>
  <Bond atomName1="O" atomName2="H2"/>
</Residue>
```

Each <VirtualSite> tag indicates an atom in the residue that should be represented with a virtual site. The `type` attribute may equal "`average2`", "`average3`", "`outOfPlane`", or "`localCoords`", which correspond to the TwoParticleAverageSite, ThreeParticleAverageSite, OutOfPlaneSite, and LocalCoordinatesSite classes respectively. The `siteName` attribute gives the name of the atom to represent with a virtual site. The atoms it is calculated based on are specified by `atomName1`, `atomName2`, and (for virtual site classes that involve three atoms) `atomName3`. (Some old force fields use the deprecated tags `index`, `atom1`, `atom2`, and `atom3` to refer to them by index instead of name.)

The remaining attributes are specific to the virtual site class, and specify the parameters for calculating the site position. For a TwoParticleAverageSite, they are `weight1` and `weight2`. For a ThreeParticleAverageSite, they are `weight1`, `weight2`, and `weight3`. For an OutOfPlaneSite, they are `weight12`, `weight13`, and `weightCross`. For a LocalCoordinatesSite, they are `wo1`, `wo2`, `wo3`, `wx1`, `wx2`, `wx3`, `wy1`, `wy2`, `wy3`, `p1`, `p2`, and `p3`.

7.2.3 Missing residue templates

> **Caution:** These features are experimental, and their API is subject to change.

You can use the `getUnmatchedResidues()` method to get a list of residues in the provided `topology` object that do not currently have a matching residue template defined in the `ForceField`.

```python
pdb = PDBFile('input.pdb')
forcefield = ForceField('amber99sb.xml', 'tip3p.xml')
unmatched_residues = forcefield.getUnmatchedResidues(topology)
```

This is useful for identifying issues with prepared systems, debugging issues with residue template definitions, or identifying which additional residues need to be parameterized.

As a convenience for parameterizing new residues, you can also get a list of residues and empty residue templates using `generateTemplatesForUnmatchedResidues()`

```
pdb = PDBFile('input.pdb')
forcefield = ForceField('amber99sb.xml', 'tip3p.xml')
[templates, residues] = forcefield.generateTemplatesForUnmatchedResidues(topology)
# Se the atom types
for template in templates:
    for atom in template.atoms:
        atom.type = ... # set the atom types here
    # Register the template with the forcefield.
    forcefield.registerResidueTemplate(template)
```

If you find that templates seem to be incorrectly matched, another useful function `getMatchingTemplates()` can help you identify which templates are being matched:

```
pdb = PDBFile('input.pdb')
forcefield = ForceField('amber99sb.xml', 'tip3p.xml')
templates = forcefield.getMatchingTemplates(topology)
for (residue, template) in zip(pdb.topology.residues(), templates):
    print("Residue %d %s matched template %s" % (residue.id, residue.name, template.name))
```

7.2.4 <HarmonicBondForce>

To add a HarmonicBondForce to the System, include a tag that looks like this:

```
<HarmonicBondForce>
 <Bond class1="C" class2="C" length="0.1525" k="259408.0"/>
 <Bond class1="C" class2="CA" length="0.1409" k="392459.2"/>
 <Bond class1="C" class2="CB" length="0.1419" k="374049.6"/>
 ...
</HarmonicBondForce>
```

Every `<Bond>` tag defines a rule for creating harmonic bond interactions between atoms. Each tag may identify the atoms either by type (using the attributes `type1` and `type2`) or by class (using the attributes `class1` and `class2`). For every pair of bonded atoms, the force field searches for a rule whose atom types or atom classes match the two atoms. If it finds one, it calls `addBond()` on the HarmonicBondForce with the specified parameters. Otherwise, it ignores that pair and continues. `length` is the equilibrium bond length in nm, and `k` is the spring constant in kJ/mol/nm^2.

7.2.5 <HarmonicAngleForce>

To add a HarmonicAngleForce to the System, include a tag that looks like this:

```
<HarmonicAngleForce>
 <Angle class1="C" class2="C" class3="O" angle="2.094" k="669.44"/>
 <Angle class1="C" class2="C" class3="OH" angle="2.094" k="669.44"/>
 <Angle class1="CA" class2="C" class3="CA" angle="2.094" k="527.184"/>
 ...
</HarmonicAngleForce>
```

Every `<Angle>` tag defines a rule for creating harmonic angle interactions between triplets of atoms. Each tag may identify the atoms either by type (using the attributes `type1`, `type2`, ...) or by class (using the attributes `class1`, `class2`, ...). The force field identifies every set of three atoms in the system where the first is bonded to the second, and the second to the third. For each one, it searches for a rule whose atom types or atom classes match the three atoms. If it finds one, it calls `addAngle()` on the HarmonicAngleForce with the specified parameters. Otherwise, it ignores that set and continues. `angle` is the equilibrium angle in radians, and `k` is the spring constant in kJ/mol/radian^2.

7.2.6 <PeriodicTorsionForce>

To add a PeriodicTorsionForce to the System, include a tag that looks like this:

```
<PeriodicTorsionForce>
 <Proper class1="HC" class2="CT" class3="CT" class4="CT" periodicity1="3" phase1="0.0"
     k1="0.66944"/>
 <Proper class1="HC" class2="CT" class3="CT" class4="HC" periodicity1="3" phase1="0.0"
     k1="0.6276"/>
 ...
 <Improper class1="N" class2="C" class3="CT" class4="O" periodicity1="2"
     phase1="3.14159265359" k1="4.6024"/>
 <Improper class1="N" class2="C" class3="CT" class4="H" periodicity1="2"
     phase1="3.14159265359" k1="4.6024"/>
 ...
</PeriodicTorsionForce>
```

Every child tag defines a rule for creating periodic torsion interactions between sets of four atoms. Each tag may identify the atoms either by type (using the attributes `type1`, `type2`, ...) or by class (using the attributes `class1`, `class2`, ...).

The force field recognizes two different types of torsions: proper and improper. A proper torsion involves four atoms that are bonded in sequence: 1 to 2, 2 to 3, and 3 to 4. An improper torsion involves a central atom and three others that are bonded to it: atoms 2, 3, and 4 are all bonded to atom 1. The force field begins by identifying every set of atoms in the system of each of these types. For each one, it searches for a rule whose atom types or atom classes match the four atoms. If it finds one, it calls `addTorsion()` on the PeriodicTorsionForce with the specified parameters. Otherwise, it ignores that set and continues. `periodicity1` is the periodicity of the torsion, `phase1` is the phase offset in radians, and `k1` is the force constant in kJ/mol.

Each torsion definition can specify multiple periodic torsion terms to add to its atoms. To add a second one, just add three more attributes: `periodicity2`, `phase2`, and `k2`. You can have as many terms as you want. Here is an example of a rule that adds three torsion terms to its atoms:

```
<Proper class1="CT" class2="CT" class3="CT" class4="CT"
    periodicity1="3" phase1="0.0" k1="0.75312"
    periodicity2="2" phase2="3.14159265359" k2="1.046"
    periodicity3="1" phase3="3.14159265359" k3="0.8368"/>
```

You can also use wildcards when defining torsions. To do this, simply leave the type or class name for an atom empty. That will cause it to match any atom. For example, the following definition will match any sequence of atoms where the second atom has class OS and the third has class P:

```
<Proper class1="" class2="OS" class3="P" class4="" periodicity1="3" phase1="0.0" k1="1.046"/>
```

7.2.7 <RBTorsionForce>

To add an RBTorsionForce to the System, include a tag that looks like this:

```
<RBTorsionForce>
 <Proper class1="CT" class2="CT" class3="OS" class4="CT" c0="2.439272" c1="4.807416"
     c2="-0.8368" c3="-6.409888" c4="0" c5="0" />
 <Proper class1="C" class2="N" class3="CT" class4="C" c0="10.46" c1="-3.34720"
     c2="-7.1128" c3="0" c4="0" c5="0" />
 ...
 <Improper class1="N" class2="C" class3="CT" class4="O" c0="0.8368" c1="0"
     c2="-2.76144" c3="0" c4="3.3472" c5="0" />
 <Improper class1="N" class2="C" class3="CT" class4="H" c0="29.288" c1="-8.368"
```

```
    c2="-20.92" c3="0" c4="0" c5="0" />
 ...
</RBTorsionForce>
```

Every child tag defines a rule for creating Ryckaert-Bellemans torsion interactions between sets of four atoms. Each tag may identify the atoms either by type (using the attributes `type1`, `type2`, ...) or by class (using the attributes `class1`, `class2`, ...).

The force field recognizes two different types of torsions: proper and improper. A proper torsion involves four atoms that are bonded in sequence: 1 to 2, 2 to 3, and 3 to 4. An improper torsion involves a central atom and three others that are bonded to it: atoms 2, 3, and 4 are all bonded to atom 1. The force field begins by identifying every set of atoms in the system of each of these types. For each one, it searches for a rule whose atom types or atom classes match the four atoms. If it finds one, it calls `addTorsion()` on the RBTorsionForce with the specified parameters. Otherwise, it ignores that set and continues. The attributes `c0` through `c5` are the coefficients of the terms in the Ryckaert-Bellemans force expression.

You can also use wildcards when defining torsions. To do this, simply leave the type or class name for an atom empty. That will cause it to match any atom. For example, the following definition will match any sequence of atoms where the second atom has class OS and the third has class P:

```
<Proper class1="" class2="OS" class3="P" class4="" c0="2.439272" c1="4.807416"
    c2="-0.8368" c3="-6.409888" c4="0" c5="0" />
```

7.2.8 <CMAPTorsionForce>

To add a CMAPTorsionForce to the System, include a tag that looks like this:

```
<CMAPTorsionForce>
 <Map>
  0.0 0.809 0.951 0.309
  -0.587 -1.0 -0.587 0.309
  0.951 0.809 0.0 -0.809
  -0.951 -0.309 0.587 1.0
 </Map>
 <Torsion map="0" class1="CT" class2="CT" class3="C" class4="N" class5="CT"/>
 <Torsion map="0" class1="N" class2="CT" class3="C" class4="N" class5="CT"/>
 ...
</CMAPTorsionForce>
```

Each `<Map>` tag defines an energy correction map. Its content is the list of energy values in kJ/mole, listed in the correct order for CMAPTorsionForce's `addMap()` method and separated by white space. See the API documentation for details. The size of the map is determined from the number of energy values.

Each `<Torsion>` tag defines a rule for creating CMAP torsion interactions between sets of five atoms. The tag may identify the atoms either by type (using the attributes `type1`, `type2`, ...) or by class (using the attributes `class1`, `class2`, ...). The force field identifies every set of five atoms that are bonded in sequence: 1 to 2, 2 to 3, 3 to 4, and 4 to 5. For each one, it searches for a rule whose atom types or atom classes match the five atoms. If it finds one, it calls `addTorsion()` on the CMAPTorsionForce with the specified parameters. Otherwise, it ignores that set and continues. The first torsion is defined by the sequence of atoms 1-2-3-4, and the second one by atoms 2-3-4-5. `map` is the index of the map to use, starting from 0, in the order they are listed in the file.

You can also use wildcards when defining torsions. To do this, simply leave the type or class name for an atom empty. That will cause it to match any atom. For example, the following definition will match any sequence of five atoms where the middle three have classes CT, C, and N respectively:

```
<Torsion map="0" class1="" class2="CT" class3="C" class4="N" class5=""/>
```

7.2.9 <NonbondedForce>

To add a NonbondedForce to the System, include a tag that looks like this:

```
<NonbondedForce coulomb14scale="0.833333" lj14scale="0.5">
 <Atom type="0" charge="-0.4157" sigma="0.32499" epsilon="0.71128"/>
 <Atom type="1" charge="0.2719" sigma="0.10690" epsilon="0.06568"/>
 <Atom type="2" charge="0.0337" sigma="0.33996" epsilon="0.45772"/>
 ...
</NonbondedForce>
```

The `<NonbondedForce>` tag has two attributes `coulomb14scale` and `lj14scale` that specify the scale factors between pairs of atoms separated by three bonds. After setting the nonbonded parameters for all atoms, the force field calls `createExceptionsFromBonds()` on the NonbondedForce, passing in these scale factors as arguments.

Each `<Atom>` tag specifies the nonbonded parameters for one atom type (specified with the `type` attribute) or atom class (specified with the `class` attribute). It is fine to mix these two methods, having some tags specify a type and others specify a class. However you do it, you must make sure that a unique set of parameters is defined for every atom type. `charge` is measured in units of the proton charge, `sigma` is in nm, and `epsilon` is in kJ/mole.

7.2.10 <GBSAOBCForce>

To add a GBSAOBCForce to the System, include a tag that looks like this:

```
<GBSAOBCForce>
 <Atom type="0" charge="-0.4157" radius="0.1706" scale="0.79"/>
 <Atom type="1" charge="0.2719" radius="0.115" scale="0.85"/>
 <Atom type="2" charge="0.0337" radius="0.19" scale="0.72"/>
 ...
</GBSAOBCForce>
```

Each `<Atom>` tag specifies the OBC parameters for one atom type (specified with the `type` attribute) or atom class (specified with the `class` attribute). It is fine to mix these two methods, having some tags specify a type and others specify a class. However you do it, you must make sure that a unique set of parameters is defined for every atom type. `charge` is measured in units of the proton charge, `radius` is the GBSA radius in nm, and `scale` is the OBC scaling factor.

7.2.11 <CustomBondForce>

To add a CustomBondForce to the System, include a tag that looks like this:

```
<CustomBondForce energy="scale*k*(r-r0)^2">
 <GlobalParameter name="scale" defaultValue="0.5"/>
 <PerBondParameter name="k"/>
 <PerBondParameter name="r0"/>
 <Bond class1="OW" class2="HW" r0="0.09572" k="462750.4"/>
 <Bond class1="HW" class2="HW" r0="0.15136" k="462750.4"/>
 <Bond class1="C" class2="C" r0="0.1525" k="259408.0"/>
 ...
</CustomBondForce>
```

The energy expression for the CustomBondForce is specified by the `energy` attribute. This is a mathematical expression that gives the energy of each bond as a function of its length *r*. It also may depend on an arbitrary list of global or per-bond parameters. Use a `<GlobalParameter>` tag to define a global parameter, and a `<PerBondParameter>` tag to define a per-bond parameter.

Every <Bond> tag defines a rule for creating custom bond interactions between atoms. Each tag may identify the atoms either by type (using the attributes type1 and type2) or by class (using the attributes class1 and class2). For every pair of bonded atoms, the force field searches for a rule whose atom types or atom classes match the two atoms. If it finds one, it calls addBond() on the CustomBondForce. Otherwise, it ignores that pair and continues. The remaining attributes are the values to use for the per-bond parameters. All per-bond parameters must be specified for every <Bond> tag, and the attribute name must match the name of the parameter. For instance, if there is a per-bond parameter with the name "k", then every <Bond> tag must include an attribute called k.

7.2.12 <CustomAngleForce>

To add a CustomAngleForce to the System, include a tag that looks like this:

```
<CustomAngleForce energy="scale*k*(theta-theta0)^2">
 <GlobalParameter name="scale" defaultValue="0.5"/>
 <PerAngleParameter name="k"/>
 <PerAngleParameter name=" theta0"/>
 <Angle class1="HW" class2="OW" class3="HW" theta0="1.824218" k="836.8"/>
 <Angle class1="HW" class2="HW" class3="OW" theta0="2.229483" k="0.0"/>
 <Angle class1="C" class2="C" class3="O" theta0="2.094395" k="669.44"/>
 ...
</CustomAngleForce>
```

The energy expression for the CustomAngleForce is specified by the energy attribute. This is a mathematical expression that gives the energy of each angle as a function of the angle *theta*. It also may depend on an arbitrary list of global or per-angle parameters. Use a <GlobalParameter> tag to define a global parameter, and a <PerAngleParameter> tag to define a per-angle parameter.

Every <Angle> tag defines a rule for creating custom angle interactions between triplets of atoms. Each tag may identify the atoms either by type (using the attributes type1, type2, ...) or by class (using the attributes class1, class2, ...). The force field identifies every set of three atoms in the system where the first is bonded to the second, and the second to the third. For each one, it searches for a rule whose atom types or atom classes match the three atoms. If it finds one, it calls addAngle() on the CustomAngleForce. Otherwise, it ignores that set and continues. The remaining attributes are the values to use for the per-angle parameters. All per-angle parameters must be specified for every <Angle> tag, and the attribute name must match the name of the parameter. For instance, if there is a per-angle parameter with the name "k", then every <Angle> tag must include an attribute called k.

7.2.13 <CustomTorsionForce>

To add a CustomTorsionForce to the System, include a tag that looks like this:

```
<CustomTorsionForce energy="scale*k*(1+cos(per*theta-phase))">
 <GlobalParameter name="scale" defaultValue="1"/>
 <PerTorsionParameter name="k"/>
 <PerTorsionParameter name="per"/>
 <PerTorsionParameter name="phase"/>
 <Proper class1="HC" class2="CT" class3="CT" class4="CT" per="3" phase="0.0" k="0.66944"/>
 <Proper class1="HC" class2="CT" class3="CT" class4="HC" per="3" phase="0.0" k="0.6276"/>
 ...
 <Improper class1="N" class2="C" class3="CT" class4="O" per="2" phase="3.14159265359"
     k="4.6024"/>
 <Improper class1="N" class2="C" class3="CT" class4="H" per="2" phase="3.14159265359"
     k="4.6024"/>
 ...
</CustomTorsionForce>
```

The energy expression for the CustomTorsionForce is specified by the `energy` attribute. This is a mathematical expression that gives the energy of each torsion as a function of the angle *theta*. It also may depend on an arbitrary list of global or per-torsion parameters. Use a `<GlobalParameter>` tag to define a global parameter, and a `<PerTorsionParameter>` tag to define a per-torsion parameter.

Every child tag defines a rule for creating custom torsion interactions between sets of four atoms. Each tag may identify the atoms either by type (using the attributes `type1`, `type2`, ...) or by class (using the attributes `class1`, `class2`, ...).

The force field recognizes two different types of torsions: proper and improper. A proper torsion involves four atoms that are bonded in sequence: 1 to 2, 2 to 3, and 3 to 4. An improper torsion involves a central atom and three others that are bonded to it: atoms 2, 3, and 4 are all bonded to atom 1. The force field begins by identifying every set of atoms in the system of each of these types. For each one, it searches for a rule whose atom types or atom classes match the four atoms. If it finds one, it calls `addTorsion()` on the CustomTorsionForce with the specified parameters. Otherwise, it ignores that set and continues. The remaining attributes are the values to use for the per- torsion parameters. Every `<Torsion>` tag must include one attribute for every per-torsion parameter, and the attribute name must match the name of the parameter.

You can also use wildcards when defining torsions. To do this, simply leave the type or class name for an atom empty. That will cause it to match any atom. For example, the following definition will match any sequence of atoms where the second atom has class OS and the third has class P:

```
<Proper class1="" class2="OS" class3="P" class4="" per="3" phase="0.0" k="0.66944"/>
```

7.2.14 <CustomNonbondedForce>

To add a CustomNonbondedForce to the System, include a tag that looks like this:

```
<CustomNonbondedForce energy="scale*epsilon1*epsilon2*((sigma1+sigma2)/r)^12" bondCutoff="3">
 <GlobalParameter name="scale" defaultValue="1"/>
 <PerParticleParameter name="sigma"/>
 <PerParticleParameter name="epsilon"/>
 <Atom type="0" sigma="0.3249" epsilon="0.7112"/>
 <Atom type="1" sigma="0.1069" epsilon="0.0656"/>
 <Atom type="2" sigma="0.3399" epsilon="0.4577"/>
 ...
</CustomNonbondedForce>
```

The energy expression for the CustomNonbondedForce is specified by the `energy` attribute. This is a mathematical expression that gives the energy of each pairwise interaction as a function of the distance *r*. It also may depend on an arbitrary list of global or per-particle parameters. Use a `<GlobalParameter>` tag to define a global parameter, and a `<PerParticleParameter>` tag to define a per-particle parameter.

Exclusions are created automatically based on the `bondCutoff` attribute. After setting the nonbonded parameters for all atoms, the force field calls `createExclusionsFromBonds()` on the CustomNonbondedForce, passing in this value as its argument. To avoid creating exclusions, set `bondCutoff` to 0.

Each `<Atom>` tag specifies the parameters for one atom type (specified with the `type` attribute) or atom class (specified with the `class` attribute). It is fine to mix these two methods, having some tags specify a type and others specify a class. However you do it, you must make sure that a unique set of parameters is defined for every atom type. The remaining attributes are the values to use for the per-atom parameters. All per-atom parameters must be specified for every `<Atom>` tag, and the attribute name must match the name of the parameter. For instance, if there is a per-atom parameter with the name "radius", then every `<Atom>` tag must include an attribute called `radius`.

CustomNonbondedForce also allows you to define tabulated functions. See section 7.2.18 for details.

7.2.15 <CustomGBForce>

To add a CustomGBForce to the System, include a tag that looks like this:

```
<CustomGBForce>
 <GlobalParameter name="solventDielectric" defaultValue="78.3"/>
 <GlobalParameter name="soluteDielectric" defaultValue="1"/>
 <PerParticleParameter name="charge"/>
 <PerParticleParameter name="radius"/>
 <PerParticleParameter name="scale"/>
 <ComputedValue name="I" type="ParticlePairNoExclusions">
    step(r+sr2-or1)*0.5*(1/L-1/U+0.25*(1/U^2-1/L^2)*(r-sr2*sr2/r)+0.5*log(L/U)/r+C);
    U=r+sr2; C=2*(1/or1-1/L)*step(sr2-r-or1); L=max(or1, D); D=abs(r-sr2); sr2 =
    scale2*or2; or1 = radius1-0.009; or2 = radius2-0.009
 </ComputedValue>
 <ComputedValue name="B" type="SingleParticle">
  1/(1/or-tanh(1*psi-0.8*psi^2+4.85*psi^3)/radius); psi=I*or; or=radius-0.009
 </ComputedValue>
 <EnergyTerm type="SingleParticle">
  28.3919551*(radius+0.14)^2*(radius/B)^6-0.5*138.935456*
          (1/soluteDielectric-1/solventDielectric)*charge^2/B
 </EnergyTerm>
 <EnergyTerm type="ParticlePair">
  -138.935456*(1/soluteDielectric-1/solventDielectric)*charge1*charge2/f;
          f=sqrt(r^2+B1*B2*exp(-r^2/(4*B1*B2)))
 </EnergyTerm>
 <Atom type="0" charge="-0.4157" radius="0.1706" scale="0.79"/>
 <Atom type="1" charge="0.2719" radius="0.115" scale="0.85"/>
 <Atom type="2" charge="0.0337" radius="0.19" scale="0.72"/>
 ...
</CustomGBForce>
```

The above (rather complicated) example defines a generalized Born model that is equivalent to GBSAOBCForce. The definition consists of a set of computed values (defined by `<ComputedValue>` tags) and energy terms (defined by `<EnergyTerm>` tags), each of which is evaluated according to a mathematical expression. See the API documentation for details.

The expressions may depend on an arbitrary list of global or per-atom parameters. Use a `<GlobalParameter>` tag to define a global parameter, and a `<PerAtomParameter>` tag to define a per-atom parameter.

Each `<Atom>` tag specifies the parameters for one atom type (specified with the `type` attribute) or atom class (specified with the `class` attribute). It is fine to mix these two methods, having some tags specify a type and others specify a class. However you do it, you must make sure that a unique set of parameters is defined for every atom type. The remaining attributes are the values to use for the per-atom parameters. All per-atom parameters must be specified for every `<Atom>` tag, and the attribute name must match the name of the parameter. For instance, if there is a per-atom parameter with the name "radius", then every `<Atom>` tag must include an attribute called `radius`.

CustomGBForce also allows you to define tabulated functions. See section *7.2.18* for details.

7.2.16 <CustomManyParticleForce>

To add a CustomManyParticleForce to the System, include a tag that looks like this:

```
<CustomManyParticleForce particlesPerSet="3" permutationMode="UniqueCentralParticle"
    bondCutoff="3" energy="scale*(distance(p1,p2)-r1)*(distance(p1,p3)-r1)">
 <GlobalParameter name="scale" defaultValue="1"/>
 <PerParticleParameter name="r"/>
 <TypeFilter index="0" types="1,2"/>
```

```
  <Atom type="0" r="0.31" filterType="0"/>
  <Atom type="1" r="0.25" filterType="0"/>
  <Atom type="2" r="0.33" filterType="1"/>
  ...
</CustomManyParticleForce>
```

The energy expression for the CustomManyParticleForce is specified by the `energy` attribute. This is a mathematical expression that gives the energy of each pairwise interaction as a function of various particle coordinates, distances, and angles. See the API documentation for details. `particlesPerSet` specifies the number of particles involved in the interaction and `permutationMode` specifies the permutation mode.

The expression may depend on an arbitrary list of global or per-atom parameters. Use a `<GlobalParameter>` tag to define a global parameter, and a `<PerAtomParameter>` tag to define a per-atom parameter.

Exclusions are created automatically based on the `bondCutoff` attribute. After setting the nonbonded parameters for all atoms, the force field calls `createExclusionsFromBonds()` on the CustomManyParticleForce, passing in this value as its argument. To avoid creating exclusions, set `bondCutoff` to 0.

Type filters may be specified with a `<TypeFilter>` tag. The `index` attribute specifies the index of the particle to apply the filter to, and `types` is a comma separated list of allowed types.

Each `<Atom>` tag specifies the parameters for one atom type (specified with the `type` attribute) or atom class (specified with the `class` attribute). It is fine to mix these two methods, having some tags specify a type and others specify a class. However you do it, you must make sure that a unique set of parameters is defined for every atom type. In addition, each `<Atom>` tag must include the `filterType` attribute, which specifies the atom type for use in type filters. The remaining attributes are the values to use for the per-atom parameters. All per-atom parameters must be specified for every `<Atom>` tag, and the attribute name must match the name of the parameter. For instance, if there is a per-atom parameter with the name "radius", then every `<Atom>` tag must include an attribute called `radius`.

CustomManyParticleForce also allows you to define tabulated functions. See section *7.2.18* for details.

7.2.17 Writing Custom Expressions

The custom forces described in this chapter involve user defined algebraic expressions. These expressions are specified as character strings, and may involve a variety of standard operators and mathematical functions.

The following operators are supported: + (add), - (subtract), * (multiply), / (divide), and ^ (power). Parentheses "("and ")" may be used for grouping.

The following standard functions are supported: sqrt, exp, log, sin, cos, sec, csc, tan, cot, asin, acos, atan, sinh, cosh, tanh, erf, erfc, min, max, abs, floor, ceil, step, delta, select. step(x) = 0 if x < 0, 1 otherwise. delta(x) = 1 if x is 0, 0 otherwise. select(x,y,z) = z if x = 0, y otherwise. Some custom forces allow additional functions to be defined from tabulated values.

Numbers may be given in either decimal or exponential form. All of the following are valid numbers: 5, -3.1, 1e6, and 3.12e-2.

The variables that may appear in expressions are specified in the API documentation for each force class. In addition, an expression may be followed by definitions for intermediate values that appear in the expression. A semicolon ";" is used as a delimiter between value definitions. For example, the expression

```
a^2+a*b+b^2; a=a1+a2; b=b1+b2
```

is exactly equivalent to

```
(a1+a2)^2+(a1+a2)*(b1+b2)+(b1+b2)^2
```

The definition of an intermediate value may itself involve other intermediate values. All uses of a value must appear *before* that value's definition.

7.2.18 Tabulated Functions

Some forces, such as CustomNonbondedForce and CustomGBForce, allow you to define tabulated functions. To define a function, include a `<Function>` tag inside the `<CustomNonbondedForce>` or `<CustomGBForce>` tag:

```
<Function name="myfn" type="Continuous1D" min="-5" max="5">
0.983674857694 -0.980096396266 -0.975743130031 -0.970451936613 -0.964027580076
-0.956237458128 -0.946806012846 -0.935409070603 -0.921668554406 -0.905148253645
-0.885351648202 -0.861723159313 -0.833654607012 -0.800499021761 -0.761594155956
-0.716297870199 -0.664036770268 -0.604367777117 -0.537049566998 -0.46211715726
-0.379948962255 -0.291312612452 -0.197375320225 -0.099667994625 0.0
0.099667994625 0.197375320225 0.291312612452 0.379948962255 0.46211715726
0.537049566998 0.604367777117 0.664036770268 0.716297870199 0.761594155956
0.800499021761 0.833654607012 0.861723159313 0.885351648202 0.905148253645
0.921668554406 0.935409070603 0.946806012846 0.956237458128 0.964027580076
0.970451936613 0.975743130031 0.980096396266 0.983674857694 0.986614298151
0.989027402201
</Function>
```

The tag's attributes define the name of the function, the type of function, and the range of values for which it is defined. The required set of attributed depends on the function type:

Type	Required Attributes
Continuous1D	min, max
Continuous2D	xmin, ymin, xmax, ymax, xsize, ysize
Continuous3D	xmin, ymin, zmin, xmax, ymax, zmax, xsize, ysize, zsize
Discrete1D	
Discrete2D	xsize, ysize
Discrete3D	xsize, ysize, zsize

The "min" and "max" attributes define the range of the independent variables for a continuous function. The "size" attributes define the size of the table along each axis. The tabulated values are listed inside the body of the tag, with successive values separated by white space. See the API documentation for more details.

7.2.19 Residue Template Parameters

In forces that use an `<Atom>` tag to define parameters for atom types or classes, there is an alternate mechanism you can also use: defining those parameter values in the residue template. This is useful for situations that come up in certain force fields. For example, `NonbondedForce` and `GBSAOBCForce` each have a `charge` attribute. If you only have to define the charge of each atom type once, that is more convenient and avoids potential bugs. Also, many force fields have a different charge for each atom type, but Lennard-Jones parameters that are the same for all types in a class. It would be preferable not to have to repeat those parameter values many times over.

When writing a residue template, you can add arbitrary additional attributes to each `<Atom>` tag. For example, you might include a `charge` attribute as follows:

```
<Atom name="CA" type="53" charge="0.0381"/>
```

When writing the tag for a force, you can then include a `<UseAttributeFromResidue>` tag inside it. This indicates that a specified attribute should be taken from the residue template. Finally, you simply omit that attribute in the force's own `<Atom>` tags. For example:

```
<NonbondedForce coulomb14scale="0.833333" lj14scale="0.5">
 <UseAttributeFromResidue name="charge"/>
 <Atom class="CX" sigma="0.339966950842" epsilon="0.4577296"/>
 ...
</NonbondedForce>
```

Notice that the `charge` attribute is missing, and that the parameters are specified by class, not by type. This means that sigma and epsilon only need to be specified once for each class. The atom charges, which are different for each type, are taken from the residue template instead.

7.3 Using Multiple Files

If multiple XML files are specified when a ForceField is created, their definitions are combined as follows.

- A file may refer to atom types and classes that it defines, as well as those defined in previous files. It may not refer to ones defined in later files. This means that the order in which files are listed when calling the ForceField constructor is potentially significant.
- Forces that involve per-atom parameters (such as NonbondedForce or GBSAOBCForce) require parameter values to be defined for every atom type. It does not matter which file those types are defined in. For example, files that define explicit water models generally define a small number of atom types, as well as nonbonded parameters for those types. In contrast, files that define implicit solvent models do not define any new atom types, but provide parameters for all the atom types that were defined in the main force field file.
- For other forces, the files are effectively independent. For example, if two files each include a `<HarmonicBondForce>` tag, bonds will be created based on the rules in the first file, and then more bonds will be created based on the rules in the second file. This means you could potentially end up with multiple bonds between a single pair of atoms.

7.4 Extending ForceField

The ForceField class is designed to be modular and extensible. This means you can add support for entirely new force types, such as ones implemented with plugins.

7.4.1 Adding new force types

For every force class, there is a “generator” class that parses the corresponding XML tag, then creates Force objects and adds them to the System. ForceField maintains a map of tag names to generator classes. When a ForceField is created, it scans through the XML files, looks up the generator class for each tag, and asks that class to create a generator object based on it. Then, when you call `createSystem()`, it loops over each of its generators and asks each one to create its Force object. Adding a new Force type therefore is simply a matter of creating a new generator class and adding it to ForceField’s map.

The generator class must define two methods. First, it needs a static method with the following signature to parse the XML tag and create the generator:

```
@staticmethod
def parseElement(element, forcefield):
```

`element` is the XML tag (an xml.etree.ElementTree.Element object) and `forcefield` is the ForceField being created. This method should create a generator and add it to the ForceField:

```
generator = MyForceGenerator()
forcefield._forces.append(generator)
```

It then should parse the information contained in the XML tag and configure the generator based on it.

Second, it must define a method with the following signature:

```
def createForce(self, system, data, nonbondedMethod, nonbondedCutoff, args):
```

When `createSystem()` is called on the ForceField, it first creates the System object, then loops over each of its generators and calls `createForce()` on each one. This method should create the Force object and add it to the System. `data` is a ForceField._SystemData object containing information about the System being created (atom types, bonds, angles, etc.), `system` is the System object, and the remaining arguments are values that were passed to `createSystem()`. To get a better idea of how this works, look at the existing generator classes in forcefield.py.

The generator class may optionally also define a method with the following signature:

```
def postprocessSystem(self, system, data, args):
```

If this method exists, it will be called after all Forces have been created. This gives generators a chance to make additional changes to the System.

Finally, you need to register your class by adding it to ForceField's map:

```
forcefield.parsers['MyForce'] = MyForceGenerator.parseElement
```

The key is the XML tag name, and the value is the static method to use for parsing it.

Now you can simply create a ForceField object as usual. If an XML file contains a `<MyForce>` tag, it will be recognized and processed correctly.

7.4.2 Adding residue template generators

Caution: This feature is experimental, and its API is subject to change.

Typically, when `ForceField` encounters a residue it does not have a template for, it simply raises an `Exception`, since it does not know how to assign atom types for the unknown residue.

However, `ForceField` has an API for registering *residue template generators* that are called when a residue without an existing template is encountered. These generators may create new residue templates that match existing atom types and parameters, or can even create new atom types and new parameters that are added to `ForceField`. This functionality can be useful for adding residue template generators that are able to parameterize small molecules that are not represented in a protein or nucleic acid forcefield, for example, or for creating new residue templates for post-translationally modified residues, covalently-bound ligands, or unnatural amino acids or bases.

To register a new residue template generator named `generator`, simply call the `registerTemplateGenerator()` method on an existing `ForceField` object:

```
forcefield.registerTemplateGenerator(generator)
```

This `generator` function must conform to the following API:

```
def generator(forcefield, residue):
    """
    Parameters
    ----------
    forcefield : simtk.openmm.app.ForceField
        The ForceField object to which residue templates and/or parameters are to be added.
    residue : simtk.openmm.app.Topology.Residue
        The residue topology for which a template is to be generated.

    Returns
    -------
    success : bool
```

```
        If the generator is able to successfully parameterize the residue, `True` is returned.
        If the generator cannot parameterize the residue, it should return `False` and not
        modify `forcefield`.

    The generator should either register a residue template directly with
    `forcefield.registerResidueTemplate(template)` or it should call `forcefield.loadFile(file)`
    to load residue definitions from an ffxml file.

    It can also use the `ForceField` programmatic API to add additional atom types (via
    `forcefield.registerAtomType(parameters)`) or additional parameters.

    """
```

The `ForceField` object will be modified by the residue template generator as residues without previously defined templates are encountered. Because these templates are added to the `ForceField` as new residue types are encountered, subsequent residues will be parameterized using the same residue templates without calling the `generator` again.

CHAPTER

EIGHT

THE OPENMM LIBRARY: INTRODUCTION

8.1 What Is the OpenMM Library?

OpenMM consists of two parts. First, there is a set of libraries for performing many types of computations needed for molecular simulations: force evaluation, numerical integration, energy minimization, etc. These libraries provide an interface targeted at developers of simulation software, allowing them to easily add simulation features to their programs.

Second, there is an "application layer", a set of Python libraries providing a high level interface for running simulations. This layer is targeted at computational biologists or other people who want to run simulations, and who may or may not be programmers.

The first part of this guide focused on the application layer and described how to run simulations with it. We now turn to the lower level libraries. We will assume you are a programmer, that you are writing your own applications, and that you want to add simulation features to those applications. The following chapters describe how to do that with OpenMM.

8.1.1 How to get started

We have provided a number of files that make it easy to get started with OpenMM. Pre-compiled binaries are provided for quickly getting OpenMM onto your computer (See Chapter *3* for set-up instructions). We recommend that you then compile and run some of the tutorial examples, described in Chapter *10*. These highlight key functions within OpenMM and teach you the basic programming concepts for using OpenMM. Once you are ready to begin integrating OpenMM into a specific software package, read through Chapter *13* to see how other software developers have done this.

8.1.2 License

Two different licenses are used for different parts of OpenMM. The public API, the low level API, the reference platform, the CPU platform, and the application layer are all distributed under the MIT license. This is a very permissive license which allows them to be used in almost any way, requiring only that you retain the copyright notice and disclaimer when distributing them.

The CUDA and OpenCL platforms are distributed under the GNU Lesser General Public License (LGPL). This also allows you to use, modify, and distribute them in any way you want, but it requires you to also distribute the source code for your modifications. This restriction applies only to modifications to OpenMM itself; you need not distribute the source code to applications that use it.

OpenMM also uses several pieces of code that were written by other people and are covered by other licenses. All of these licenses are similar in their terms to the MIT license, and do not significantly restrict how OpenMM can be used.

All of these licenses may be found in the "licenses" directory included with OpenMM.

8.2 Design Principles

The design of the OpenMM API is guided by the following principles.

1. The API must support efficient implementations on a variety of architectures.

The most important consequence of this goal is that the API cannot provide direct access to state information (particle positions, velocities, etc.) at all times. On some architectures, accessing this information is expensive. With a GPU, for example, it will be stored in video memory, and must be transferred to main memory before outside code can access it. On a distributed architecture, it might not even be present on the local computer. OpenMM therefore only allows state information to be accessed in bulk, with the understanding that doing so may be a slow operation.

2. The API should be easy to understand and easy to use.

This seems obvious, but it is worth stating as an explicit goal. We are creating OpenMM with the hope that many other people will use it. To achieve that goal, it should be possible for someone to learn it without an enormous amount of effort. An equally important aspect of being "easy to use" is being easy to use *correctly*. A well designed API should minimize the opportunities for a programmer to make mistakes. For both of these reasons, clarity and simplicity are essential.

3. It should be modular and extensible.

We cannot hope to provide every feature any user will ever want. For that reason, it is important that OpenMM be easy to extend. If a user wants to add a new molecular force field, a new thermostat algorithm, or a new hardware platform, the API should make that easy to do.

4. The API should be hardware independent.

Computer architectures are changing rapidly, and it is impossible to predict what hardware platforms might be important to support in the future. One of the goals of OpenMM is to separate the API from the hardware. The developers of a simulation application should be able to write their code once, and have it automatically take advantage of any architecture that OpenMM supports, even architectures that do not yet exist when they write it.

8.3 Choice of Language

Molecular modeling and simulation tools are written in a variety of languages: C, C++, Fortran, Python, TCL, etc. It is important that any of these tools be able to use OpenMM. There are two possible approaches to achieving this goal.

One option is to provide a separate version of the API for each language. These could be created by hand, or generated automatically with a wrapper generator such as SWIG. This would require the API to use only "lowest common denominator" features that can be reasonably supported in all languages. For example, an object oriented API would not be an option, since it could not be cleanly expressed in C or Fortran.

The other option is to provide a single version of the API written in a single language. This would permit a cleaner, simpler API, but also restrict the languages it could be directly called from. For example, a C++ API could not be invoked directly from Fortran or Python.

We have chosen to use a hybrid of these two approaches. OpenMM is based on an object oriented C++ API. This is the primary way to invoke OpenMM, and is the only API that fully exposes all features of the library. We believe this will ultimately produce the best, easiest to use API and create the least work for developers who use it. It does require that any code which directly invokes this API must itself be written in C++, but this should not be a significant burden. Regardless of what language we had chosen, developers would need to write a thin layer for translating between their own application's data model and OpenMM. That layer is the only part which needs to be written in C++.

In addition, we have created wrapper APIs that allow OpenMM to be invoked from other languages. The current release includes wrappers for C, Fortran, and Python. These wrappers support as many features as reasonably possible given the constraints of the particular languages, but some features cannot be fully supported. In particular, writing plug-ins to extend the OpenMM API can only be done in C++.

We are also aware that some features of C++ can easily lead to compatibility and portability problems, and we have tried to avoid those features. In particular, we make minimal use of templates and avoid multiple inheritance altogether. Our goal is to support OpenMM on all major compilers and operating systems.

8.4 Architectural Overview

OpenMM is based on a layered architecture, as shown in the following diagram:

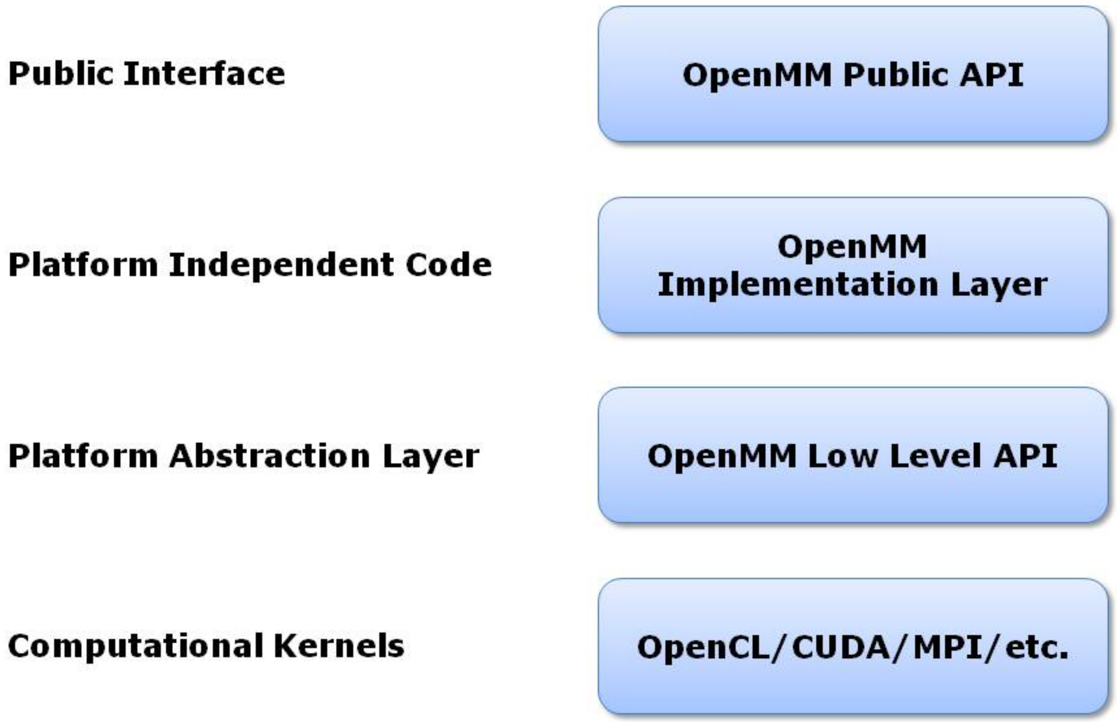

Figure 8-1: OpenMM architecture

At the highest level is the OpenMM public API. This is the API developers program against when using OpenMM within their own applications. It is designed to be simple, easy to understand, and completely platform independent. This is the only layer that many users will ever need to look at.

The public API is implemented by a layer of platform independent code. It serves as the interface to the lower level, platform specific code. Most users will never need to look at it.

The next level down is the OpenMM Low Level API (OLLA). This acts as an abstraction layer to hide the details of each hardware platform. It consists of a set of C++ interfaces that each platform must implement. Users who want to extend OpenMM will need to write classes at the OLLA level. Note the different roles played by the public API and the low level API: the public API defines an interface for users to invoke in their own code, while OLLA defines an interface that users must implement, and that is invoked by the OpenMM implementation layer.

At the lowest level is hardware specific code that actually performs computations. This code may be written in any language and use any technologies that are appropriate. For example, code for GPUs will be written in stream processing languages such as OpenCL or CUDA, code written to run on clusters will use MPI or other distributed computing tools, code written for multicore processors will use threading tools such as Pthreads or OpenMP, etc. OpenMM sets no restrictions on how these computational kernels are written. As long as they are wrapped in the appropriate OLLA interfaces, OpenMM can use them.

8.5 The OpenMM Public API

The public API is based on a small number of classes:

System: A System specifies generic properties of the system to be simulated: the number of particles it contains, the mass of each one, the size of the periodic box, etc. The interactions between the particles are specified through a set of Force objects (see below) that are added to the System. Force field specific parameters, such as particle charges, are not direct properties of the System. They are properties of the Force objects contained within the System.

Force: The Force objects added to a System define the behavior of the particles. Force is an abstract class; subclasses implement specific behaviors. The Force class is actually slightly more general than its name suggests. A Force can, indeed, apply forces to particles, but it can also directly modify particle positions and velocities in arbitrary ways. Some thermostats and barostats, for example, can be implemented as Force classes. Examples of Force subclasses include HarmonicBondForce, NonbondedForce, and MonteCarloBarostat.

Context: This stores all of the state information for a simulation: particle positions and velocities, as well as arbitrary parameters defined by the Forces in the System. It is possible to create multiple Contexts for a single System, and thus have multiple simulations of that System in progress at the same time.

Integrator: This implements an algorithm for advancing the simulation through time. It is an abstract class; subclasses implement specific algorithms. Examples of Integrator subclasses include LangevinIntegrator, VerletIntegrator, and BrownianIntegrator.

State: A State stores a snapshot of the simulation at a particular point in time. It is created by calling a method on a Context. As discussed earlier, this is a potentially expensive operation. This is the only way to query the values of state variables, such as particle positions and velocities; Context does not provide methods for accessing them directly.

Here is an example of what the source code to create a System and run a simulation might look like:

```
System system;
for (int i = 0; i < numParticles; ++i)
    system.addParticle(particle[i].mass);
HarmonicBondForce* bonds = new HarmonicBondForce();
system.addForce(bonds);
for (int i = 0; i < numBonds; ++i)
    bonds->addBond(bond[i].particle1, bond[i].particle2,
        bond[i].length, bond[i].k);
HarmonicAngleForce* angles = new HarmonicAngleForce();
system.addForce(angles);
for (int i = 0; i < numAngles; ++i)
    angles->addAngle(angle[i].particle1, angle[i].particle2,
        angle[i].particle3, angle[i].angle, angle[i].k);
// ...create and initialize other force field terms in the same way
LangevinIntegrator integrator(temperature, friction, stepSize);
Context context(system, integrator);
context.setPositions(initialPositions);
context.setVelocities(initialVelocities);
integrator.step(10000);
```

We create a System, add various Forces to it, and set parameters on both the System and the Forces. We then create a LangevinIntegrator, initialize a Context in which to run a simulation, and instruct the Integrator to advance the simulation for 10,000 time steps.

8.6 The OpenMM Low Level API

The OpenMM Low Level API (OLLA) defines a set of interfaces that users must implement in their own code if they want to extend OpenMM, such as to create a new Force subclass or support a new hardware platform. It is based on the concept of "kernels" that define particular computations to be performed.

More specifically, there is an abstract class called **KernelImpl**. Instances of this class (or rather, of its subclasses) are created by **KernelFactory** objects. These classes provide the concrete implementations of kernels for a particular platform. For example, to perform calculations on a GPU, one would create one or more KernelImpl subclasses that implemented the computations with GPU kernels, and one or more KernelFactory subclasses to instantiate the KernelImpl objects.

All of these objects are encapsulated in a single object that extends **Platform**. KernelFactory objects are registered with the Platform to be used for creating specific named kernels. The choice of what implementation to use (a GPU implementation, a multithreaded CPU implementation, an MPI-based distributed implementation, etc.) consists entirely of choosing what Platform to use.

As discussed so far, the low level API is not in any way specific to molecular simulation; it is a fairly generic computational API. In addition to defining the generic classes, OpenMM also defines abstract subclasses of KernelImpl corresponding to specific calculations. For example, there is a class called CalcHarmonicBondForceKernel to implement HarmonicBondForce and a class called IntegrateLangevinStepKernel to implement LangevinIntegrator. It is these classes for which each Platform must provide a concrete subclass.

This architecture is designed to allow easy extensibility. To support a new hardware platform, for example, you create concrete subclasses of all the abstract kernel classes, then create appropriate factories and a Platform subclass to bind everything together. Any program that uses OpenMM can then use your implementation simply by specifying your Platform subclass as the platform to use.

Alternatively, you might want to create a new Force subclass to implement a new type of interaction. To do this, define an abstract KernelImpl subclass corresponding to the new force, then write the Force class to use it. Any Platform can support the new Force by providing a concrete implementation of your KernelImpl subclass. Furthermore, you can easily provide that implementation yourself, even for existing Platforms created by other people. Simply create a new KernelFactory subclass for your kernel and register it with the Platform object. The goal is to have a completely modular system. Each module, which might be distributed as an independent library, can either add new features to existing platforms or support existing features on new platforms.

In fact, there is nothing "special" about the kernel classes defined by OpenMM. They are simply KernelImpl subclasses that happen to be used by Forces and Integrators that happen to be bundled with OpenMM. They are treated exactly like any other KernelImpl, including the ones you define yourself.

It is important to understand that OLLA defines an interface, not an implementation. It would be easy to assume a one-to-one correspondence between KernelImpl objects and the pieces of code that actually perform calculations, but that need not be the case. For a GPU implementation, for example, a single KernelImpl might invoke several GPU kernels. Alternatively, a single GPU kernel might perform the calculations of several KernelImpl subclasses.

8.7 Platforms

This release of OpenMM contains the following Platform subclasses:

ReferencePlatform: This is designed to serve as reference code for writing other platforms. It is written with simplicity and clarity in mind, not performance.

CpuPlatform: This platform provides high performance when running on conventional CPUs.

CudaPlatform: This platform is implemented using the CUDA language, and performs calculations on Nvidia GPUs.

OpenCLPlatform: This platform is implemented using the OpenCL language, and performs calculations on a variety of types of GPUs and CPUs.

The choice of which platform to use for a simulation depends on various factors:

1. The Reference platform is much slower than the others, and therefore is rarely used for production simulations.
2. The CPU platform is usually the fastest choice when a fast GPU is not available. However, it requires the CPU to support SSE 4.1. That includes most CPUs made in the last several years, but this platform may not be available on some older computers. Also, for simulations that use certain features (primarily the various "custom" force classes), it may be faster to use the OpenCL platform running on the CPU.
3. The CUDA platform can only be used with NVIDIA GPUs. For using an AMD or Intel GPU, use the OpenCL platform.
4. The AMOEBA force field only works with the CUDA platform, not with the OpenCL platform. It also works with the Reference and CPU platforms, but the performance is usually too slow to be useful on those platforms.

CHAPTER

NINE

COMPILING OPENMM FROM SOURCE CODE

This chapter describes the procedure for building and installing OpenMM libraries from source code. It is recommended that you use binary OpenMM libraries, if possible. If there are not suitable binary libraries for your system, consider building OpenMM from source code by following these instructions.

9.1 Prerequisites

Before building OpenMM from source, you will need the following:

- A C++ compiler
- CMake
- OpenMM source code

See the sections below for specific instructions for the different platforms.

9.1.1 Get a C++ compiler

You must have a C++ compiler installed before attempting to build OpenMM from source.

Mac and Linux: clang or gcc

Use clang or gcc on Mac/Linux. OpenMM should compile correctly with all recent versions of these compilers. We recommend clang since it produces faster code, especially when using the CPU platform. If you do not already have a compiler installed, you will need to download and install it. On Mac OS X, this means downloading the Xcode Tools from the App Store.

Windows: Visual Studio

On Windows systems, use the C++ compiler in Visual Studio version 10 (2010) or later. You can download a free version of Visual C++ Express Edition from http://www.microsoft.com/express/vc/. If you plan to use use OpenMM from Python, it is critical that both OpenMM and Python be compiled with the same version of Visual Studio.

9.1.2 Install CMake

CMake is the build system used for OpenMM. You must install CMake version 2.8 or higher before attempting to build OpenMM from source. You can get CMake from http://www.cmake.org/. If you choose to build CMake from source

on Linux, make sure you have the curses library installed beforehand, so that you will be able to build the CCMake visual CMake tool.

9.1.3 Get the OpenMM source code

You will also need the OpenMM source code before building OpenMM from source. To download and unpack OpenMM source code:

1. Browse to https://simtk.org/home/openmm.
2. Click the "Downloads" link in the navigation bar on the left side.
3. Download OpenMM<Version>-Source.zip, choosing the latest version.
4. Unpack the zip file. Note the location where you unpacked the OpenMM source code.

Alternatively, if you want the most recent development version of the code rather than the version corresponding to a particular release, you can get it from https://github.com/pandegroup/openmm. Be aware that the development code is constantly changing, may contain bugs, and should never be used for production work. If you want a stable, well tested version of OpenMM, you should download the source code for the latest release as described above.

9.1.4 Other Required Software

There are several other pieces of software you must install to compile certain parts of OpenMM. Which of these you need depends on the options you select in CMake.

- For compiling the CUDA Platform, you need:
  - CUDA (See Chapter *3* for installation instructions.)
- For compiling the OpenCL Platform, you need:
  - OpenCL (See Chapter *3* for installation instructions.)
- For compiling C and Fortran API wrappers, you need:
  - Python 2.6 or later (http://www.python.org)
  - Doxygen (http://www.doxygen.org)
  - A Fortran compiler
- For compiling the Python API wrappers, you need:
  - Python 2.6 or later (http://www.python.org)
  - SWIG (http://www.swig.org)
  - Doxygen (http://www.doxygen.org)
- For compiling the CPU platform, you need:
  - FFTW, single precision multithreaded version (http://www.fftw.org)
- To generate API documentation, you need:
  - Doxygen (http://www.doxygen.org)

9.2 Step 1: Configure with CMake

9.2.1 Build and source directories

First, create a directory in which to build OpenMM. A good name for this directory is build_openmm. We will refer to this as the "build_openmm directory" in the instructions below. This directory will contain the temporary files used by the OpenMM CMake build system. Do not create this build directory within the OpenMM source code directory. This is what is called an "out of source" build, because the build files will not be mixed with the source files.

Also note the location of the OpenMM source directory (i.e., where you unpacked the source code zip file). It should contain a file called CMakeLists.txt. This directory is what we will call the "OpenMM source directory" in the following instructions.

9.2.2 Starting CMake

Configuration is the first step of the CMake build process. In the configuration step, the values of important build variables will be established.

Mac and Linux

On Mac and Linux machines, type the following two lines:

```
cd build_openmm
ccmake -i <path to OpenMM src directory>
```

That is not a typo. `ccmake` has two c's. CCMake is the visual CMake configuration tool. Press "`c`" within the CCMake interface to configure CMake. Follow the instructions in the "All Platforms" section below.

Windows

On Windows, perform the following steps:

1. Click Start->All Programs->CMake 2.8->CMake
2. In the box labeled "Where is the source code:" browse to OpenMM src directory (containing top CMakeLists.txt)
3. In the box labeled "Where to build the binaries" browse to your build_openmm directory.
4. Click the "Configure" button at the bottom of the CMake screen.
5. Select "Visual Studio 10 2010" from the list of Generators (or whichever version you have installed)
6. Follow the instructions in the "All Platforms" section below.

All platforms

There are several variables that can be adjusted in the CMake interface:

- If you intend to use CUDA (NVIDIA) or OpenCL acceleration, set the variable OPENMM_BUILD_CUDA_LIB or OPENMM_BUILD_OPENCL_LIB, respectively, to ON. Before doing so, be certain that you have installed and tested the drivers for the platform you have selected (see Chapter *3* for information on installing GPU software).

- There are lots of other options starting with OPENMM_BUILD that control whether to build particular features of OpenMM, such as plugins, API wrappers, and documentation.
- Set the variable CMAKE_INSTALL_PREFIX to the location where you want to install OpenMM.
- Set the variable PYTHON_EXECUTABLE to the Python interpreter you plan to use OpenMM with.

Configure (press "c") again. Adjust any variables that cause an error.

Continue to configure (press "c") until no starred/red CMake variables are displayed. Congratulations, you have completed the configuration step.

9.3 Step 2: Generate Build Files with CMake

Once the configuration is done, the next step is generation. The generate "g" or "OK" or "Generate" option will not be available until configuration has completely converged.

9.3.1 Windows

- Press the "OK" or "Generate" button to generate Visual Studio project files.
- If CMake does not exit automatically, press the close button in the upper- right corner of the CMake title bar to exit.

9.3.2 Mac and Linux

- Press "g" to generate the Makefile.
- If CMake does not exit automatically, press "q" to exit.

That's it! Generation is the easy part. Now it's time to build.

9.4 Step 3: Build OpenMM

9.4.1 Windows

1. Open the file OpenMM.sln in your openmm_build directory in Visual Studio.
2. Set the configuration type to "Release" (not "Debug") in the toolbar.
3. From the Build menu, click Build->Build Solution
4. The OpenMM libraries and test programs will be created. This takes some time.
5. The test program TestCudaRandom might not build on Windows. This is OK.

9.4.2 Mac and Linux

- Type `make` in the openmm_build directory.

The OpenMM libraries and test programs will be created. This takes some time.

9.5 Step 4: Install OpenMM

9.5.1 Windows

In the Solution Explorer Panel, far-click/right-click INSTALL->build.

9.5.2 Mac and Linux

Type:

```
make install
```

If you are installing to a system area, such as /usr/local/openmm/, you will need to type:

```
sudo make install
```

9.6 Step 5: Install the Python API

9.6.1 Windows

In the Solution Explorer Panel, right-click PythonInstall->build.

9.6.2 Mac and Linux

Type:

```
make PythonInstall
```

If you are installing into the system Python, such as /usr/bin/python, you will need to type:

```
sudo make PythonInstall
```

9.7 Step 6: Test your build

After OpenMM has been built, you should run the unit tests to make sure it works.

9.7.1 Windows

In Visual Studio, far-click/right-click RUN_TESTS in the Solution Explorer Panel. Select RUN_TESTS->build to begin testing. Ignore any failures for TestCudaRandom.

9.7.2 Mac and Linux

Type:

```
make test
```

You should see a series of test results like this:

```
      Start   1: TestReferenceAndersenThermostat
1/317 Test   #1: TestReferenceAndersenThermostat .............. Passed  0.26 sec
      Start   2: TestReferenceBrownianIntegrator
2/317 Test   #2: TestReferenceBrownianIntegrator .............. Passed  0.13 sec
      Start   3: TestReferenceCheckpoints
3/317 Test   #3: TestReferenceCheckpoints ..................... Passed  0.02 sec
... <many other tests> ...
```

`Passed` is good. `FAILED` is bad. If any tests fail, you can run them individually to get more detailed error information. Note that some tests are stochastic, and therefore are expected to fail a small fraction of the time. These tests will say so in the error message:

```
./TestReferenceLangevinIntegrator

exception: Assertion failure at TestReferenceLangevinIntegrator.cpp:129.  Expected 9.97741,
    found 10.7884 (This test is stochastic and may occasionally fail)
```

Congratulations! You successfully have built and installed OpenMM from source.

9.8 Building the Documentation (Optional)

The documentation that you're currently reading, as well as the developer guide and API documentation can be built through CMake by setting the OpenMM option `OPENMM_GENERATE_API_DOCS=ON`.

9.8.1 User Guide and Developer Guide

Generating the user guide and developer guide requires the following dependencies

- Sphinx (http://sphinx-doc.org/)
- sphinxcontrib-bibtex (https://pypi.python.org/pypi/sphinxcontrib-bibtex)

These dependencies may not be available in your system package manager, but should be installable through Python's `pip` package manager.

```
pip install sphinx sphinxcontrib-bibtex
```

The developer and user guides can be built either as HTML or a PDFs. Building the PDF version will also require a functional LaTeX installation.

To build the HTML version of the documentation, type:

```
make sphinxhtml
```

To build the PDF version of the documentation, type:

```
make sphinxpdf
```

9.8.2 Python and C++ API Documentation

The following dependencies are required to build the Python and C++ API documentation.

- Sphinx (http://sphinx-doc.org/)
- sphinxcontrib-lunrsearch (https://pypi.python.org/pypi/sphinxcontrib-lunrsearch)
- sphinxcontrib-autodoc_doxygen (https://pypi.python.org/pypi/sphinxcontrib-autodoc_doxygen)

These dependencies may not be available in your system package manager, but should be installable through Python's `pip` package manager.

```
pip install sphinx sphinxcontrib-lunrsearch sphinxcontrib-autodoc_doxygen
```

To build the C++ API documentation, type:

```
make C++ApiDocs
```

To build the Python API documentation, type:

```
make PythonApiDocs
```

CHAPTER

TEN

OPENMM TUTORIALS

10.1 Example Files Overview

Four example files are provided in the examples folder, each designed with a specific objective.

- **HelloArgon:** A very simple example intended for verifying that you have installed OpenMM correctly. It also introduces you to the basic classes within OpenMM.
- **HelloSodiumChloride:** This example shows you our recommended strategy for integrating OpenMM into an existing molecular dynamics code.
- **HelloEthane:** The main purpose of this example is to demonstrate how to tell OpenMM about bonded forces (bond stretch, bond angle bend, dihedral torsion).
- **HelloWaterBox:** This example shows you how to use OpenMM to model explicit solvation, including setting up periodic boundary conditions. It runs extremely fast on a GPU but very, very slowly on a CPU, so it is an excellent example to use to compare performance on the GPU versus the CPU. The other examples provided use systems where the performance difference would be too small to notice.

The two fundamental examples—HelloArgon and HelloSodiumChloride—are provided in C++, C, and Fortran, as indicated in the table below. The other two examples—HelloEthane and HelloWaterBox—follow the same structure as HelloSodiumChloride but demonstrate more calls within the OpenMM API. They are only provided in C++ but can be adapted to run in C and Fortran by following the mappings described in Chapter *12*. HelloArgon and HelloSodiumChloride also serve as examples of how to do these mappings. The sections below describe the HelloArgon, HelloSodiumChloride, and HelloEthane programs in more detail.

Example	Solvent	Thermostat	Boundary	Forces & Constraints	API
Argon	Vacuum	None	None	Non-bonded*	C++, C, Fortran
Sodium Chloride	Implicit water	Langevin	None	Non-bonded*	C++, C, Fortran
Ethane	Vacuum	None	None	Non-bonded*, stretch, bend, torsion	C++
Water Box	Explicit water	Andersen	Periodic	Non-bonded*, stretch, bend, constraints	C++

*van der Waals and Coulomb forces

10.2 Running Example Files

The instructions below are for running the HelloArgon program. A similar process would be used to run the other examples.

10.2.1 Visual Studio

Navigate to wherever you saved the example files. Descend into the directory folder VisualStudio. Double-click the file HelloArgon.sln (a Microsoft Visual Studio Solution file). Visual Studio will launch.

Note: These files were created using Visual Studio 8. If you are using a more recent version, it will ask if you want to convert the files to the new version. Agree and continue through the conversion process.

In Visual Studio, make sure the "Solution Configuration" is set to "Release" and not "Debug". The "Solution Configuration" can be set using the drop-down menu in the top toolbar, next to the green arrow (see Figure 10-1 below). Due to incompatibilities among Visual Studio versions, we do not provide pre-compiled debug binaries.

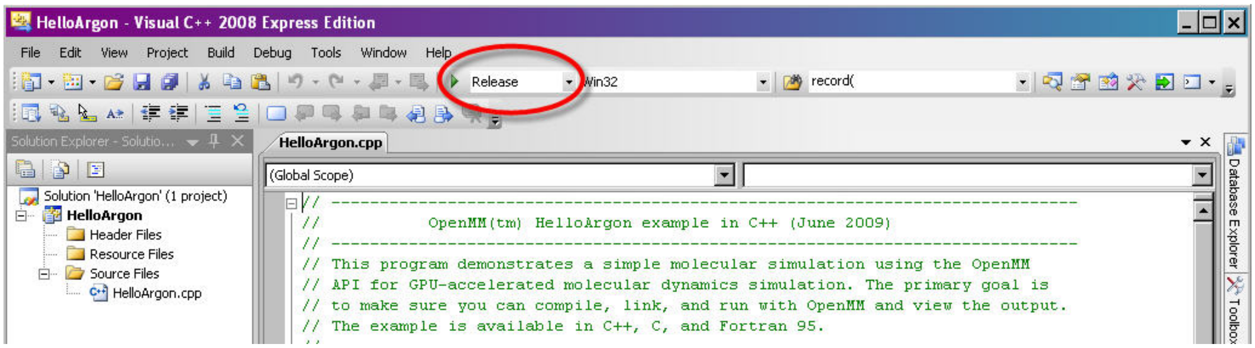

Figure 10-1: Setting "Solution Configuration" to "Release" mode in Visual Studio

From the command options select Debug -> Start Without Debugging (or CTRL-F5). See Figure 10-2. This will also compile the program, if it has not previously been compiled.

You should see a series of lines like the following output on your screen:

```
REMARK  Using OpenMM platform Reference
MODEL     1
ATOM      1  AR   AR     1       0.000   0.000   0.000  1.00  0.00
ATOM      2  AR   AR     1       5.000   0.000   0.000  1.00  0.00
ATOM      3  AR   AR     1       10.000  0.000   0.000  1.00  0.00
ENDMDL

...

MODEL     250
ATOM      1  AR   AR     1       0.233   0.000   0.000  1.00  0.00
ATOM      2  AR   AR     1       5.068   0.000   0.000  1.00  0.00
ATOM      3  AR   AR     1       9.678   0.000   0.000  1.00  0.00
ENDMDL
MODEL     251
ATOM      1  AR   AR     1       0.198   0.000   0.000  1.00  0.00
ATOM      2  AR   AR     1       5.082   0.000   0.000  1.00  0.00
ATOM      3  AR   AR     1       9.698   0.000   0.000  1.00  0.00
ENDMDL
MODEL     252
ATOM      1  AR   AR     1       0.165   0.000   0.000  1.00  0.00
ATOM      2  AR   AR     1       5.097   0.000   0.000  1.00  0.00
ATOM      3  AR   AR     1       9.717   0.000   0.000  1.00  0.00
ENDMDL
```

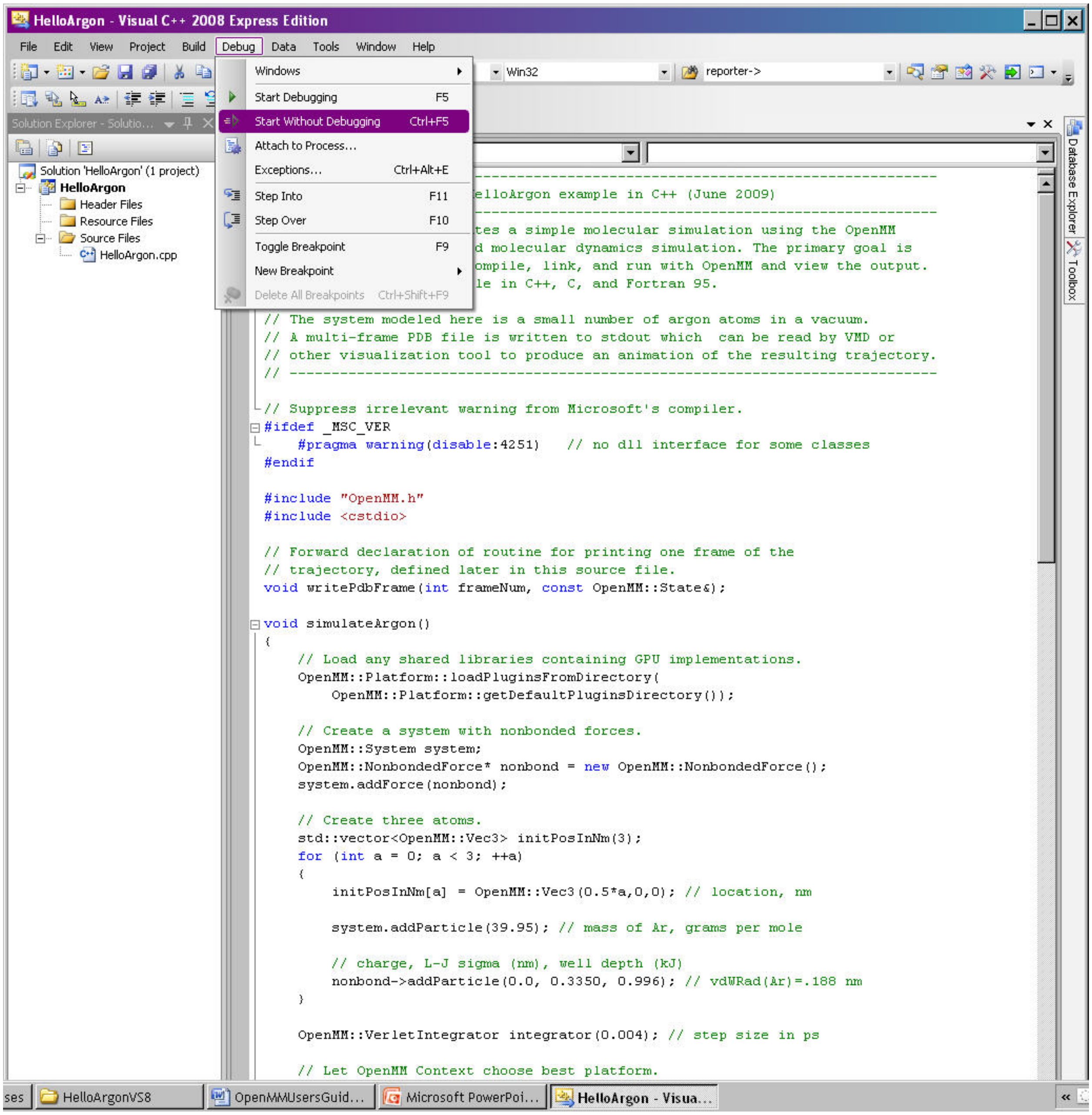

Figure 10-2: Run a program in Visual Studio

Determining the platform being used

The very first line of the output will indicate whether you are running on the CPU (Reference platform) or a GPU (CUDA or OpenCL platform). It will say one of the following:

```
REMARK  Using OpenMM platform Reference
REMARK  Using OpenMM platform Cuda
REMARK  Using OpenMM platform OpenCL
```

If you have a supported GPU, the program should, by default, run on the GPU.

Visualizing the results

You can output the results to a PDB file that could be visualized using programs like VMD (http://www.ks.uiuc.edu/Research/vmd/) or PyMol (http://pymol.sourceforge.net/). To do this within Visual Studios:

1. Right-click on the project name HelloArgon (not one of the files) and select the "Properties" option.
2. On the "Property Pages" form, select "Debugging" under the "Configuration Properties" node.
3. In the "Command Arguments" field, type:

```
     > argon.pdb
```

   This will save the output to a file called argon.pdb in the current working directory (default is the VisualStudio directory). If you want to save it to another directory, you will need to specify the full path.

4. Select "OK"

Now, when you run the program in Visual Studio, no text will appear. After a short time, you should see the message "`Press any key to continue...`," indicating that the program is complete and that the PDB file has been completely written.

10.2.2 Mac OS X/Linux

Navigate to wherever you saved the example files.

Verify your makefile by consulting the MakefileNotes file in this directory, if necessary.

Type::

```
make
```

Then run the program by typing:

```
./HelloArgon
```

You should see a series of lines like the following output on your screen:

```
REMARK  Using OpenMM platform Reference
MODEL     1
ATOM      1  AR   AR     1       0.000   0.000   0.000  1.00  0.00
ATOM      2  AR   AR     1       5.000   0.000   0.000  1.00  0.00
ATOM      3  AR   AR     1       10.000  0.000   0.000  1.00  0.00
ENDMDL

...
```

```
MODEL     250
ATOM      1  AR   AR     1       0.233   0.000   0.000  1.00  0.00
ATOM      2  AR   AR     1       5.068   0.000   0.000  1.00  0.00
ATOM      3  AR   AR     1       9.678   0.000   0.000  1.00  0.00
ENDMDL
MODEL     251
ATOM      1  AR   AR     1       0.198   0.000   0.000  1.00  0.00
ATOM      2  AR   AR     1       5.082   0.000   0.000  1.00  0.00
ATOM      3  AR   AR     1       9.698   0.000   0.000  1.00  0.00
ENDMDL
MODEL     252
ATOM      1  AR   AR     1       0.165   0.000   0.000  1.00  0.00
ATOM      2  AR   AR     1       5.097   0.000   0.000  1.00  0.00
ATOM      3  AR   AR     1       9.717   0.000   0.000  1.00  0.00
ENDMDL
```

Determining the platform being used

The very first line of the output will indicate whether you are running on the CPU (Reference platform) or a GPU (CUDA or OpenCL platform). It will say one of the following:

```
REMARK  Using OpenMM platform Reference
REMARK  Using OpenMM platform Cuda
REMARK  Using OpenMM platform OpenCL
```

If you have a supported GPU, the program should, by default, run on the GPU.

Visualizing the results

You can output the results to a PDB file that could be visualized using programs like VMD (http://www.ks.uiuc.edu/Research/vmd/) or PyMol (http://pymol.sourceforge.net/) by typing:

```
./HelloArgon > argon.pdb
```

Compiling Fortran and C examples

The Makefile provided with the examples can also be used to compile the Fortran and C examples.

The Fortran compiler needs to load a version of the libstdc++.dylib library that is compatible with the version of gcc used to build OpenMM; OpenMM for Mac is compiled using gcc 4.2. If you are compiling with a different version, edit the Makefile and add the following flag to FCPPLIBS: `-L/usr/lib/gcc/i686 -apple-darwin10/4.2.1`.

When the Makefile has been updated, type:

```
make all
```

10.3 HelloArgon Program

The HelloArgon program simulates three argon atoms in a vacuum. It is a simple program primarily intended for you to verify that you are able to compile, link, and run with OpenMM. It also demonstrates the basic calls needed to run a simulation using OpenMM.

10.3.1 Including OpenMM-defined functions

The OpenMM header file *OpenMM.h* instructs the program to include everything defined by the OpenMM libraries. Include the header file by adding the following line at the top of your program:

```
#include "OpenMM.h"
```

10.3.2 Running a program on GPU platforms

By default, a program will run on the Reference platform. In order to run a program on another platform (e.g., an NVIDIA or AMD GPU), you need to load the required shared libraries for that other platform (e.g., Cuda, OpenCL). The easy way to do this is to call:

```
OpenMM::Platform::loadPluginsFromDirectory(OpenMM::Platform::getDefaultPluginsDirectory());
```

This will load all the shared libraries (plug-ins) that can be found, so you do not need to explicitly know which libraries are available on a given machine. In this way, the program will be able to run on another platform, if it is available.

10.3.3 Running a simulation using the OpenMM public API

The OpenMM public API was described in Section *8.5*. Here you will see how to use those classes to create a simple system of three argon atoms and run a short simulation. The main components of the simulation are within the function `simulateArgon()`:

1. **System** – We first establish a system and add a non-bonded force to it. At this point, there are no particles in the system.

```
// Create a system with nonbonded forces.
OpenMM::System system;
OpenMM::NonbondedForce* nonbond = new OpenMM::NonbondedForce();
system.addForce(nonbond);
```

We then add the three argon atoms to the system. For this system, all the data for the particles are hard-coded into the program. While not a realistic scenario, it makes the example simpler and clearer. The `std::vector<OpenMM::Vec3>` is an array of vectors of 3.

```
// Create three atoms.
std::vector<OpenMM::Vec3> initPosInNm(3);
for (int a = 0; a < 3; ++a)
{
    initPosInNm[a] = OpenMM::Vec3(0.5*a,0,0); // location, nm

    system.addParticle(39.95); // mass of Ar, grams per mole

    // charge, L-J sigma (nm), well depth (kJ)
    nonbond->addParticle(0.0, 0.3350, 0.996); // vdWRad(Ar)=.188 nm
}
```

Units: Be very careful with the units in your program. It is very easy to make mistakes with the units, so we recommend including them in your variable names, as we have done here `initPosInNm` (position in nanometers). OpenMM provides conversion constants that should be used whenever there are conversions to be done; for simplicity, we did not do that in HelloArgon, but all the other examples show the use of these constants.

It is hard to overemphasize the importance of careful units handling—it is very easy to make a mistake despite, or perhaps because of, the trivial nature of units conversion. For more information about the units used in OpenMM, see Section *18.2*.

Adding Particle Information: Both the system and the non-bonded force require information about the particles. The system just needs to know the mass of the particle. The non-bonded force requires information about the charge (in this case, argon is uncharged), and the Lennard-Jones parameters sigma (zero-energy separation distance) and well depth (see Section *19.6.1* for more details).

Note that the van der Waals radius for argon is 0.188 nm and that it has already been converted to sigma (0.335 nm) in the example above where it is added to the non-bonded force; in your code, you should make use of the appropriate conversion factor supplied with OpenMM as discussed in Section *18.2*.

2. **Integrator** – We next specify the integrator to use to perform the calculations. In this case, we choose a Verlet integrator to run a constant energy simulation. The only argument required is the step size in picoseconds.

```
OpenMM::VerletIntegrator integrator(0.004); // step size in ps
```

We have chosen to use 0.004 picoseconds, or 4 femtoseconds, which is larger than that used in a typical molecular dynamics simulation. However, since this example does not have any bonds with higher frequency components, like most molecular dynamics simulations do, this is an acceptable value.

3. **Context** – The context is an object that consists of an integrator and a system. It manages the state of the simulation. The code below initializes the context. We then let the context select the best platform available to run on, since this is not specifically specified, and print out the chosen platform. This is useful information, especially when debugging.

```
// Let OpenMM Context choose best platform.
OpenMM::Context context(system, integrator);
printf("REMARK  Using OpenMM platform %s\n", context.getPlatform().getName().c_str());
```

We then initialize the system, setting the initial time, as well as the initial positions and velocities of the atoms. In this example, we leave time and velocity at their default values of zero.

```
// Set starting positions of the atoms. Leave time and velocity zero.
context.setPositions(initPosInNm);
```

4. **Initialize and run the simulation** – The next block of code runs the simulation and saves its output. For each frame of the simulation (in this example, a frame is defined by the advancement interval of the integrator; see below), the current state of the simulation is obtained and written out to a PDB-formatted file.

```
// Simulate.
for (int frameNum=1; ;++frameNum) {
    // Output current state information.
    OpenMM::State state = context.getState(OpenMM::State::Positions);
    const double  timeInPs = state.getTime();
    writePdbFrame(frameNum, state); // output coordinates
```

Getting state information has to be done in bulk, asking for information for all the particles at once. This is computationally expensive since this information can reside on the GPUs and requires communication overhead to retrieve, so you do not want to do it very often. In the above code, we only request the positions, since that is all that is needed, and time from the state.

The simulation stops after 10 ps; otherwise we ask the integrator to take 10 steps (so one frame is equivalent to 10 time steps). Normally, we would want to take more than 10 steps at a time, but to get a reasonable-looking animation, we use 10.

```
if (timeInPs >= 10.)
    break;

// Advance state many steps at a time, for efficient use of OpenMM.
integrator.step(10); // (use a lot more than this normally)
```

10.3.4 Error handling for OpenMM

Error handling for OpenMM is explicitly designed so you do not have to check the status after every call. If anything goes wrong, OpenMM throws an exception. It uses standard exceptions, so on many platforms, you will get the exception message automatically. However, we recommend using `try-catch` blocks to ensure you do catch the exception.

```
int main()
{
    try {
        simulateArgon();
        return 0; // success!
    }
    // Catch and report usage and runtime errors detected by OpenMM and fail.
    catch(const std::exception& e) {
        printf("EXCEPTION: %s\n", e.what());
        return 1; // failure!
    }
}
```

10.3.5 Writing out PDB files

For the HelloArgon program, we provide a simple PDB file writing function `writePdbFrame` that *only* writes out argon atoms. The function has nothing to do with OpenMM except for using the OpenMM State. The function extracts the positions from the State in nanometers (10^{-9} m) and converts them to Angstroms (10^{-10} m) to be compatible with the PDB format. Again, we emphasize how important it is to track the units being used!

```
void writePdbFrame(int frameNum, const OpenMM::State& state)
{
    // Reference atomic positions in the OpenMM State.
    const std::vector<OpenMM::Vec3>& posInNm = state.getPositions();

    // Use PDB MODEL cards to number trajectory frames
    printf("MODEL     %d\n", frameNum); // start of frame
    for (int a = 0; a < (int)posInNm.size(); ++a)
    {
        printf("ATOM  %5d  AR   AR     1    ", a+1); // atom number
        printf("%8.3f%8.3f%8.3f  1.00  0.00\n",      // coordinates
        // "*10" converts nanometers to Angstroms
        posInNm[a][0]*10, posInNm[a][1]*10, posInNm[a][2]*10);
    }
    printf("ENDMDL\n"); // end of frame
}
```

`MODEL` and `ENDMDL` are used to mark the beginning and end of a frame, respectively. By including multiple frames in a PDB file, you can visualize the simulation trajectory.

10.3.6 HelloArgon output

The output of the HelloArgon program can be saved to a *.pdb* file and visualized using programs like VMD or PyMol (see Section *10.2*). You should see three atoms moving linearly away and towards one another:

You may need to adjust the van der Waals radius in your visualization program to see the atoms colliding.

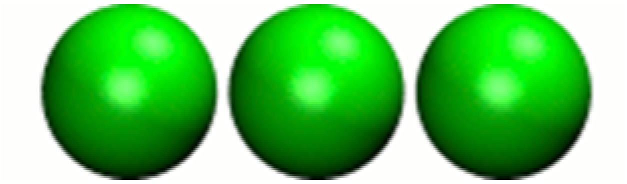

10.4 HelloSodiumChloride Program

The HelloSodiumChloride models several sodium (Na^+) and chloride (Cl^-) ions in implicit solvent (using a Generalized Born/Surface Area, or GBSA, OBC model). As with the HelloArgon program, only non-bonded forces are simulated.

The main purpose of this example is to illustrate our recommended strategy for integrating OpenMM into an existing molecular dynamics (MD) code:

1. **Write a few, high-level interface routines containing all your OpenMM calls**: Rather than make OpenMM calls throughout your program, we recommend writing a handful of interface routines that understand both your MD code's data structures and OpenMM. Organize these routines into a separate compilation unit so you do not have to make huge changes to your existing MD code. These routines could be written in any language that is callable from the existing MD code. We recommend writing them in C++ since that is what OpenMM is written in, but you can also write them in C or Fortran; see Chapter *12*.
2. **Call only these high-level interface routines from your existing MD code:** This provides a clean separation between the existing MD code and OpenMM, so that changes to OpenMM will not directly impact the existing MD code. One way to implement this is to use opaque handles, a standard trick used (for example) for opening files in Linux. An existing MD code can communicate with OpenMM via the handle, but knows none of the details of the handle. It only has to hold on to the handle and give it back to OpenMM.

In the example described below, you will see how this strategy can be implemented for a very simple MD code. Chapter *13* describes the strategies used in integrating OpenMM into real MD codes.

10.4.1 Simple molecular dynamics system

The initial sections of HelloSodiumChloride.cpp represent a very simple molecular dynamics system. The system includes modeling and simulation parameters and the atom and force field data. It also provides a data structure `posInAng[3]` for storing the current state. These sections represent (in highly simplified form) information that would be available from an existing MD code, and will be used to demonstrate how to integrate OpenMM with an existing MD program.

```
// -----------------------------------------------------------------
//                   MODELING AND SIMULATION PARAMETERS
// -----------------------------------------------------------------
static const double Temperature         = 300;     // Kelvins
static const double FrictionInPerPs     = 91.;     // collisions per picosecond
static const double SolventDielectric   = 80.;     // typical for water
static const double SoluteDielectric    = 2.;      // typical for protein

static const double StepSizeInFs        = 2;       // integration step size (fs)
static const double ReportIntervalInFs  = 50;      // how often to issue PDB frame (fs)
static const double SimulationTimeInPs  = 100;     // total simulation time (ps)

// Decide whether to request energy calculations.
static const bool   WantEnergy          = true;
```

```
// -----------------------------------------------------------------
//                   ATOM AND FORCE FIELD DATA
// -----------------------------------------------------------------
// This is not part of OpenMM; just a struct we can use to collect atom
// parameters for this example. Normally atom parameters would come from the
// force field's parameterization file. We're going to use data in Angstrom and
// Kilocalorie units and show how to safely convert to OpenMM's internal unit
// system which uses nanometers and kilojoules.
static struct MyAtomInfo {
    const char* pdb;
    double      mass, charge, vdwRadiusInAng, vdwEnergyInKcal,
                gbsaRadiusInAng, gbsaScaleFactor;
    double      initPosInAng[3];
    double      posInAng[3]; // leave room for runtime state info
} atoms[] = {
// pdb   mass  charge   vdwRad vdwEnergy   gbsaRad gbsaScale  initPos
{" NA ", 22.99,  1,    1.8680, 0.00277,    1.992,   0.8,      8, 0,   0},
{" CL ", 35.45, -1,    2.4700, 0.1000,     1.735,   0.8,     -8, 0,   0},
{" NA ", 22.99,  1,    1.8680, 0.00277,    1.992,   0.8,      0, 9,   0},
{" CL ", 35.45, -1,    2.4700, 0.1000,     1.735,   0.8,      0,-9,   0},
{" NA ", 22.99,  1,    1.8680, 0.00277,    1.992,   0.8,      0, 0,-10},
{" CL ", 35.45, -1,    2.4700, 0.1000,     1.735,   0.8,      0, 0, 10},
{""} // end of list
};
```

10.4.2 Interface routines

The key to our recommended integration strategy is the interface routines. You will need to decide what interface routines are required for effective communication between your existing MD program and OpenMM, but typically there will only be six or seven. In our example, the following four routines suffice:

- **Initialize:** Data structures that already exist in your MD program (i.e., force fields, constraints, atoms in the system) are passed to the `Initialize` routine, which makes appropriate calls to OpenMM and then returns a handle to the OpenMM object that can be used by the existing MD program.
- **Terminate:** Clean up the heap space allocated by `Initialize` by passing the handle to the `Terminate` routine.
- **Advance State:** The `AdvanceState` routine advances the simulation. It requires that the calling function, the existing MD code, gives it a handle.
- **Retrieve State:** When you want to do an analysis or generate some kind of report, you call the `RetrieveState` routine. You have to give it a handle. It then fills in a data structure that is defined in the existing MD code, allowing the MD program to use it in its existing routines without further modification.

Note that these are just descriptions of the routines' functions—you can call them anything you like and implement them in whatever way makes sense for your MD code.

In the example code, the four routines performing these functions, plus an opaque data structure (the handle), would be declared, as shown below. Then, the main program, which sets up, runs, and reports on the simulation, accesses these routines and the opaque data structure (in this case, the variable `omm`). As you can see, it does not have access to any OpenMM declarations, only to the interface routines that you write so there is no need to change the build environment.

```
struct MyOpenMMData;
static MyOpenMMData* myInitializeOpenMM(const MyAtomInfo atoms[],
                                        double temperature,
                                        double frictionInPs,
```

```
                                          double solventDielectric,
                                          double soluteDielectric,
                                          double stepSizeInFs,
                                          std::string& platformName);
static void          myStepWithOpenMM(MyOpenMMData*, int numSteps);
static void          myGetOpenMMState(MyOpenMMData*,
                                        bool wantEnergy,
                                        double& time,
                                        double& energy,
                                        MyAtomInfo atoms[]);
static void          myTerminateOpenMM(MyOpenMMData*);


// -----------------------------------------------------------------
//                              MAIN PROGRAM
// -----------------------------------------------------------------
int main() {
    const int NumReports     = (int)(SimulationTimeInPs*1000 / ReportIntervalInFs + 0.5);
    const int NumSilentSteps = (int)(ReportIntervalInFs / StepSizeInFs + 0.5);

    // ALWAYS enclose all OpenMM calls with a try/catch block to make sure that
    // usage and runtime errors are caught and reported.
    try {
        double        time, energy;
        std::string   platformName;

        // Set up OpenMM data structures; returns OpenMM Platform name.
        MyOpenMMData* omm = myInitializeOpenMM(atoms, Temperature, FrictionInPerPs,
             SolventDielectric, SoluteDielectric, StepSizeInFs, platformName);

        // Run the simulation:
        //   (1) Write the first line of the PDB file and the initial configuration.
        //   (2) Run silently entirely within OpenMM between reporting intervals.
        //   (3) Write a PDB frame when the time comes.
        printf("REMARK  Using OpenMM platform %s\n", platformName.c_str());
        myGetOpenMMState(omm, WantEnergy, time, energy, atoms);
        myWritePDBFrame(1, time, energy, atoms);

        for (int frame=2; frame <= NumReports; ++frame) {
            myStepWithOpenMM(omm, NumSilentSteps);
            myGetOpenMMState(omm, WantEnergy, time, energy, atoms);
            myWritePDBFrame(frame, time, energy, atoms);
        }

        // Clean up OpenMM data structures.
        myTerminateOpenMM(omm);

        return 0; // Normal return from main.
    }

    // Catch and report usage and runtime errors detected by OpenMM and fail.
    catch(const std::exception& e) {
        printf("EXCEPTION: %s\n", e.what());
        return 1;
    }
}
```

We will examine the implementation of each of the four interface routines and the opaque data structure (handle) in

the sections below.

Units

The simple molecular dynamics system described in Section *10.4.1* employs the commonly used units of angstroms and kcals. These differ from the units and parameters used within OpenMM (see Section *18.2*): nanometers and kilojoules. These differences may be small but they are critical and must be carefully accounted for in the interface routines.

Lennard-Jones potential

The Lennard-Jones potential describes the energy between two identical atoms as the distance between them varies.

The van der Waals "size" parameter is used to identify the distance at which the energy between these two atoms is at a minimum (that is, where the van der Waals force is most attractive). There are several ways to specify this parameter, typically, either as the van der Waals radius r_{vdw} or as the actual distance between the two atoms d_{min} (also called r_{min}), which is twice the van der Waals radius r_{vdw}. A third way to describe the potential is through sigma σ, which identifies the distance at which the energy function crosses zero as the atoms move closer together than d_{min}. (See Section *19.6.1* for more details about the relationship between these).

σ turns out to be about 0.89*d_{min}, which is close enough to d_{min} that it makes it hard to distinguish the two. Be very careful that you use the correct value. In the example below, we will show you how to use the built-in OpenMM conversion constants to avoid errors.

Lennard-Jones parameters are defined for pairs of identical atoms, but must also be applied to pairs of dissimilar atoms. That is done by "combining rules" that differ among popular MD codes. Two of the most common are:

- Lorentz-Berthelot (used by AMBER, CHARMM):

$$r = \frac{r_i + r_j}{2}, \epsilon = \sqrt{\epsilon_i \epsilon_j}$$

- Jorgensen (used by OPLS):

$$r = \sqrt{r_i r_j}, \epsilon = \sqrt{\epsilon_i \epsilon_j}$$

where r = the effective van der Waals "size" parameter (minimum radius, minimum distance, or zero crossing (sigma)), and ϵ = the effective van der Waals energy well depth parameter, for the dissimilar pair of atoms i and j.

OpenMM only implements Lorentz-Berthelot directly, but others can be implemented using the CustomNonbondedForce class. (See Section *20.4* for details.)

Opaque handle MyOpenMMData

In this example, the handle used by the interface to OpenMM is a pointer to a struct called `MyOpenMMData`. The pointer itself is opaque, meaning the calling program has no knowledge of what the layout of the object it points to is, or how to use it to directly interface with OpenMM. The calling program will simply pass this opaque handle from one interface routine to another.

There are many different ways to implement the handle. The code below shows just one example. A simulation requires three OpenMM objects (a System, a Context, and an Integrator) and so these must exist within the handle. If other objects were required for a simulation, you would just add them to your handle; there would be no change in the main program using the handle.

```
struct MyOpenMMData {
    MyOpenMMData() : system(0), context(0), integrator(0) {}
    ~MyOpenMMData() {delete system; delete context; delete integrator;}
    OpenMM::System*         system;
    OpenMM::Context*        context;
    OpenMM::Integrator*     integrator;
};
```

In addition to establishing pointers to the required three OpenMM objects, `MyOpenMMData` has a constructor `MyOpenMMData()` that sets the pointers for the three OpenMM objects to zero and a destructor `~MyOpenMMData()` that (in C++) gives the heap space back. This was done in-line in the HelloArgon program, but we recommend you use something like the method here instead.

myInitializeOpenMM

The `myInitializeOpenMM` function takes the data structures and simulation parameters from the existing MD code and returns a new handle that can be used to do efficient computations with OpenMM. It also returns the `platformName` so the calling program knows what platform (e.g., CUDA, OpenCL, Reference) was used.

```
static MyOpenMMData*
myInitializeOpenMM( const MyAtomInfo    atoms[],
                    double              temperature,
                    double              frictionInPs,
                    double              solventDielectric,
                    double              soluteDielectric,
                    double              stepSizeInFs,
                    std::string&        platformName)
```

This initialization routine is very similar to the HelloArgon example program, except that objects are created and put in the handle. For instance, just as in the HelloArgon program, the first step is to load the OpenMM plug-ins, so that the program will run on the best performing platform that is available. Then, a System is created **and** assigned to the handle `omm`. Similarly, forces are added to the System which is already in the handle.

```
// Load all available OpenMM plugins from their default location.
OpenMM::Platform::loadPluginsFromDirectory
       (OpenMM::Platform::getDefaultPluginsDirectory());

// Allocate space to hold OpenMM objects while we're using them.
MyOpenMMData* omm = new MyOpenMMData();

// Create a System and Force objects within the System. Retain a reference
// to each force object so we can fill in the forces. Note: the OpenMM
// System takes ownership of the force objects;don't delete them yourself.
omm->system = new OpenMM::System();
OpenMM::NonbondedForce* nonbond = new OpenMM::NonbondedForce();
OpenMM::GBSAOBCForce*   gbsa    = new OpenMM::GBSAOBCForce();
omm->system->addForce(nonbond);
omm->system->addForce(gbsa);

// Specify dielectrics for GBSA implicit solvation.
gbsa->setSolventDielectric(solventDielectric);
gbsa->setSoluteDielectric(soluteDielectric);
```

In the next step, atoms are added to the System within the handle, with information about each atom coming from the data structure that was passed into the initialization function from the existing MD code. As shown in the HelloArgon program, both the System and the forces need information about the atoms. For those unfamiliar with the C++ Standard

Template Library, the `push_back` function called at the end of this code snippet just adds the given argument to the end of a C++ "vector" container.

```
// Specify the atoms and their properties:
//  (1) System needs to know the masses.
//  (2) NonbondedForce needs charges,van der Waals properties(in MD units!).
//  (3) GBSA needs charge, radius, and scale factor.
//  (4) Collect default positions for initializing the simulation later.
std::vector<Vec3> initialPosInNm;
for (int n=0; *atoms[n].pdb; ++n) {
     const MyAtomInfo& atom = atoms[n];

     omm->system->addParticle(atom.mass);

     nonbond->addParticle(atom.charge,
                         atom.vdwRadiusInAng * OpenMM::NmPerAngstrom
                                             * OpenMM::SigmaPerVdwRadius,
                         atom.vdwEnergyInKcal * OpenMM::KJPerKcal);

     gbsa->addParticle(atom.charge,
                       atom.gbsaRadiusInAng * OpenMM::NmPerAngstrom,
                       atom.gbsaScaleFactor);

     // Convert the initial position to nm and append to the array.
     const Vec3 posInNm(atom.initPosInAng[0] * OpenMM::NmPerAngstrom,
                  atom.initPosInAng[1] * OpenMM::NmPerAngstrom,
                  atom.initPosInAng[2] * OpenMM::NmPerAngstrom);
     initialPosInNm.push_back(posInNm);
```

Units: Here we emphasize the need to pay special attention to the units. As mentioned earlier, the existing MD code in this example uses units of angstroms and kcals, but OpenMM uses nanometers and kilojoules. So the initialization routine will need to convert the values from the existing MD code into the OpenMM units before assigning them to the OpenMM objects.

In the code above, we have used the unit conversion constants that come with OpenMM (e.g., `OpenMM::NmPerAngstrom`) to perform these conversions. Combined with the naming convention of including the units in the variable name (e.g., `initPosInAng`), the unit conversion constants are useful reminders to pay attention to units and minimize errors.

Finally, the initialization routine creates the Integrator and Context for the simulation. Again, note the change in units for the arguments! The routine then gets the platform that will be used to run the simulation and returns that, along with the handle `omm`, back to the calling function.

```
// Choose an Integrator for advancing time, and a Context connecting the
// System with the Integrator for simulation. Let the Context choose the
// best available Platform. Initialize the configuration from the default
// positions we collected above. Initial velocities will be zero but could
// have been set here.
omm->integrator = new OpenMM::LangevinIntegrator(temperature,
frictionInPs,
stepSizeInFs * OpenMM::PsPerFs);
omm->context    = new OpenMM::Context(*omm->system, *omm->integrator);
omm->context->setPositions(initialPosInNm);

platformName = omm->context->getPlatform().getName();
return omm;
```

myGetOpenMMState

The `myGetOpenMMState` function takes the handle and returns the time, energy, and data structure for the atoms in a way that the existing MD code can use them without modification.

```
static void
myGetOpenMMState(MyOpenMMData* omm, bool wantEnergy,
                 double& timeInPs, double& energyInKcal, MyAtomInfo atoms[])
```

Again, this is another interface routine in which you need to be very careful of your units! Note the conversion from the OpenMM units back to the units used in the existing MD code.

```
int infoMask = 0;
infoMask = OpenMM::State::Positions;
if (wantEnergy) {
   infoMask += OpenMM::State::Velocities; // for kinetic energy (cheap)
   infoMask += OpenMM::State::Energy;     // for pot. energy (more expensive)
}
// Forces are also available (and cheap).

const OpenMM::State state = omm->context->getState(infoMask);
timeInPs = state.getTime(); // OpenMM time is in ps already

// Copy OpenMM positions into atoms array and change units from nm to Angstroms.
const std::vector<Vec3>& positionsInNm = state.getPositions();
for (int i=0; i < (int)positionsInNm.size(); ++i)
    for (int j=0; j < 3; ++j)
         atoms[i].posInAng[j] = positionsInNm[i][j] * OpenMM::AngstromsPerNm;

// If energy has been requested, obtain it and convert from kJ to kcal.
energyInKcal = 0;
if (wantEnergy)
   energyInKcal = (state.getPotentialEnergy() + state.getKineticEnergy())
                  * OpenMM::KcalPerKJ;
```

myStepWithOpenMM

The `myStepWithOpenMM` routine takes the handle, uses it to find the Integrator, and then sets the number of steps for the Integrator to take. It does not return any values.

```
static void
myStepWithOpenMM(MyOpenMMData* omm, int numSteps) {
    omm->integrator->step(numSteps);
}
```

myTerminateOpenMM

The `myTerminateOpenMM` routine takes the handle and deletes all the components, e.g., the Context and System, cleaning up the heap space.

```
static void
myTerminateOpenMM(MyOpenMMData* omm) {
    delete omm;
}
```

10.5 HelloEthane Program

The HelloEthane program simulates ethane (H3-C-C-H3) in a vacuum. It is structured similarly to the HelloSodiumChloride example, but includes bonded forces (bond stretch, bond angle bend, dihedral torsion). In setting up these bonded forces, the program illustrates some of the other inconsistencies in definitions and units that you should watch out for.

The bonded forces are added to the system within the initialization interface routine, similar to how the non-bonded forces were added in the HelloSodiumChloride example:

```
// Create a System and Force objects within the System. Retain a reference
// to each force object so we can fill in the forces. Note: the System owns
// the force objects and will take care of deleting them; don't do it yourself!
OpenMM::System&                 system      = *(omm->system = new OpenMM::System());
OpenMM::NonbondedForce&         nonbond     = *new OpenMM::NonbondedForce();
OpenMM::HarmonicBondForce&      bondStretch = *new OpenMM::HarmonicBondForce();
OpenMM::HarmonicAngleForce&     bondBend    = *new OpenMM::HarmonicAngleForce();
OpenMM::PeriodicTorsionForce&   bondTorsion = *new OpenMM::PeriodicTorsionForce();
system.addForce(&nonbond);
system.addForce(&bondStretch);
system.addForce(&bondBend);
system.addForce(&bondTorsion);
```

Constrainable and non-constrainable bonds: In the initialization routine, we also set up the bonds. If constraints are being used, then we tell the System about the constrainable bonds:

```
std::vector< std::pair<int,int> > bondPairs;
for (int i=0; bonds[i].type != EndOfList; ++i) {
    const int*       atom = bonds[i].atoms;
    const BondType& bond = bondType[bonds[i].type];

    if (UseConstraints && bond.canConstrain) {
        system.addConstraint(atom[0], atom[1],
                bond.nominalLengthInAngstroms * OpenMM::NmPerAngstrom);
    }
```

Otherwise, we need to give the HarmonicBondForce the bond stretch parameters.

Warning: The constant used to specify the stiffness may be defined differently between the existing MD code and OpenMM. For instance, AMBER uses the constant, as given in the harmonic *energy* term kx^2, where the force is 2kx (k = constant and x = distance). OpenMM wants the constant, as used in the *force* term kx (with energy $0.5 * kx^2$). So a factor of 2 must be introduced when setting the bond stretch parameters in an OpenMM system using data from an AMBER system.

```
bondStretch.addBond(atom[0], atom[1], bond.nominalLengthInAngstroms * OpenMM::NmPerAngstrom,
                    bond.stiffnessInKcalPerAngstrom2 * 2 * OpenMM::KJPerKcal *
                    OpenMM::AngstromsPerNm * OpenMM::AngstromsPerNm);
```

Non-bond exclusions: Next, we deal with non-bond exclusions. These are used for pairs of atoms that appear close to one another in the network of bonds in a molecule. For atoms that close, normal non-bonded forces do not apply or are reduced in magnitude. First, we create a list of bonds to generate the non- bond exclusions:

```
bondPairs.push_back(std::make_pair(atom[0], atom[1]));
```

OpenMM's non-bonded force provides a convenient routine for creating the common exceptions. These are: (1) for atoms connected by one bond (1-2) or connected by just one additional bond (1-3), Coulomb and van der Waals terms do not apply; and (2) for atoms connected by three bonds (1-4), Coulomb and van der Waals terms apply but are reduced by a force-field dependent scale factor. In general, you may introduce additional exceptions, but the standard ones suffice here and in many other circumstances.

```
// Exclude 1-2, 1-3 bonded atoms from nonbonded forces, and scale down 1-4 bonded atoms.
nonbond.createExceptionsFromBonds(bondPairs, Coulomb14Scale, LennardJones14Scale);

// Create the 1-2-3 bond angle harmonic terms.
for (int i=0; angles[i].type != EndOfList; ++i) {
     const int*       atom  = angles[i].atoms;
     const AngleType& angle = angleType[angles[i].type];

// See note under bond stretch above regarding the factor of 2 here.
bondBend.addAngle(atom[0],atom[1],atom[2],
angle.nominalAngleInDegrees     * OpenMM::RadiansPerDegree,
angle.stiffnessInKcalPerRadian2 * 2 *
OpenMM::KJPerKcal);
}

// Create the 1-2-3-4 bond torsion (dihedral) terms.
for (int i=0; torsions[i].type != EndOfList; ++i) {
     const int*         atom = torsions[i].atoms;
    const TorsionType& torsion = torsionType[torsions[i].type];
    bondTorsion.addTorsion(atom[0],atom[1],atom[2],atom[3],
            torsion.periodicity,
            torsion.phaseInDegrees  * OpenMM::RadiansPerDegree,
            torsion.amplitudeInKcal * OpenMM::KJPerKcal);
}
```

The rest of the code is similar to the HelloSodiumChloride example and will not be covered in detail here. Please refer to the program HelloEthane.cpp itself, which is well-commented, for additional details.

CHAPTER

ELEVEN

PLATFORM-SPECIFIC PROPERTIES

When creating a Context, you can specify values for properties specific to a particular Platform. This is used to control how calculations are done in ways that are outside the scope of the generic OpenMM API.

To do this, pass both the Platform object and a map of property values to the Context constructor:

```
Platform& platform = Platform::getPlatformByName("OpenCL");
map<string, string> properties;
properties["OpenCLDeviceIndex"] = "1";
Context context(system, integrator, platform, properties);
```

After a Context is created, you can use the Platform's `getPropertyValue()` method to query the values of properties.

11.1 OpenCL Platform

The OpenCL Platform recognizes the following Platform-specific properties:

- OpenCLPrecision: This selects what numeric precision to use for calculations. The allowed values are "single", "mixed", and "double". If it is set to "single", nearly all calculations are done in single precision. This is the fastest option but also the least accurate. If it is set to "mixed", forces are computed in single precision but integration is done in double precision. This gives much better energy conservation with only a slight decrease in speed. If it is set to "double", all calculations are done in double precision. This is the most accurate option, but is usually much slower than the others.
- OpenCLUseCpuPme: This selects whether to use the CPU-based PME implementation. The allowed values are "true" or "false". Depending on your hardware, this might (or might not) improve performance. To use this option, you must have FFTW (single precision, multithreaded) installed, and your CPU must support SSE 4.1.
- OpenCLPlatformIndex: When multiple OpenCL implementations are installed on your computer, this is used to select which one to use. The value is the zero-based index of the platform (in the OpenCL sense, not the OpenMM sense) to use, in the order they are returned by the OpenCL platform API. This is useful, for example, in selecting whether to use a GPU or CPU based OpenCL implementation.
- OpenCLDeviceIndex: When multiple OpenCL devices are available on your computer, this is used to select which one to use. The value is the zero-based index of the device to use, in the order they are returned by the OpenCL device API.

The OpenCL Platform also supports parallelizing a simulation across multiple GPUs. To do that, set the OpenCLDeviceIndex property to a comma separated list of values. For example,

```
properties["OpenCLDeviceIndex"] = "0,1";
```

This tells it to use both devices 0 and 1, splitting the work between them.

11.2 CUDA Platform

The CUDA Platform recognizes the following Platform-specific properties:

- CudaPrecision: This selects what numeric precision to use for calculations. The allowed values are "single", "mixed", and "double". If it is set to "single", nearly all calculations are done in single precision. This is the fastest option but also the least accurate. If it is set to "mixed", forces are computed in single precision but integration is done in double precision. This gives much better energy conservation with only a slight decrease in speed. If it is set to "double", all calculations are done in double precision. This is the most accurate option, but is usually much slower than the others.
- CudaUseCpuPme: This selects whether to use the CPU-based PME implementation. The allowed values are "true" or "false". Depending on your hardware, this might (or might not) improve performance. To use this option, you must have FFTW (single precision, multithreaded) installed, and your CPU must support SSE 4.1.
- CudaCompiler: This specifies the path to the CUDA kernel compiler. Versions of CUDA before 7.0 require a separate compiler executable. If you do not specify this, OpenMM will try to locate the compiler itself. Specify this only when you want to override the default location. The logic used to pick the default location depends on the operating system:
  - Mac/Linux: It first looks for an environment variable called OPENMM_CUDA_COMPILER. If that is set, its value is used. Otherwise, the default location is set to /usr/local/cuda/bin/nvcc.
  - Windows: It looks for an environment variable called CUDA_BIN_PATH, then appends nvcc.exe to it. That environment variable is set by the CUDA installer, so it usually is present.
- CudaTempDirectory: This specifies a directory where temporary files can be written while compiling kernels. OpenMM usually can locate your operating system's temp directory automatically (for example, by looking for the TEMP environment variable), so you rarely need to specify this.
- CudaDeviceIndex: When multiple CUDA devices are available on your computer, this is used to select which one to use. The value is the zero-based index of the device to use, in the order they are returned by the CUDA API.
- CudaUseBlockingSync: This is used to control how the CUDA runtime synchronizes between the CPU and GPU. If this is set to "true" (the default), CUDA will allow the calling thread to sleep while the GPU is performing a computation, allowing the CPU to do other work. If it is set to "false", CUDA will spin-lock while the GPU is working. Setting it to "false" can improve performance slightly, but also prevents the CPU from doing anything else while the GPU is working.

The CUDA Platform also supports parallelizing a simulation across multiple GPUs. To do that, set the CudaDeviceIndex property to a comma separated list of values. For example,

```
properties["CudaDeviceIndex"] = "0,1";
```

This tells it to use both devices 0 and 1, splitting the work between them.

11.3 CPU Platform

The CPU Platform recognizes the following Platform-specific properties:

- CpuThreads: This specifies the number of CPU threads to use. If you do not specify this, OpenMM will select a default number of threads as follows:
  - If an environment variable called OPENMM_CPU_THREADS is set, its value is used as the number of threads.

- Otherwise, the number of threads is set to the number of logical CPU cores in the computer it is running on.

Usually the default value works well. This is mainly useful when you are running something else on the computer at the same time, and you want to prevent OpenMM from monopolizing all available cores.

11.4 Determinism

Whether a simulation is deterministic will depend on what plaform you run on in addition to what settings/methods you use. For instance, as of this writing, using PME on the Reference, OpenCL, and double-precision CUDA will result in deterministic simulations. Single-precision CUDA and CPU platforms are not deterministic in this case. However, none of this behavior is guaranteed in future versions. In many cases it will still result in an identical trajectory. If determinism is a critical for your needs, you should carefully check to ensure that your settings and platform allow for this.

CHAPTER

TWELVE

USING OPENMM WITH SOFTWARE WRITTEN IN LANGUAGES OTHER THAN C++

Although the native OpenMM API is object-oriented C++ code, it is possible to directly translate the interface so that it is callable from C, Fortran 95, and Python with no substantial conceptual changes. We have developed a straightforward mapping for these languages that, while perhaps not the most elegant possible, has several advantages:

- Almost all documentation, training, forum discussions, and so on are equally useful to users of all these languages. There are syntactic differences of course, but all the important concepts remain unchanged.
- We are able to generate the C, Fortran, and Python APIs from the C++ API. Obviously, this reduces development effort, but more importantly it means that the APIs are likely to be error-free and are always available immediately when the native API is updated.
- Because OpenMM performs expensive operations "in bulk" there is no noticeable overhead in accessing these operations through the C, Fortran, or Python APIs.
- All symbols introduced to a C or Fortran program begin with the prefix "`OpenMM_`" so will not interfere with symbols already in use.

Availability of APIs in other languages: All necessary C and Fortran bindings are built in to the main OpenMM library; no separate library is required. The Python wrappers are contained in a module that is distributed with OpenMM and that can be installed by executing its setup.py script in the standard way.

(This doesn't apply to most users: if you are building your own OpenMM from source using CMake and want the API bindings generated, be sure to enable the `OPENMM_BUILD_C_AND_FORTRAN_WRAPPERS` option for C and Fortran, or `OPENMM_BUILD_PYTHON_WRAPPERS` option for Python. The Python module will be placed in a subdirectory of your main build directory called "python")

Documentation for APIs in other languages: While there is extensive Doxygen documentation available for the C++ and Python APIs, there is no separate on-line documentation for the C and Fortran API. Instead, you should use the C++ documentation, employing the mappings described here to figure out the equivalent syntax in C or Fortran.

12.1 C API

Before you start writing your own C program that calls OpenMM, be sure you can build and run the two C examples that are supplied with OpenMM (see Chapter *10*). These can be built from the supplied `Makefile` on Linux and Mac, or supplied `NMakefile` and Visual Studio solution files on Windows.

The example programs are `HelloArgonInC` and `HelloSodiumChlorideInC`. The argon example serves as a quick check that your installation is set up properly and you know how to build a C program that is linked with OpenMM. It will also tell you whether OpenMM is executing on the GPU or is running (slowly) on the Reference platform. However, the argon example is not a good template to follow for your own programs. The sodium chloride example, though necessarily simplified, is structured roughly in the way we recommended you set up your own programs to call OpenMM. Please be sure you have both of these programs executing successfully on your machine before continuing.

12.1.1 Mechanics of using the C API

The C API is generated automatically from the C++ API when OpenMM is built. There are two resulting components: C bindings (functions to call), and C declarations (in a header file). The C bindings are small `extern` (global) interface functions, one for every method of every OpenMM class, whose signatures (name and arguments) are predictable from the class name and method signatures. There are also "helper" types and functions provided for the few cases in which the C++ behavior cannot be directly mapped into C. These interface and helper functions are compiled in to the main OpenMM library so there is nothing special you have to do to get access to them.

In the `include` subdirectory of your OpenMM installation directory, there is a machine-generated header file `OpenMMCWrapper.h` that should be #included in any C program that is to make calls to OpenMM functions. That header contains declarations for all the OpenMM C interface functions and related types. Note that if you follow our suggested structure, you will not need to include this file in your `main()` compilation unit but can instead use it only in a local file that you write to provide a simple interface to your existing code (see Chapter *10*).

12.1.2 Mapping from the C++ API to the C API

The automated generator of the C "wrappers" follows the translation strategy shown in Table 12-1. The idea is that if you see the construct on the left in the C++ API documentation, you should interpret it as the corresponding construct on the right in C. Please look at the supplied example programs to see how this is done in practice.

Construct	C++ API declaration	Equivalent in C API
namespace	OpenMM::	OpenMM_ (prefix)
class	class OpenMM::ClassName	typedef OpenMM_ClassName
constant	OpenMM::RadiansPerDeg	OpenMM_RadiansPerDeg (static constant)
class enum	OpenMM::State::Positions	OpenMM_State_Positions
constructor	new OpenMM::ClassName()	OpenMM_ClassName* OpenMM_ClassName_create()<br>(additional constructors are _create_2(), etc.)
destructor	OpenMM::ClassName* thing;<br>delete thing;	OpenMM_ClassName* thing;<br>OpenMM_ClassName_destroy(thing);
class method	OpenMM::ClassName* thing;<br>thing->method(args);	OpenMM_ClassName* thing;<br>OpenMM_ClassName_method(thing, args)
Boolean (type & constants)	bool<br>true, false	OpenMM_Boolean<br>OpenMM_True(1), OpenMM_False(0)
string	std::string	char*
3-vector	OpenMM::Vec3	typedef OpenMM_Vec3
arrays	std::vector<std::string><br>std::vector<double><br>std::vector<Vec3><br>std::vector<std::pair<int,int>><br>std::map<std::string,double>	typedef OpenMM_StringArray<br>typedef OpenMM_DoubleArray<br>typedef OpenMM_Vec3Array<br>typedef OpenMM_BondArray<br>typedef OpenMM_ParameterArray

Table 12-1: Default mapping of objects from the C++ API to the C API There are some exceptions to the generic translation rules shown in the table; they are enumerated in the next section. And because there are no C++ API equivalents to the array types, they are described in detail below.

12.1.3 Exceptions

These two methods are handled somewhat differently in the C API than in the C++ API:

- **OpenMM::Context::getState()** The C version, `OpenMM_Context_getState()`, returns a pointer to a heap allocated `OpenMM_State` object. You must then explicitly destroy this `State` object when you are done with it, by calling `OpenMM_State_destroy()`.

- **OpenMM::Platform::loadPluginsFromDirectory()** The C version `OpenMM_Platform_loadPluginsFromDirectory()` returns a heap-allocated `OpenMM_StringArray` object containing a list of all the file names that were successfully loaded. You must then explicitly destroy this `StringArray` object when you are done with it. Do not ignore the return value; if you do you'll have a memory leak since the `StringArray` will still be allocated.

(In the C++ API, the equivalent methods return references into existing memory rather than new heap-allocated memory, so the returned objects do not need to be destroyed.)

12.1.4 OpenMM_Vec3 helper type

Unlike the other OpenMM objects which are opaque and manipulated via pointers, the C API provides an explicit definition for the C `OpenMM_Vec3` type that is compatible with the `OpenMM::Vec3` type. The definition of `OpenMM_Vec3` is:

```
typedef struct {double x, y, z;} OpenMM_Vec3;
```

You can work directly with the individual fields of this type from your C program if you want. For convenience, a scale() function is provided that creates a new OpenMM_Vec3 from an old one and a scale factor:

```
OpenMM_Vec3 OpenMM_Vec3_scale(const OpenMM_Vec3 vec, double scale);
```

12.1.5 Array helper types

C++ has built-in container types `std::vector` and `std::map` which OpenMM uses to manipulate arrays of objects. These don't have direct equivalents in C, so we supply special array types for each kind of object for which OpenMM creates containers. These are: string, double, Vec3, bond, and parameter map. See Table 12-2 for the names of the C types for each of these object arrays. Each of the array types provides these functions (prefixed by `OpenMM_` and the actual *Thing* name), with the syntax shown conceptually since it differs slightly for each kind of object.

Function	Operation
*Thing*Array* create(int size)	Create a heap-allocated array of *Things*, with space pre-allocated to hold `size` of them. You can start at `size==0` if you want since these arrays are dynamically resizeable.
void destroy(*Thing*Array*)	Free the heap space that is currently in use for the passed-in array of *Things*.
int getSize(*Thing*Array*)	Return the current number of *Things* in this array. This means you can `get()` and `set()` elements up to `getSize()-1`.
void resize(*Thing*Array*, int size)	Change the size of this array to the indicated value which may be smaller or larger than the current size. Existing elements remain in their same locations as long as they still fit.
void append(*Thing*Array*, *Thing*)	Add a *Thing* to the end of the array, increasing the array size by one. The precise syntax depends on the actual type of *Thing*; see below.
void set(*Thing*Array*, int index, *Thing*)	Store a copy of *Thing* in the indicated element of the array (indexed from 0). The array must be of length at least `index+1`; you can't grow the array with this function.
Thing get(*Thing*Array*, int index)	Retrieve a particular element from the array (indexed from 0). (For some Things the value is returned in arguments rather than as the function return.)

Table 12-2: Generic description of array helper types

Here are the exact declarations with deviations from the generic description noted, for each of the array types.

OpenMM_DoubleArray

```
OpenMM_DoubleArray*
              OpenMM_DoubleArray_create(int size);
void          OpenMM_DoubleArray_destroy(OpenMM_DoubleArray*);
int           OpenMM_DoubleArray_getSize(const OpenMM_DoubleArray*);
void          OpenMM_DoubleArray_resize(OpenMM_DoubleArray*, int size);
void          OpenMM_DoubleArray_append(OpenMM_DoubleArray*, double value);
void          OpenMM_DoubleArray_set(OpenMM_DoubleArray*, int index, double value);
double        OpenMM_DoubleArray_get(const OpenMM_DoubleArray*, int index);
```

OpenMM_StringArray

```
OpenMM_StringArray*
              OpenMM_StringArray_create(int size);
void          OpenMM_StringArray_destroy(OpenMM_StringArray*);
int           OpenMM_StringArray_getSize(const OpenMM_StringArray*);
void          OpenMM_StringArray_resize(OpenMM_StringArray*, int size);
void          OpenMM_StringArray_append(OpenMM_StringArray*, const char* string);
void          OpenMM_StringArray_set(OpenMM_StringArray*, int index, const char* string);
const char*   OpenMM_StringArray_get(const OpenMM_StringArray*, int index);
```

OpenMM_Vec3Array

```
OpenMM_Vec3Array*
              OpenMM_Vec3Array_create(int size);
void          OpenMM_Vec3Array_destroy(OpenMM_Vec3Array*);
int           OpenMM_Vec3Array_getSize(const OpenMM_Vec3Array*);
void          OpenMM_Vec3Array_resize(OpenMM_Vec3Array*, int size);
void          OpenMM_Vec3Array_append(OpenMM_Vec3Array*, const OpenMM_Vec3 vec);
void          OpenMM_Vec3Array_set(OpenMM_Vec3Array*, int index, const OpenMM_Vec3 vec);
const OpenMM_Vec3*
              OpenMM_Vec3Array_get(const OpenMM_Vec3Array*, int index);
```

OpenMM_BondArray

Note that bonds are specified by pairs of integers (the atom indices). The `get()` method returns those in a pair of final arguments rather than as its functional return.

```
OpenMM_BondArray*
              OpenMM_BondArray_create(int size);
void          OpenMM_BondArray_destroy(OpenMM_BondArray*);
int           OpenMM_BondArray_getSize(const OpenMM_BondArray*);
void          OpenMM_BondArray_resize(OpenMM_BondArray*, int size);
void          OpenMM_BondArray_append(OpenMM_BondArray*, int particle1, int particle2);
void          OpenMM_BondArray_set(OpenMM_BondArray*, int index, int particle1, int particle2);
void          OpenMM_BondArray_get(const OpenMM_BondArray*, int index,
                                   int* particle1, int* particle2);
```

OpenMM_ParameterArray

OpenMM returns references to internal `ParameterArrays` but does not support user-created `ParameterArrays`, so only the `get()` and `getSize()` functions are available. Also, note that since this is actually a map rather than an

array, the "index" is the *name* of the parameter rather than its ordinal.

```
int          OpenMM_ParameterArray_getSize(const OpenMM_ParameterArray*);
double       OpenMM_ParameterArray_get(const OpenMM_ParameterArray*, const char* name);
```

12.2 Fortran 95 API

Before you start writing your own Fortran program that calls OpenMM, be sure you can build and run the two Fortran examples that are supplied with OpenMM (see Chapter *10*). These can be built from the supplied `Makefile` on Linux and Mac, or supplied `NMakefile` and Visual Studio solution files on Windows.

The example programs are `HelloArgonInFortran` and `HelloSodiumChlorideInFortran`. The argon example serves as a quick check that your installation is set up properly and you know how to build a Fortran program that is linked with OpenMM. It will also tell you whether OpenMM is executing on the GPU or is running (slowly) on the Reference platform. However, the argon example is not a good template to follow for your own programs. The sodium chloride example, though necessarily simplified, is structured roughly in the way we recommended you set up your own programs to call OpenMM. Please be sure you have both of these programs executing successfully on your machine before continuing.

12.2.1 Mechanics of using the Fortran API

The Fortran API is generated automatically from the C++ API when OpenMM is built. There are two resulting components: Fortran bindings (subroutines to call), and Fortran declarations of types and subroutines (in the form of a Fortran 95 module file). The Fortran bindings are small interface subroutines, one for every method of every OpenMM class, whose signatures (name and arguments) are predictable from the class name and method signatures. There are also "helper" types and subroutines provided for the few cases in which the C++ behavior cannot be directly mapped into Fortran. These interface and helper subroutines are compiled in to the main OpenMM library so there is nothing special you have to do to get access to them.

Because Fortran is case-insensitive, calls to Fortran subroutines (however capitalized) are mapped by the compiler into all-lowercase or all-uppercase names, and different compilers use different conventions. The automatically-generated OpenMM Fortran "wrapper" subroutines, which are generated in C and thus case-sensitive, are provided in two forms for compatibility with the majority of Fortran compilers, including Intel Fortran and gfortran. The two forms are: (1) all-lowercase with a trailing underscore, and (2) all-uppercase without a trailing underscore. So regardless of the Fortran compiler you are using, it should find a suitable subroutine to call in the main OpenMM library.

In the `include` subdirectory of your OpenMM installation directory, there is a machine-generated module file `OpenMMFortranModule.f90` that must be compiled along with any Fortran program that is to make calls to OpenMM functions. (You can look at the `Makefile` or Visual Studio solution file provided with the OpenMM examples to see how to build a program that uses this module file.) This module file contains definitions for two modules: `MODULE OpenMM_Types` and `MODULE OpenMM`; however, only the `OpenMM` module will appear in user programs (it references the other module internally). The modules contain declarations for all the OpenMM Fortran interface subroutines, related types, and parameters (constants). Note that if you follow our suggested structure, you will not need to `use` the `OpenMM` module in your `main()` compilation unit but can instead use it only in a local file that you write to provide a simple interface to your existing code (see Chapter *10*).

12.2.2 Mapping from the C++ API to the Fortran API

The automated generator of the Fortran "wrappers" follows the translation strategy shown in Table 12-3. The idea is that if you see the construct on the left in the C++ API documentation, you should interpret it as the corresponding construct on the right in Fortran. Please look at the supplied example programs to see how this is done in practice.

Note that all subroutines and modules are declared with "`implicit none`", meaning that the type of every symbol is declared explicitly and should not be inferred from the first letter of the symbol name.

Construct	C++ API declaration	Equivalent in Fortran API
namespace	OpenMM::	OpenMM_ (prefix)
class	class OpenMM::ClassName	type (OpenMM_ClassName)
constant	OpenMM::RadiansPerDeg	parameter (OpenMM_RadiansPerDeg)
class enum	OpenMM::State::Positions	parameter (OpenMM_State_Positions)
constructor	new OpenMM::ClassName()	type (OpenMM_ClassName) thing<br>call OpenMM_ClassName_create(thing)<br>(additional constructors are _create_2(), etc.)
destructor	OpenMM::ClassName* thing;<br>delete thing;	type (OpenMM_ClassName) thing<br>call OpenMM_ClassName_destroy(thing)
class method	OpenMM::ClassName* thing;<br>thing->method(args*)	type (OpenMM_ClassName) thing<br>call OpenMM_ClassName_method(thing, args)
Boolean (type & constants)	bool<br>true<br>false	integer*4<br>parameter (OpenMM_True=1)<br>parameter (OpenMM_False=0)
string	std::string	character(*)
3-vector	OpenMM::Vec3	real*8 vec(3)
arrays	std::vector<std::string><br>std::vector<double><br>std::vector<Vec3><br>std::vector<std::pair<int,int>><br>std::map<std::string, double>	type (OpenMM_StringArray)<br>type (OpenMM_DoubleArray)<br>type (OpenMM_Vec3Array)<br>type (OpenMM_BondArray)<br>type (OpenMM_ParameterArray)

Table 12-3: Default mapping of objects from the C++ API to the Fortran API

Because there are no C++ API equivalents to the array types, they are described in detail below.

12.2.3 OpenMM_Vec3 helper type

Unlike the other OpenMM objects which are opaque and manipulated via pointers, the Fortran API uses an ordinary `real*8(3)` array in place of the `OpenMM::Vec3` type. You can work directly with the individual elements of this type from your Fortran program if you want. For convenience, a `scale()` function is provided that creates a new Vec3 from an old one and a scale factor:

```
subroutine OpenMM_Vec3_scale(vec, scale, result)
real*8 vec(3), scale, result(3)
```

No explicit `type(OpenMM_Vec3)` is provided in the Fortran API since it is not needed.

12.2.4 Array helper types

C++ has built-in container types `std::vector` and `std::map` which OpenMM uses to manipulate arrays of objects. These don't have direct equivalents in Fortran, so we supply special array types for each kind of object for which OpenMM creates containers. These are: string, double, Vec3, bond, and parameter map. See Table 12-4 for the names of the Fortran types for each of these object arrays. Each of the array types provides these functions (prefixed by `OpenMM_` and the actual *Thing* name), with the syntax shown conceptually since it differs slightly for each kind of object.

Function	Operation
subroutine create(array,size)<br>type (OpenMM_*Thing*Array) array<br>integer*4 size	Create a heap-allocated array of *Things*, with space pre-allocated to hold `size` of them. You can start at `size`==0 if you want since these arrays are dynamically resizeable.
subroutine destroy(array)<br>type (OpenMM_*Thing*Array) array	Free the heap space that is currently in use for the passed-in array of *Things*.
function getSize(array)<br>type (OpenMM_*Thing*Array) array<br>integer*4 size	Return the current number of *Things* in this array. This means you can `get()` and `set()` elements up to `getSize()`.
subroutine resize(array,size)<br>type (OpenMM_*Thing*Array) array<br>integer*4 size	Change the size of this array to the indicated value which may be smaller or larger than the current size. Existing elements remain in their same locations as long as they still fit.
subroutine append(array,elt)<br>type (OpenMM_*Thing*Array) array<br>*Thing* elt	Add a *Thing* to the end of the array, increasing the array size by one. The precise syntax depends on the actual type of *Thing*; see below.
subroutine set(array,index,elt)<br>type (OpenMM_*Thing*Array) array<br>integer*4 size<br>*Thing* elt	Store a copy of `elt` in the indicated element of the array (indexed from 1). The array must be of length at least `index`; you can't grow the array with this function.
subroutine get(array,index,elt)<br>type (OpenMM_*Thing*Array) array<br>integer*4 size<br>*Thing* elt	Retrieve a particular element from the array (indexed from 1). Some *Things* require more than one argument to return.

Table 12-4: Generic description of array helper types

Here are the exact declarations with deviations from the generic description noted, for each of the array types.

OpenMM_DoubleArray

```
subroutine OpenMM_DoubleArray_create(array, size)
    integer*4 size
    type (OpenMM_DoubleArray) array
subroutine OpenMM_DoubleArray_destroy(array)
```

```
    type (OpenMM_DoubleArray) array
function OpenMM_DoubleArray_getSize(array)
    type (OpenMM_DoubleArray) array
    integer*4 OpenMM_DoubleArray_getSize
subroutine OpenMM_DoubleArray_resize(array, size)
    type (OpenMM_DoubleArray) array
    integer*4 size
subroutine OpenMM_DoubleArray_append(array, value)
    type (OpenMM_DoubleArray) array
    real*8 value
subroutine OpenMM_DoubleArray_set(array, index, value)
    type (OpenMM_DoubleArray) array
    integer*4 index
    real*8 value
subroutine OpenMM_DoubleArray_get(array, index, value)
    type (OpenMM_DoubleArray) array
    integer*4 index
    real*8 value
```

OpenMM_StringArray

```
subroutine OpenMM_StringArray_create(array, size)
    integer*4 size
    type (OpenMM_StringArray) array
subroutine OpenMM_StringArray_destroy(array)
    type (OpenMM_StringArray) array
function OpenMM_StringArray_getSize(array)
    type (OpenMM_StringArray) array
    integer*4 OpenMM_StringArray_getSize
subroutine OpenMM_StringArray_resize(array, size)
    type (OpenMM_StringArray) array
    integer*4 size
subroutine OpenMM_StringArray_append(array, str)
    type (OpenMM_StringArray) array
    character(*) str
subroutine OpenMM_StringArray_set(array, index, str)
    type (OpenMM_StringArray) array
    integer*4 index
    character(*) str
subroutine OpenMM_StringArray_get(array, index, str)
    type (OpenMM_StringArray) array
    integer*4 index
    character(*)str
```

OpenMM_Vec3Array

```
subroutine OpenMM_Vec3Array_create(array, size)
    integer*4 size
    type (OpenMM_Vec3Array) array
subroutine OpenMM_Vec3Array_destroy(array)
    type (OpenMM_Vec3Array) array
function OpenMM_Vec3Array_getSize(array)
    type (OpenMM_Vec3Array) array
    integer*4 OpenMM_Vec3Array_getSize
subroutine OpenMM_Vec3Array_resize(array, size)
```

```
    type (OpenMM_Vec3Array) array
    integer*4 size
subroutine OpenMM_Vec3Array_append(array, vec)
    type (OpenMM_Vec3Array) array
    real*8 vec(3)
subroutine OpenMM_Vec3Array_set(array, index, vec)
    type (OpenMM_Vec3Array) array
    integer*4 index
    real*8 vec(3)
subroutine OpenMM_Vec3Array_get(array, index, vec)
    type (OpenMM_Vec3Array) array
    integer*4 index
    real*8 vec (3)
```

OpenMM_BondArray

Note that bonds are specified by pairs of integers (the atom indices). The `get()` method returns those in a pair of final arguments rather than as its functional return.

```
subroutine OpenMM_BondArray_create(array, size)
    integer*4 size
    type (OpenMM_BondArray) array
subroutine OpenMM_BondArray_destroy(array)
    type (OpenMM_BondArray) array
function OpenMM_BondArray_getSize(array)
    type (OpenMM_BondArray) array
    integer*4 OpenMM_BondArray_getSize
subroutine OpenMM_BondArray_resize(array, size)
    type (OpenMM_BondArray) array
    integer*4 size
subroutine OpenMM_BondArray_append(array, particle1, particle2)
    type (OpenMM_BondArray) array
    integer*4 particle1, particle2
subroutine OpenMM_BondArray_set(array, index, particle1, particle2)
    type (OpenMM_BondArray) array
    integer*4 index, particle1, particle2
subroutine OpenMM_BondArray_get(array, index, particle1, particle2)
    type (OpenMM_BondArray) array
    integer*4 index, particle1, particle2
```

OpenMM_ParameterArray

OpenMM returns references to internal `ParameterArrays` but does not support user-created `ParameterArrays`, so only the `get()` and `getSize()` functions are available. Also, note that since this is actually a map rather than an array, the "index" is the *name* of the parameter rather than its ordinal.

```
function OpenMM_ParameterArray_getSize(array)
    type (OpenMM_ParameterArray) array
    integer*4 OpenMM_ParameterArray_getSize
subroutine OpenMM_ParameterArray_get(array, name, param)
    type (OpenMM_ParameterArray) array
    character(*) name
    character(*) param
```

12.3 Python API

12.3.1 Installing the Python API

There are currently two types of packages for installing the Python API. One contains wrapper source code for Unix-type machines (including Linux and Mac operating systems). You will need a C++ compiler to install it using this type of package. The other type of installation package is a binary package which contains compiled wrapper code for Windows machines (no compilers are needed to install binary packages).

Installing on Windows

OpenMM on Windows only works with Python 3.3, so make sure that version is installed before you try installing. For Python installation packages and instructions, go to http://python.org. Note that if you have a 64-bit machine, you should still install the 32-bit version of Python since the OpenMM Python API binary is 32-bit. We suggest that you install Python using the default options.

Double click on the Python API Installer icon, located in the top level directory for the OpenMM installation (by default, this is C:Program FilesOpenMM). This will install the OpenMM package into the Python installation area. If you have more than one Python installation, you will be asked which Python to use—make sure to select Python 3.3.

Installing on Linux and Mac

Make sure you have Python 2.6 or later installed. For Python installation packages and instructions, go to http://python.org. If you do not have the correct Python version, install a valid version using the default options. Most versions of Linux and Mac OS X have a suitable Python preinstalled. You can check by typing "`python --version`" in a terminal window.

You must have a C++ compiler to install the OpenMM Python API. If you are using a Mac, install Apple's Xcode development tools (http://developer.apple.com/TOOLS/Xcode) to get the needed compiler. On other Unix-type systems, install gcc or clang.

The install.sh script installs the Python API automatically as part of the installation process, so you probably already have it installed. If for some reason you need to install it manually, you can do that with the `setup.py` script included with OpenMM. Before executing this script, you must set two environment variables: `OPENMM_INCLUDE_PATH` must point to the directory containing OpenMM header files, and `OPENMM_LIB_PATH` must point to the directory containing OpenMM library files. Assuming OpenMM is installed in the default location (`/usr/local/openmm`), you would type the following commands. Note that if you are using the system Python (as opposed to a locally installed version), you may need to use the `sudo` command when running `python setup.py install`.

```
export OPENMM_INCLUDE_PATH=/usr/local/openmm/include
export OPENMM_LIB_PATH=/usr/local/openmm/lib
python setup.py build
python setup.py install
```

If you are compiling OpenMM from source, you can also install by building the "PythonInstall" target:

`make PythonInstall` OR `sudo make PythonInstall`

12.3.2 Mapping from the C++ API to the Python API

The Python API follows the C++ API as closely as possible. There are three notable differences:

1. The `getState()` method in the `Context` class takes Pythonic-type arguments to indicate which state variables should be made available. For example:

```
myContext.getState(getEnergy=True, getForce=False, ...)
```

2. Wherever the C++ API uses references to return multiple values from a method, the Python API returns a tuple. For example, in C++ you would query a HarmonicBondForce for a bond's parameters as follows:

```
int particle1, particle2;
double length, k;
f.getBondParameters(i, particle1, particle2, length, k);
```

In Python, the equivalent code is:

```
[particle1, particle2, length, k] = f.getBondParameters(i)
```

3. Unlike C++, the Python API accepts and returns quantities with units attached to most values (see Section *12.3.4* below for details). In short, this means that while values in C++ have *implicit* units, the Python API returns objects that have values and *explicit* units.

12.3.3 Mechanics of using the Python API

When using the Python API, be sure to include the GPU support libraries in your library path, just as you would for a C++ application. This is set with the `LD_LIBRARY_PATH` environment variable on Linux, `DYLD_LIBRARY_PATH` on Mac, or `PATH` on Windows. See Chapter *3* for details.

The Python API is contained in the simtk.openmm package, while the units code is contained in the simtk.units package. (The application layer, described in the Application Guide, is contained in the simtk.openmm.app package.) A program using it will therefore typically begin

```
import simtk.openmm as mm
import simtk.unit as unit
```

Creating and using OpenMM objects is then done exactly as in C++:

```
system = mm.System()
nb = mm.NonbondedForce()
nb.setNonbondedMethod(mm.NonbondedForce.CutoffNonPeriodic)
nb.setCutoffDistance(1.2*unit.nanometer)
system.addForce(nb)
```

Note that when setting the cutoff distance, we explicitly specify that it is in nanometers. We could just as easily specify it in different units:

```
nb.setCutoffDistance(12*unit.angstrom)
```

The use of units in OpenMM is discussed in the next section.

12.3.4 Units and dimensional analysis

Why does the Python API include units?

The C++ API for OpenMM uses an *implicit* set of units for physical quantities such as lengths, masses, energies, etc. These units are based on daltons, nanometers, and picoseconds for the mass, length, and time dimensions, respectively. When using the C++ API, it is very important to ensure that quantities being manipulated are always expressed in terms of these units. For example, if you read in a distance in Angstroms, you must multiply that distance by a conversion factor to turn it into nanometers before using it in the C++ API. Such conversions can be a source of tedium and errors. This is true in many areas of scientific programming. Units confusion was blamed for the loss of the Mars Climate

Orbiter spacecraft in 1999, at a cost of more than $100 million. Units were introduced in the Python API to minimize the chance of such errors.

The Python API addresses the potential problem of conversion errors by using quantities with explicit units. If a particular distance is expressed in Angstroms, the Python API will know that it is in Angstroms. When the time comes to call the C++ API, it will understand that the quantity must be converted to nanometers. You, the programmer, must declare upfront that the quantity is in Angstrom units, and the API will take care of the details from then on. Using explicit units is a bit like brushing your teeth: it requires some effort upfront, but it probably saves you trouble in the long run.

Quantities, units, and dimensions

The explicit unit system is based on three concepts: Dimensions, Units, and Quantities.

Dimensions are measurable physical concepts such as mass, length, time, and energy. Energy is actually a composite dimension based on mass, length, and time.

A Unit defines a linear scale used to measure amounts of a particular physical Dimension. Examples of units include meters, seconds, joules, inches, and grams.

A Quantity is a specific amount of a physical Dimension. An example of a quantity is "0.63 kilograms". A Quantity is expressed as a combination of a value (e.g., 0.63), and a Unit (e.g., kilogram). The same Quantity can be expressed in different Units.

The set of BaseDimensions defined in the simtk.unit module includes:

- mass
- length
- time
- temperature
- amount
- charge
- luminous intensity

These are not precisely the same list of base dimensions used in the SI unit system. SI defines "current" (charge per time) as a base unit, while simtk.unit uses "charge". And simtk.unit treats angle as a dimension, even though angle quantities are often considered dimensionless. In this case, we choose to err on the side of explicitness, particularly because interconversion of degrees and radians is a frequent source of unit headaches.

Units examples

Many common units are defined in the simtk.unit module.

```
from simtk.unit import nanometer, angstrom, dalton
```

Sometimes you don't want to type the full unit name every time, so you can assign it a shorter name using the `as` functionality:

```
from simtk.unit import nanometer as nm
```

New quantities can be created from a value and a unit. You can use either the multiply operator ('*') or the explicit Quantity constructor:

```
from simk.unit import nanometer, Quantity
# construct a Quantity using the multiply operator
bond_length = 1.53 * nanometer
# equivalently using the explicit Quantity constructor
bond_length = Quantity(1.53, nanometer)
# or more verbosely
bond_length = Quantity(value=1.53, unit=nanometer)
```

Arithmetic with units

Addition and subtraction of quantities is only permitted between quantities that share the same dimension. It makes no sense to add a mass to a distance. If you attempt to add or subtract two quantities with different dimensions, an exception will be raised. This is a good thing; it helps you avoid errors.

```
x = 5.0*dalton + 4.3*nanometer; # error
```

Addition or subtraction of quantities with the same dimension, but different units, is fine, and results in a new quantity created using the correct conversion factor between the units used.

```
x = 1.3*nanometer + 5.6*angstrom; # OK, result in nanometers
```

Quantities can be added and subtracted. Naked Units cannot.

Multiplying or dividing two quantities creates a new quantity with a composite dimension. For example, dividing a distance by a time results in a velocity.

```
from simtk.unit import kilogram, meter, second
a = 9.8 * meter / second**2; # acceleration
m = 0.36 * kilogram; # mass
F = m * a; # force in kg*m/s**2::
```

Multiplication or division of two Units results in a composite Unit.

```
mps = meter / second
```

Unlike amount (moles), angle (radians) is arguably dimensionless. But simtk.unit treats angle as another dimension. Use the trigonometric functions from the simtk.unit module (not those from the Python math module!) when dealing with Units and Quantities.

```
from simtk.unit import sin, cos, acos
x = sin(90.0*degrees)
angle = acos(0.68); # returns an angle quantity (in radians)
```

The method `pow()` is a built-in Python method that works with Quantities and Units.

```
area = pow(3.0*meter, 2)
# or, equivalently
area = (3.0*meter)**2
# or
area = 9.0*(meter**2)
```

The method `sqrt()` is not as built-in as `pow()`. Do not use the Python `math.sqrt()` method with Units and Quantities. Use the `simtk.unit.sqrt()` method instead:

```
from simtk.unit import sqrt
side_length = sqrt(4.0*meter**2)
```

Atomic scale mass and energy units are "per amount"

Mass and energy units at the atomic scale are specified "per amount" in the simtk.unit module. Amount (mole) is one of the seven fundamental dimensions in the SI unit system. The atomic scale mass unit, dalton, is defined as grams per mole. The dimension of dalton is therefore mass/amount, instead of simply mass. Similarly, the atomic scale energy unit, kilojoule_per_mole (and kilocalorie_per_mole) has "per amount" in its dimension. Be careful to always use "per amount" mass and energy types at the atomic scale, and your dimensional analysis should work out properly.

The energy unit kilocalories_per_mole does not have the same Dimension as the macroscopic energy unit kilocalories. Molecular scientists sometimes use the word "kilocalories" when they mean "kilocalories per mole". Use "kilocalories per mole" or"kilojoules per mole" for molecular energies. Use "kilocalories" for the metabolic energy content of your lunch. The energy unit kilojoule_per_mole happens to go naturally with the units nanometer, picoseconds, and dalton. This is because 1 kilojoule/mole happens to be equal to 1 gram-nanometer^2/mole-picosecond^2, and is therefore consistent with the molecular dynamics unit system used in the C++ OpenMM API.

These "per mole" units are what you should be using for molecular calculations, as long as you are using SI / cgs / calorie sorts of units.

SI prefixes

Many units with SI prefixes such as "milligram" (milli) and "kilometer" (kilo) are provided in the simtk.unit module. Others can be created by multiplying a prefix symbol by a non-prefixed unit:

```
from simtk.unit import mega, kelvin
megakelvin = mega * kelvin
t = 8.3 * megakelvin
```

Only grams and meters get all of the SI prefixes (from yotto-(10^{-24}) to yotta-(10^{24})) automatically.

Converting to different units

Use the `Quantity.in_units_of()` method to create a new Quantity with different units.

```
from simtk.unit import nanosecond, fortnight
x = (175000*nanosecond).in_units_of(fortnight)
```

When you want a plain number out of a Quantity, use the `value_in_unit()` method:

```
from simtk.unit import femtosecond, picosecond
t = 5.0*femtosecond
t_just_a_number = t.value_in_unit(picoseconds)
```

Using `value_in_unit()` puts the responsibility for unit analysis back into your hands, and it should be avoided. It is sometimes necessary, however, when you are called upon to use a non-units-aware Python API.

Lists, tuples, vectors, numpy arrays, and Units

Units can be attached to containers of numbers to create a vector quantity. The simtk.unit module overloads the `__setitem__` and `__getitem__` methods for these containers to ensure that Quantities go in and out.

```
>>> a = Vec3(1,2,3) * nanometers
>>> print(a)
(1, 2, 3) nm
>>> print(a.in_units_of(angstroms))
(10.0, 20.0, 30.0) A
```

```
>>> s2 = [[1,2,3],[4,5,6]] * centimeter
>>> print(s2)
[[1, 2, 3], [4, 5, 6]] cm
>>> print(s2/millimeter)
[[10.0, 20.0, 30.0], [40.0, 50.0, 60.0]]

>>> import numpy
>>> a = numpy.array([1,2,3]) * centimeter
>>> print(a)
[1 2 3] cm
>>> print(a/millimeter)
[ 10.  20.  30.]
```

Converting a whole list to different units at once is much faster than converting each element individually. For example, consider the following code that prints out the position of every particle in a State, as measured in Angstroms:

```
for v in state.getPositions():
    print(v.value_in_unit(angstrom))
```

This can be rewritten as follows:

```
for v in state.getPositions().value_in_unit(angstrom):
    print(v)
```

The two versions produce identical results, but the second one will run faster, and therefore is preferred.

CHAPTER

THIRTEEN

EXAMPLES OF OPENMM INTEGRATION

13.1 GROMACS

GROMACS is a large, complex application written primarily in C. The considerations involved in adapting it to use OpenMM are likely to be similar to those faced by developers of other existing applications.

The first principle we followed in adapting GROMACS was to keep all OpenMM-related code isolated to just a few files, while modifying as little of the existing GROMACS code as possible. This minimized the risk of breaking existing parts of the code, while making the OpenMM-related parts as easy to work with as possible. It also minimized the need for C code to invoke the C++ API. (This would not be an issue if we used the OpenMM C API wrapper, but that is less convenient than the C++ API, and placing all of the OpenMM calls into separate C++ files solves the problem equally well.) Nearly all of the OpenMM-specific code is contained in a single file, openmm_wrapper.cpp. It defines four functions which encapsulate all of the interaction between OpenMM and the rest of GROMACS:

`openmm_init()`: As arguments, this function takes pointers to lots of internal GROMACS data structures that describe the simulation to be run. It creates a System, Integrator, and Context based on them, then returns an opaque reference to an object containing them. That reference is an input argument to all of the other functions defined in openmm_wrapper.cpp. This allows information to be passed between those functions without exposing it to the rest of GROMACS.

`openmm_take_one_step()`: This calls `step(1)` on the Integrator that was created by `openmm_init()`.

`openmm_copy_state()`: This calls `getState()` on the Context that was created by `openmm_init()`, and then copies information from the resulting State into various GROMACS data structures. This function is how state data generated by OpenMM is passed back to GROMACS for output, analysis, etc.

`openmm_cleanup()`: This is called at the end of the simulation. It deletes all the objects that were created by `openmm_init()`.

This set of functions defines the interactions between GROMACS and OpenMM: copying information from the application to OpenMM, performing integration, copying information from OpenMM back to the application, and freeing resources at the end of the simulation. While the details of their implementations are specific to GROMACS, this overall pattern is fairly generic. A similar set of functions can be used for many other applications as well.

13.2 TINKER-OpenMM

TINKER is written primarily in Fortran, and uses common blocks extensively to store application-wide parameters. Rather than modify the TINKER build scripts to allow C++ code, it was decided to use the OpenMM C API instead. Despite these differences, the overall approach used to add OpenMM support was very similar to that used for GROMACS.

TINKER-OpenMM allows OpenMM to be used to calculate forces and energies and to perform the integration in the main molecular dynamics loop. The only changes to the TINKER source code are in the file `dynamic.f` for the setup

and running of a simulation. An added file, `dynamic_openmm.c`, contains the interface C code between TINKER and OpenMM.

The flow of the molecular dynamics simulation using OpenMM is as follows:

1. The TINKER code is used to read the AMOEBA parameter file, the `*.xyz` and `*.key` files. It then parses the command-line options.
2. The routine `map_common_blocks_to_c_data_structs()` is called to map the FORTRAN common blocks to C data structures used in setting the parameters used by OpenMM.
3. The routine `openmm_validate()` is called from `dynamic.f` before the main loop. This routine checks that all required options and settings obtained from the input in step (1) and common blocks in step (2) are available. If an option or setting is unsupported, the program exits with an appropriate message. The routine `openmm_validate()` and the other OpenMM interface methods are in the file `dynamic_openmm.c`.
4. `openmm_init()` is called to create the OpenMM System, Integrator and Context objects..
5. `openmm_take_steps()` is called to take a specified number of time steps.
6. `openmm_update()` is then called to retrieve the state (energies/positions/velocities) and populate the appropriate TINKER data structures. These values are converted from the OpenMM units of kJ/nm to kcal/Å when populating the TINKER arrays.
7. Once the main loop has completed, the routine `openmm_cleanup()` is called to delete the OpenMM objects and release resources being used on the GPU.

CHAPTER

FOURTEEN

TESTING AND VALIDATION OF OPENMM

The goal of testing and validation is to make sure that OpenMM works correctly. That means that it runs without crashing or otherwise failing, and that it produces correct results. Furthermore, it must work correctly on a variety of hardware platforms (e.g. different models of GPU), software platforms (e.g. operating systems and OpenCL implementations), and types of simulations.

Three types of tests are used to validate OpenMM:

- **Unit tests:** These are small tests designed to test specific features or pieces of code in isolation. For example, a test of HarmonicBondForce might create a System with just a few particles and bonds, compute the forces and energy, and compare them to the analytically expected values. There are thousands of unit tests that collectively cover all of OpenMM.
- **System tests:** Whereas unit tests validate small features in isolation, system tests are designed to validate the entire library as a whole. They simulate realistic models of biomolecules and perform tests that are likely to fail if any problem exists anywhere in the library.
- **Direct comparison between OpenMM and other programs:** The third type of validation performed is a direct comparison of the individual forces computed by OpenMM to those computed by other programs for a collection of biomolecules.

Each type of test is outlined in greater detail below; a discussion of the current status of the tests is then given.

14.1 Description of Tests

14.1.1 Unit tests

The unit tests are with the source code, so if you build from source you can run them yourself. See Section 9.7 for details. When you run the tests (for example, by typing "make test" on Linux or Mac), it should produce output something like this:

```
      Start   1: TestReferenceAndersenThermostat
1/317 Test   #1: TestReferenceAndersenThermostat .............. Passed  0.26 sec
      Start   2: TestReferenceBrownianIntegrator
2/317 Test   #2: TestReferenceBrownianIntegrator .............. Passed  0.13 sec
      Start   3: TestReferenceCheckpoints
3/317 Test   #3: TestReferenceCheckpoints ..................... Passed  0.02 sec
... <many other tests> ...
```

Each line represents a test suite, which may contain multiple unit tests. If all tests within a suite passed, it prints the word "Passed" and how long the suite took to execute. Otherwise it prints an error message. If any tests failed, you can then run them individually (each one is a separate executable) to get more details on what went wrong.

14.1.2 System tests

Several different types of system tests are performed. Each type is run for a variety of systems, including both proteins and nucleic acids, and involving both implicit and explicit solvent. The full suite of tests is repeated for both the CUDA and OpenCL platforms, using both single and double precision (and for the integration tests, mixed precision as well), on a variety of operating systems and hardware. There are four types of tests:

- **Consistency between platforms:** The forces and energy are computed using the platform being tested, then compared to ones computed with the Reference platform. The results are required to agree to within a small tolerance.
- **Energy-force consistency:** This verifies that the force really is the gradient of the energy. It first computes the vector of forces for a given conformation. It then generates four other conformations by displacing the particle positions by small amounts along the force direction. It computes the energy of each one, uses those to calculate a fourth order finite difference approximation to the derivative along that direction, and compares it to the actual forces. They are required to agree to within a small tolerance.
- **Energy conservation:** The system is simulated at constant energy using a Verlet integrator, and the total energy is periodically recorded. A linear regression is used to estimate the rate of energy drift. In addition, all constrained distances are monitored during the simulation to make sure they never differ from the expected values by more than the constraint tolerance.
- **Thermostability:** The system is simulated at constant temperature using a Langevin integrator. The mean kinetic energy over the course of the simulation is computed and compared to the expected value based on the temperature. In addition, all constrained distances are monitored during the simulation to make sure they never differ from the expected values by more than the constraint tolerance.

If you want to run the system tests yourself, they can be found in the Subversion repository at https://simtk.org/svn/pyopenmm/trunk/test/system-tests. Check out that directory, then execute the runAllTests.sh shell script. It will create a series of files with detailed information about the results of the tests. Be aware that running the full test suite may take a long time (possibly several days) depending on the speed of your GPU.

14.1.3 Direct comparisons between OpenMM and other programs

As a final check, identical systems are set up in OpenMM and in another program (Gromacs 4.5 or Tinker 6.1), each one is used to compute the forces on atoms, and the results are directly compared to each other.

14.2 Test Results

In this section, we highlight the major results obtained from the tests described above. They are not exhaustive, but should give a reasonable idea of the level of accuracy you can expect from OpenMM.

14.2.1 Comparison to Reference Platform

The differences between forces computed with the Reference platform and those computed with the OpenCL or CUDA platform are shown in Table 14-1. For every atom, the relative difference between platforms was computed as $2\cdot|F_{ref}–F_{test}|/(|F_{ref}|+|F_{test}|)$, where F_{ref} is the force computed by the Reference platform and F_{test} is the force computed by the platform being tested (OpenCL or CUDA). The median over all atoms in a given system was computed to estimate the typical force errors for that system. Finally, the median of those values for all test systems was computed to give the value shown in the table.

Force	OpenCL (single)	OpenCL (double)	CUDA (single)	CUDA (double)
Total Force	$2.53 \cdot 10^{-6}$	$1.44 \cdot 10^{-7}$	$2.56 \cdot 10^{-6}$	$8.78 \cdot 10^{-8}$
HarmonicBondForce	$2.88 \cdot 10^{-6}$	$1.57 \cdot 10^{-13}$	$2.88 \cdot 10^{-6}$	$1.57 \cdot 10^{-13}$
HarmonicAngleForce	$2.25 \cdot 10^{-5}$	$4.21 \cdot 10^{-7}$	$2.27 \cdot 10^{-5}$	$4.21 \cdot 10^{-7}$
PeriodicTorsionForce	$8.23 \cdot 10^{-7}$	$2.44 \cdot 10^{-7}$	$9.27 \cdot 10^{-7}$	$2.56 \cdot 10^{-7}$
RBTorsionForce	$4.86 \cdot 10^{-6}$	$1.46 \cdot 10^{-7}$	$4.72 \cdot 10^{-6}$	$1.4 \cdot 10^{-8}$
NonbondedForce (no cutoff)	$1.49 \cdot 10^{-6}$	$6.49 \cdot 10^{-8}$	$1.49 \cdot 10^{-6}$	$6.49 \cdot 10^{-8}$
NonbondedForce (cutoff, nonperiodic)	$9.74 \cdot 10^{-7}$	$4.88 \cdot 10^{-9}$	$9.73 \cdot 10^{-7}$	$4.88 \cdot 10^{-9}$
NonbondedForce (cutoff, periodic)	$9.82 \cdot 10^{-7}$	$4.88 \cdot 10^{-9}$	$9.8 \cdot 10^{-7}$	$4.88 \cdot 10^{-9}$
NonbondedForce (Ewald)	$1.33 \cdot 10^{-6}$	$5.22 \cdot 10^{-9}$	$1.33 \cdot 10^{-6}$	$5.22 \cdot 10^{-9}$
NonbondedForce (PME)	$3.99 \cdot 10^{-5}$	$4.08 \cdot 10^{-6}$	$3.99 \cdot 10^{-5}$	$4.08 \cdot 10^{-6}$
GBSAOBCForce (no cutoff)	$3.0 \cdot 10^{-6}$	$1.76 \cdot 10^{-7}$	$3.09 \cdot 10^{-6}$	$9.4 \cdot 10^{-8}$
GBSAOBCForce (cutoff, nonperiodic)	$2.77 \cdot 10^{-6}$	$1.76 \cdot 10^{-7}$	$2.95 \cdot 10^{-6}$	$9.33 \cdot 10^{-8}$
GBSAOBCForce (cutoff, periodic)	$2.61 \cdot 10^{-6}$	$1.78 \cdot 10^{-7}$	$2.77 \cdot 10^{-6}$	$9.24 \cdot 10^{-8}$

Table 14-1: Median relative difference in forces between Reference platform and OpenCL/CUDA platform

14.2.2 Energy Conservation

Figure 14-1 shows the total system energy versus time for three simulations of ubiquitin in OBC implicit solvent. All three simulations used the CUDA platform, a Verlet integrator, a time step of 0.5 fs, no constraints, and no cutoff on the nonbonded interactions. They differ only in the level of numeric precision that was used for calculations (see Chapter *11*).

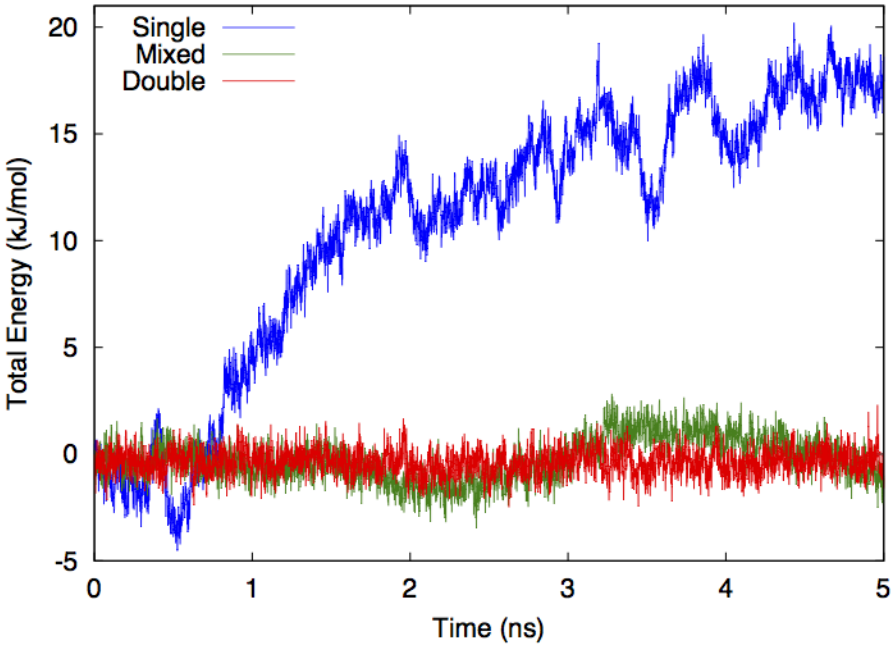

Figure 14-1: Total energy versus time for simulations run in three different precision modes.

For the mixed and double precision simulations, the drift in energy is almost entirely diffusive with negligible systematic drift. The single precision simulation has a more significant upward drift with time, though the rate of

drift is still small compared to the rate of short term fluctuations. Fitting a straight line to each curve gives a long term rate of energy drift of 3.98 kJ/mole/ns for single precision, 0.217 kJ/mole/ns for mixed precision, and 0.00100 kJ/mole/ns for double precision. In the more commonly reported units of kT/ns/dof, these correspond to $4.3 \cdot 10^{-4}$ for single precision, $2.3 \cdot 10^{-5}$ for mixed precision, and $1.1 \cdot 10^{-7}$ for double precision.

Be aware that different simulation parameters will give different results. These simulations were designed to minimize all sources of error except those inherent in OpenMM. There are other sources of error that may be significant in other situations. In particular:

- Using a larger time step increases the integration error (roughly proportional to dt^2).
- If a system involves constraints, the level of error will depend strongly on the constraint tolerance specified by the Integrator.
- When using Ewald summation or Particle Mesh Ewald, the accuracy will depend strongly on the Ewald error tolerance.
- Applying a distance cutoff to implicit solvent calculations will increase the error, and the shorter the cutoff is, the greater the error will be.

As a result, the rate of energy drift may be much greater in some simulations than in the ones shown above.

14.2.3 Comparison to Gromacs

OpenMM and Gromacs 4.5.5 were each used to compute the atomic forces for dihydrofolate reductase (DHFR) in implicit and explicit solvent. The implicit solvent calculations used the OBC solvent model and no cutoff on nonbonded interactions. The explicit solvent calculations used Particle Mesh Ewald and a 1 nm cutoff on direct space interactions. For OpenMM, the Ewald error tolerance was set to 10^{-6}. For Gromacs, `fourierspacing` was set to 0.07 and `ewald_rtol` to 10^{-6}. No constraints were applied to any degrees of freedom. Both programs used single precision. The test was repeated for OpenCL, CUDA, and CPU platforms.

For every atom, the relative difference between OpenMM and Gromacs was computed as $2 \cdot |F_{MM} - F_{Gro}| / (|F_{MM}| + |F_{Gro}|)$, where F_{MM} is the force computed by OpenMM and F_{Gro} is the force computed by Gromacs. The median over all atoms is shown in Table 14-2.

Solvent Model	OpenCL	CUDA	CPU
Implicit	$7.66 \cdot 10^{-6}$	$7.68 \cdot 10^{-6}$	$1.94 \cdot 10^{-5}$
Explicit	$6.77 \cdot 10^{-5}$	$6.78 \cdot 10^{-5}$	$9.89 \cdot 10^{-5}$

Table 14-2: Median relative difference in forces between OpenMM and Gromacs

CHAPTER

FIFTEEN

AMOEBA PLUGIN

OpenMM 7.0 provides a plugin that implements the AMOEBA polarizable atomic multipole force field from Jay Ponder's lab. The AMOEBA force field may be used through OpenMM's Python application layer. We have also created a modified version of TINKER (referred to as TINKER-OpenMM here) that uses OpenMM to accelerate AMOEBA simulations. TINKER-OpenMM can be created from a TINKER package using three files made available through the OpenMM home page. OpenMM AMOEBA Force and System objects containing AMOEBA forces can be serialized.

At present, AMOEBA is only supported on the CUDA and Reference platforms, not on the OpenCL platform.

In the following sections, the individual forces and options available in the plugin are listed, and the steps required to build and use the plugin and TINKER-OpenMM are outlined. Validation results are also reported. Benchmarks can be found on the OpenMM wiki at http://wiki.simtk.org/openmm/Benchmarks.

15.1 OpenMM AMOEBA Supported Forces and Options

15.1.1 Supported Forces and Options

The AMOEBA force terms implemented in OpenMM are listed in Table 15-1 along with the supported and unsupported options. TINKER options that are not supported for any OpenMM force include the grouping of atoms (e.g. protein chains), the infinite polymer check, and no exclusion of particles from energy/force calculations ('active'/'inactive' particles). The virial is not calculated for any force.

All rotation axis types are supported: 'Z-then-X', 'Bisector', 'Z-Bisect', '3-Fold', 'Z-Only'.

TINKER Force	OpenMM Force	Option/Note
ebond1 (bondterm)	AmoebaBondForce	bndtyp='HARMONIC' supported, 'MORSE' not implemented
Eangle71 (angleterm)	AmoebaAngleForce	angtyp='HARMONIC' and 'IN-PLANE' supported; 'LINEAR' and 'FOURIER' not implemented
etors1a (torsionterm)	PeriodicTorsionForce	All options implemented; smoothing version(etors1b) not supported
etortor1 (tortorterm)	AmoebaTorsionTorsionForce	All options implemented
eopbend1 (opbendterm)	AmoebaOutOfPlaneBendForce	opbtyp = 'ALLINGER' implemented; 'W-D-C' not implemented
epitors1 (pitorsterm)	AmoebaPiTorsionForce	All options implemented
estrbnd1 (strbndterm)	AmoebaStretchBendForce	All options implemented
ehal1a (vdwterm)	AmoebaVdwForce	ehal1b(LIGHTS) not supported
empole1a (mpoleterm)	AmoebaMultipoleForce	poltyp = 'MUTUAL', 'DIRECT' supported
empole1c (mpoleterm) PME	AmoebaMultipoleForce	poltyp = 'MUTUAL', 'DIRECT' supported; boundary= 'VACUUM' unsupported
esolv1 (solvateterm)	AmoebaWcaDispersionForce, AmoebaGeneralizedKirkwoodForce	Only born-radius='grycuk' and solvate='GK' supported; unsupported solvate settings: 'ASP', 'SASA', 'ONION', 'pb', 'GB-HPMF', 'Gk-HPMF'; SASA computation is based on ACE approximation
eurey1 (ureyterm)	HarmonicBondForce	All options implemented

Table 15-1: Mapping between TINKER and OpenMM AMOEBA forces

Some specific details to be aware of are the following:

- Forces available in TINKER but not implemented in the OpenMM AMOEBA plugin include the following: angle-angle, out-of-plane distance, improper dihedral, improper torsion, stretch-torsion, charge-charge, atomwise charge-dipole, dipole-dipole, reaction field, ligand field, restraint, scf molecular orbital calculation; strictly speaking, these are not part of the AMOEBA force field.
- Implicit solvent in TINKER-OpenMM is implemented with key file entry 'solvate GK'. The entry 'born-radius grycuk' should also be included; only the 'grycuk' option for calculating the Born radii is available in the plugin.
- In TINKER, the nonpolar cavity contribution to the solvation term is calculated using an algorithm that does not map well to GPUs. Instead the OpenMM plugin uses the TINKER version of the ACE approximation to estimate the cavity contribution to the SASA.
- Calculations using the CUDA platform may be done in either single or double precision; for the Reference platform, double precision is used. TINKER uses double precision.
- The TINKER parameter files for the AMOEBA force-field parameters are based on units of kilocalorie/Å, whereas OpenMM uses units of kilojoules/nanometer; both TINKER and OpenMM use picoseconds time units. Hence, in mapping the force-field parameters from TINKER files to OpenMM, many of the parameter values must be converted to the OpenMM units. The setup methods in the TINKER-OpenMM application perform the required conversions.

15.1.2 Supported Integrators

In addition to the limitations to the forces outlined above, TINKER-OpenMM can only use either the 'Verlet' or 'Stochastic' integrators when the OpenMM plugin is used; an equivalent to the TINKER 'Beeman' integrator is unavailable in OpenMM.

15.2 TINKER-OpenMM

15.2.1 Building TINKER-OpenMM (Linux)

Below are instructions for building TINKER-OpenMM in Linux.

1. To build and install the OpenMM plugin libraries, follow the steps outlined in Chapter 9 (Compiling OpenMM from Source Code). You will need to set the following options to 'ON' when you run CMake:
   (a) OPENMM_BUILD_AMOEBA_PLUGIN
   (b) OPENMM_BUILD_AMOEBA_CUDA_LIB
   (c) OPENMM_BUILD_CUDA_LIB
   (d) OPENMM_BUILD_C_AND_FORTRAN_WRAPPERS
2. Download the complete TINKER distribution from http://dasher.wustl.edu/ffe/ and unzip/untar the file.
3. Obtain the modified TINKER file `dynamic.f`, the interface file `dynamic_openmm.c` and the `Makefile` from the "Downloads" section of OpenMM's homepage (https://simtk.org/home/openmm) and place them in the TINKER source directory. These files are compatible with TINKER 6.0.4. If you are using later versions of TINKER, some minor edits may be required to get the program to compile.
4. In the `Makefile`, edit the following fields, as needed:
   (a) TINKERDIR – This should point to the head of the TINKER distribution directory, e.g., '/home/user/tinker-5.1.09'
   (b) LINKDIR – directory in executable path containing linked copies of the TINKER executables; typical directory would be '/usr/local/bin'
   (c) CC – This is an added field that should point to the C compiler (e.g., '/usr/bin/gcc')
   (d) OpenMM_INSTALL_DIR - This should identify the directory where the OpenMM files were installed, i.e., the OPENMM_INSTALL_PREFIX setting when CMake was run in step (1)
5. At the command line, type:

```
make dynamic_openmm.x
```

   to create the executable.

6. Check that the environment variable 'OPENMM_PLUGIN_DIR' is set to the installed plugins directory and that the environment variable 'LD_LIBRARY_PATH' includes both the installed lib and plugins directory; for example:

```
OPENMM_PLUGIN_DIR=/home/usr/install/openmm/lib/plugins
LD_LIBRARY_PATH=/usr/local/cuda/lib64:/home/usr/install/openmm/lib:
                /home/usr/install/openmm/lib/plugins
```

15.2.2 Using TINKER-OpenMM

Run `dynamic_openmm.x` with the same command-line options as you would `dynamic.x`. Consult the TINKER documentation and Table 15-1 for more details.

Available outputs

Only the total force and potential energy are returned by TINKER-OpenMM; a breakdown of the energy and force into individual terms (bond, angle, . . .), as is done in TINKER, is unavailable through the OpenMM plugin. Also, the pressure cannot be calculated since the virial is not calculated in the plugin.

Setting the frequency of output data updates

Frequent retrieval of the state information from the GPU board can use up a substantial portion of the total wall clock time. This is due to the fact that the forces and energies are recalculated for each retrieval. Hence, if the state information is obtained after every timestep, the wall clock time will approximately double over runs where the state information in only gathered infrequently (say every 50-100 timesteps).

Two options are provided for updating the TINKER data structures:

1. (DEFAULT) If the logical value of 'oneTimeStepPerUpdate' in `dynamic.f` is true, then a single step is taken and the TINKER data structures are populated at each step. This option is conceptually simpler and is consistent with the TINKER md loops; for example, the output from the TINKER subroutine mdstat() will be accurate for this choice. However, the performance will be degraded since the forces and energy are recalculated with each call, doubling the required time. This is the default option.
2. If 'oneTimeStepPerUpdate' is false, then depending on the values of iprint (TINKER keyword 'PRINTOUT') and iwrite (=dump time/dt), multiple time steps are taken on the GPU before data is transferred from the GPU to the CPU; here dump time is the value given to the TINKER command-line query 'Enter Time between Dumps in Picoseconds'. Under this option, every iprint and every iwrite timesteps, the state information will be retrieved. For example if 'PRINTOUT' is 10 and iwrite is 15, then the information will be retrieved at time steps { 10, 15, 20, 30, 40, 45, . . . }. This option will lead to better performance than option 1. However, a downside to this approach is that the fluctuation values printed by the Tinker routine mdstat() will be incorrect.

Specify the GPU board to use

To specify a GPU board other than the default, set the environment variable 'CUDA_DEVICE' to the desired board id. A line like the following will be printed to stderr for the setting CUDA_DEVICE=2:

```
Platform Cuda: setting device id to 2 based on env variable CUDA_DEVICE.
```

Running comparison tests between TINKER and OpenMM routines

To turn on testing (comparison of forces and potential energy for the initial conformation calculated using TINKER routines and OpenMM routines), set 'applyOpenMMTest' to a non-zero value in `dynamic.f`. Note: the program exits after the force/energy comparisons; it does not execute the main molecular dynamics loop.

Testing individual forces: An example key file for testing the harmonic bond term is as follows:

```
parameters /home/user/tinker/params/amoebabio09
verbose
solvate  GK
born-radius  grycuk
polar-eps  0.0001
```

```
integrate  verlet
bondterm only
```

For the other covalent and Van der Waals forces, replace the line `bondterm only` above with the following lines depending on the force to be tested:

```
angle force:              angleterm onl
out-of-plane bend:        opbendterm only
stretch bend force        strbndterm only
pi-torsion force:         pitorsterm only
torsion force:            torsionterm only
torsion-torsion force:    tortorterm only
Urey-Bradley force:       ureyterm only
Van der Waals force:      vdwterm only
```

A sample key file for the multipole force with no cutoffs is given below:

```
parameters /home/user/tinker/params/amoebabio09
verbose
solvate  GK
born-radius  grycuk
polar-eps  0.0001
integrate  verlet
mpoleterm only
polarizeterm
```

A sample key file for PME multipole tests

```
parameters /home/user/tinker/params/amoebabio09
verbose
randomseed  123456789
neighbor-list
vdw-cutoff  12.0
ewald
ewald-cutoff  7.0
pme-grid  64 64 64
polar-eps  0.01
fft-package  fftw
integrate  verlet
mpoleterm only
polarizeterm
```

For the Generalized Kirkwood force, the following entries are needed:

```
parameters /home/user/tinker/params/amoebabio09
verbose
solvate  GK
born-radius  grycuk
polar-eps  0.0001
integrate  verlet
solvateterm only
polarizeterm
mpoleterm
```

For the implicit solvent ('solvate GK' runs) test, the forces and energies will differ due to the different treatments of the cavity term (see Section *15.1.1* above). With these options for the Generalized Kirkwood force, the test routine will remove the cavity contribution from the TINKER and OpenMM forces/energy when performing the comparisons between the two calculations.

To test the multipole force or the Generalized Kirkwood forces with direct polarization, add the following line to the end of the above files:

```
polarization DIRECT
```

Turning off OpenMM / Reverting to TINKER routines

To use the TINKER routines, as opposed to the OpenMM plugin, to run a simulation, set 'useOpenMM' to .false. in `dynamic.f`.

15.3 OpenMM AMOEBA Validation

OpenMM and TINKER 6.1.01 were each used to compute the atomic forces for dihydrofolate reductase (DHFR) in implicit and explicit solvent. Calculations used the CUDA platform, and were repeated for both single and double precision. For every atom, the relative difference between OpenMM and TINKER was computed as $2\cdot|F_{MM}-F_T|/(|F_{MM}|+|F_T|)$, where F_{MM} is the force computed by OpenMM and F_T is the force computed by TINKER. The median over all atoms is shown in Table 15-2.

Because OpenMM and TINKER use different approximations to compute the cavity term, the differences in forces are much larger for implicit solvent than for explicit solvent. We therefore repeated the calculations, removing the cavity term. This yields much closer agreement between OpenMM and TINKER, demonstrating that the difference comes entirely from that one term.

Solvent Model	single	double
Implicit	$1.04\cdot10^{-2}$	$1.04\cdot10^{-2}$
Implicit (no cavity term)	$9.23\cdot10^{-6}$	$1.17\cdot10^{-6}$
Explicit	$3.73\cdot10^{-5}$	$1.83\cdot10^{-7}$

Table 15-2: Median relative difference in forces between OpenMM and TINKER

CHAPTER

SIXTEEN

RING POLYMER MOLECULAR DYNAMICS (RPMD) PLUGIN

Ring Polymer Molecular Dynamics (RPMD) provides an efficient approach to include nuclear quantum effects in molecular simulations.[26] When used to calculate static equilibrium properties, RPMD reduces to path integral molecular dynamics and gives an exact description of the effect of quantum fluctuations for a given potential energy model.[27] For dynamical properties RPMD is no longer exact but has shown to be a good approximation in many cases.

For a system with a classical potential energy $E(q)$, the RPMD Hamiltonian is given by

$$H = \sum_{k=1}^{n} \left(\frac{p_{k^2}}{2m} + E(q_k) + \frac{m(k_B T n)^2}{2\hbar^2} (q_k - q_{k-1})^2 \right)$$

This Hamiltonian resembles that of a system of classical ring polymers where different copies of the system are connected by harmonic springs. Hence each copy of the classical system is commonly referred to as a "bead". The spread of the ring polymer representing each particle is directly related to its De Broglie thermal wavelength (uncertainty in its position).

RPMD calculations must be converged with respect to the number n of beads used. Each bead is evolved at the effective temperature nT, where T is the temperature for which properties are required. The number of beads needed to converge a calculation can be estimated using[28]

$$n > \frac{\hbar \omega_{max}}{k_B T}$$

where ω_{max} is the highest frequency in the problem. For example, for flexible liquid water the highest frequency is the OH stretch at around 3000 cm^{-1}, so around 24 to 32 beads are needed depending on the accuracy required. For rigid water where the highest frequency is only around 1000 cm^{-1}, only 6 beads are typically needed. Due to the replication needed of the classical system, the extra cost of the calculation compared to a classical simulation increases linearly with the number of beads used.

This cost can be reduced by "contracting" the ring polymer to a smaller number of beads.[28] The rapidly changing forces are then computed for the full number of beads, while slower changing forces are computed on a smaller set. In the case of flexible water, for example, a common arrangement would be to compute the high frequency bonded forces on all 32 beads, the direct space nonbonded forces on only 6 beads, and the reciprocal space nonbonded forces on only a single bead.

Due to the stiff spring terms between the beads, NVE RPMD trajectories can suffer from ergodicity problems and hence thermostatting is highly recommended, especially when dynamical properties are not required.[29] The thermostat implemented here is the path integral Langevin equation (PILE) approach.[30] This method couples an optimal white noise Langevin thermostat to the normal modes of each polymer, leaving only one parameter to be chosen by the user which controls the friction applied to the center of mass of each ring polymer. A good choice for this is to use a value similar to that used in a classical calculation of the same system.

CHAPTER

SEVENTEEN

DRUDE PLUGIN

Drude oscillators are a method for incorporating electronic polarizability into a model.[31] For each polarizable particle, a second particle (the "Drude particle") is attached to it by an anisotropic harmonic spring. When both particles are at the same location, they are equivalent to an ordinary point particle. Applying an electric field causes the Drude particle to move a short distance away from its parent particle, creating an induced dipole moment. The polarizability α is related to the charge q on the Drude particle and the spring constant k by

$$\alpha = \frac{q^2}{k}$$

A damped interaction[32] is used between dipoles that are bonded to each other.

The equations of motion can be integrated with two different methods:

1. In the Self Consistent Field (SCF) method, the ordinary particles are first updated as usual. A local energy minimization is then performed to select new positions for the Drude particles. This ensures that the induced dipole moments respond instantly to changes in their environments. This method is accurate but computationally expensive.
2. In the extended Lagrangian method, the positions of the Drude particles are treated as dynamical variables, just like any other particles. A small amount of mass is transferred from the parent particles to the Drude particles, allowing them to be integrated normally. A dual Langevin integrator is used to maintain the center of mass of each Drude particle pair at the system temperature, while using a much lower temperature for their relative internal motion. In practice, this produces dipole moments very close to those from the SCF solution while being much faster to compute.

CHAPTER

EIGHTEEN

THE THEORY BEHIND OPENMM: INTRODUCTION

18.1 Overview

This guide describes the mathematical theory behind OpenMM. For each computational class, it describes what computations the class performs and how it should be used. This serves two purposes. If you are using OpenMM within an application, this guide teaches you how to use it correctly. If you are implementing the OpenMM API for a new Platform, it teaches you how to correctly implement the required kernels.

On the other hand, many details are intentionally left unspecified. Any behavior that is not specified either in this guide or in the API documentation is left up to the Platform, and may be implemented in different ways by different Platforms. For example, an Integrator is required to produce a trajectory that satisfies constraints to within the user-specified tolerance, but the algorithm used to enforce those constraints is left up to the Platform. Similarly, this guide provides the functional form of each Force, but does not specify what level of numerical precision it must be calculated to.

This is an essential feature of the design of OpenMM, because it allows the API to be implemented efficiently on a wide variety of hardware and software platforms, using whatever methods are most appropriate for each platform. On the other hand, it means that a single program may produce meaningfully different results depending on which Platform it uses. For example, different constraint algorithms may have different regions of convergence, and thus a time step that is stable on one platform may be unstable on a different one. It is essential that you validate your simulation methodology on each Platform you intend to use, and do not assume that good results on one Platform will guarantee good results on another Platform when using identical parameters.

18.2 Units

There are several different sets of units widely used in molecular simulations. For example, energies may be measured in kcal/mol or kJ/mol, distances may be in Angstroms or nm, and angles may be in degrees or radians. OpenMM uses the following units everywhere.

Quantity	Units
distance	nm
time	ps
mass	atomic mass units
charge	proton charge
temperature	Kelvin
angle	radians
energy	kJ/mol

These units have the important feature that they form an internally consistent set. For example, a force always has the same units (kJ/mol/nm) whether it is calculated as the gradient of an energy or as the product of a mass and an acceleration. This is not true in some other widely used unit systems, such as those that express energy in kcal/mol.

The header file Units.h contains predefined constants for converting between the OpenMM units and some other common units. For example, if your application expresses distances in Angstroms, you should multiply them by OpenMM::NmPerAngstrom before passing them to OpenMM, and positions calculated by OpenMM should be multiplied by OpenMM::AngstromsPerNm before passing them back to your application.

CHAPTER NINETEEN

STANDARD FORCES

The following classes implement standard force field terms that are widely used in molecular simulations.

19.1 HarmonicBondForce

Each harmonic bond is represented by an energy term of the form

$$E = \frac{1}{2}k(x - x_0)^2$$

where x is the distance between the two particles, x_0 is the equilibrium distance, and k is the force constant. This produces a force of magnitude $k(x\text{-}x_0)$.

Be aware that some force fields define their harmonic bond parameters in a slightly different way: $E = k'(x\text{-}x_0)^2$, leading to a force of magnitude $2k'(x\text{-}x_0)$. Comparing these two forms, you can see that $k = 2k'$. Be sure to check which form a particular force field uses, and if necessary multiply the force constant by 2.

19.2 HarmonicAngleForce

Each harmonic angle is represented by an energy term of the form

$$E = \frac{1}{2}k(\theta - \theta_0)^2$$

where θ is the angle formed by the three particles, θ_0 is the equilibrium angle, and k is the force constant.

As with HarmonicBondForce, be aware that some force fields define their harmonic angle parameters as $E = k'(\theta\text{-}\theta_0)^2$. Be sure to check which form a particular force field uses, and if necessary multiply the force constant by 2.

19.3 PeriodicTorsionForce

Each torsion is represented by an energy term of the form

$$E = k\left(1 + \cos\left(n\theta - \theta_0\right)\right)$$

where θ is the dihedral angle formed by the four particles, θ_0 is the phase offset, n is the periodicity, and k is the force constant.

19.4 RBTorsionForce

Each torsion is represented by an energy term of the form

$$E=\sum_{i=0}^{5}C_i(\cos\phi)^i$$

where ϕ is the dihedral angle formed by the four particles and C_0 through C_5 are constant coefficients.

For reason of convention, PeriodicTorsionForce and RBTorsonForce define the torsion angle differently. θ is zero when the first and last particles are on the *same* side of the bond formed by the middle two particles (the *cis* configuration), whereas ϕ is zero when they are on *opposite* sides (the *trans* configuration). This means that θ = ϕ - π.

19.5 CMAPTorsionForce

Each torsion pair is represented by an energy term of the form

$$E=f\left(\theta_1,\theta_2\right)$$

where θ_1 and θ_2 are the two dihedral angles coupled by the term, and $f(x,y)$ is defined by a user-supplied grid of tabulated values. A natural cubic spline surface is fit through the tabulated values, then evaluated to determine the energy for arbitrary (θ_1, θ_2) pairs.

19.6 NonbondedForce

19.6.1 Lennard-Jones Interaction

The Lennard-Jones interaction between each pair of particles is represented by an energy term of the form

$$E=4\epsilon\left(\left(\frac{\sigma}{r}\right)^{12}-\left(\frac{\sigma}{r}\right)^{6}\right)$$

where r is the distance between the two particles, σ is the distance at which the energy equals zero, and ϵ sets the strength of the interaction. If the NonbondedMethod in use is anything other than NoCutoff and r is greater than the cutoff distance, the energy and force are both set to zero. Because the interaction decreases very quickly with distance, the cutoff usually has little effect on the accuracy of simulations.

Optionally you can use a switching function to make the energy go smoothly to 0 at the cutoff distance. When $r_{switch} < r < r_{cutoff}$, the energy is multiplied by

$$S=1-6x^5+15x^4-10x^3$$

where $x=(r-r_{switch})/(r_{cutoff}-r_{switch})$. This function decreases smoothly from 1 at $r=r_{switch}$ to 0 at $r=r_{cutoff}$, and has continuous first and second derivatives at both ends

When an exception has been added for a pair of particles, σ and ϵ are the parameters specified by the exception. Otherwise they are determined from the parameters of the individual particles using the Lorentz-Berthelot combining rule:

$$\sigma=\frac{\sigma_1+\sigma_2}{2}$$

$$\epsilon=\sqrt{\epsilon_1\epsilon_2}$$

When using periodic boundary conditions, NonbondedForce can optionally add a term (known as a *long range dispersion correction*) to the energy that approximately represents the contribution from all interactions beyond the cutoff distance:[33]

$$E_{\text{cor}} = \frac{8\pi N^2}{V}\left(\frac{\langle\epsilon_{ij}\sigma_{ij}^{12}\rangle}{9{r_c}^9} - \frac{\langle\epsilon_{ij}\sigma_{ij}^{6}\rangle}{3{r_c}^3}\right)$$

where N is the number of particles in the system, V is the volume of the periodic box, r_c is the cutoff distance, σ_{ij} and ϵ_{ij} are the interaction parameters between particle *i* and particle *j*, and $\langle...\rangle$ represents an average over all pairs of particles in the system. When a switching function is in use, there is also a contribution to the correction that depends on the integral of E·(1-S) over the switching interval. The long range dispersion correction is primarily useful when running simulations at constant pressure, since it produces a more accurate variation in system energy with respect to volume.

The Lennard-Jones interaction is often parameterized in two other equivalent ways. One is

$$E = \epsilon\left(\left(\frac{r_{min}}{r}\right)^{12} - 2\left(\frac{r_{min}}{r}\right)^{6}\right)$$

where r_{min} (sometimes known as d_{min}; this is not a radius) is the center-to-center distance at which the energy is minimum. It is related to σ by

$$\sigma = \frac{r_{min}}{2^{1/6}}$$

In turn, r_{min} is related to the van der Waals radius by $r_{min} = 2r_{vdw}$.

Another common form is

$$E = \frac{A}{r^{12}} - \frac{B}{r^6}$$

The coefficients A and B are related to σ and ϵ by

$$\sigma = \left(\frac{A}{B}\right)^{1/6}$$

$$\epsilon = \frac{B^2}{4A}$$

19.6.2 Coulomb Interaction Without Cutoff

The form of the Coulomb interaction between each pair of particles depends on the NonbondedMethod in use. For NoCutoff, it is given by

$$E = \frac{1}{4\pi\epsilon_0}\frac{q_1 q_2}{r}$$

where q_1 and q_2 are the charges of the two particles, and *r* is the distance between them.

19.6.3 Coulomb Interaction With Cutoff

For CutoffNonPeriodic or CutoffPeriodic, it is modified using the reaction field approximation. This is derived by assuming everything beyond the cutoff distance is a solvent with a uniform dielectric constant.[34]

$$E = \frac{q_1 q_2}{4\pi\epsilon_0}\left(\frac{1}{r} + k_{rf}r^2 - c_{rf}\right)$$

$$k_{rf} = \left(\frac{1}{{r_{cutoff}}^3}\right)\left(\frac{\epsilon_{solvent} - 1}{2\epsilon_{solvent} + 1}\right)$$

$$c_{rf} = \left(\frac{1}{r_{cutoff}}\right)\left(\frac{3\epsilon_{solvent}}{2\epsilon_{solvent} + 1}\right)$$

where r_{cutoff} is the cutoff distance and $\epsilon_{solvent}$ is the dielectric constant of the solvent. In the limit $\epsilon_{solvent}$ >> 1, this causes the force to go to zero at the cutoff.

19.6.4 Coulomb Interaction With Ewald Summation

For Ewald, the total Coulomb energy is the sum of three terms: the *direct space sum*, the *reciprocal space sum*, and the *self-energy term*.[35]

$$E = E_{dir} + E_{rec} + E_{self}$$

$$E_{dir} = \frac{1}{2}\sum_{i,j}\sum_{\mathbf{n}} q_i q_j \frac{\text{erfc}\left(\alpha r_{ij,\mathbf{n}}\right)}{r_{ij,\mathbf{n}}}$$

$$E_{rec} = \frac{1}{2\pi V}\sum_{i,j} q_i q_j \sum_{\mathbf{k}\neq 0} \frac{\exp(-(\pi\mathbf{k}/\alpha)^2 + 2\pi i\mathbf{k}\cdot(\mathbf{r}_i - \mathbf{r}_j))}{\mathbf{m}^2}$$

$$E_{self} = -\frac{\alpha}{\sqrt{\pi}}\sum_{i} q_{i^2}$$

In the above expressions, the indices *i* and *j* run over all particles, $\mathbf{n} = (n_1, n_2, n_3)$ runs over all copies of the periodic cell, and $\mathbf{k} = (k_1, k_2, k_3)$ runs over all integer wave vectors from ($-k_{max}$, $-k_{max}$, $-k_{max}$) to (k_{max}, k_{max}, k_{max}) excluding (0, 0, 0). $\mathbf{r}_i$ is the position of particle i , while r_{ij} is the distance between particles *i* and *j*. *V* is the volume of the periodic cell, and α is an internal parameter.

In the direct space sum, all pairs that are further apart than the cutoff distance are ignored. Because the cutoff is required to be less than half the width of the periodic cell, the number of terms in this sum is never greater than the square of the number of particles.

The error made by applying the direct space cutoff depends on the magnitude of $\text{erfc}(\alpha r_{cutoff})$. Similarly, the error made in the reciprocal space sum by ignoring wave numbers beyond k_{max} depends on the magnitude of $\exp(-(\pi k_{max}/\alpha)^2)$. By changing α, one can decrease the error in either term while increasing the error in the other one.

Instead of having the user specify α and $-k_{max}$, NonbondedForce instead asks the user to choose an error tolerance δ. It then calculates α as

$$\alpha = \sqrt{-\log\left(2\delta\right)}/r_{cutoff}$$

Finally, it estimates the error in the reciprocal space sum as

$$error = \frac{k_{max}\sqrt{d\alpha}}{20}\exp(-(\pi k_{max}/d\alpha)^2)$$

where *d* is the width of the periodic box, and selects the smallest value for k_{max} which gives *error* < δ. (If the box is not square, k_{max} will have a different value along each axis.)

This means that the accuracy of the calculation is determined by δ. r_{cutoff} does not affect the accuracy of the result, but does affect the speed of the calculation by changing the relative costs of the direct space and reciprocal space sums.

You therefore should test different cutoffs to find the value that gives best performance; this will in general vary both with the size of the system and with the Platform being used for the calculation. When the optimal cutoff is used for every simulation, the overall cost of evaluating the nonbonded forces scales as $O(N^{3/2})$ in the number of particles.

Be aware that the error tolerance δ is not a rigorous upper bound on the errors. The formulas given above are empirically found to produce average relative errors in the forces that are less than or similar to δ across a variety of systems and parameter values, but no guarantees are made. It is important to validate your own simulations, and identify parameter values that produce acceptable accuracy for each system.

19.6.5 Coulomb Interaction With Particle Mesh Ewald

The Particle Mesh Ewald (PME) algorithm*[36]* is similar to Ewald summation, but instead of calculating the reciprocal space sum directly, it first distributes the particle charges onto nodes of a rectangular mesh using 5th order B-splines. By using a Fast Fourier Transform, the sum can then be computed very quickly, giving performance that scales as O(N log N) in the number of particles (assuming the volume of the periodic box is proportional to the number of particles).

As with Ewald summation, the user specifies the direct space cutoff r_{cutoff} and error tolerance δ. NonbondedForce then selects α as

$$\alpha = \sqrt{-\log(2\delta)}/r_{cutoff}$$

and the number of nodes in the mesh along each dimension as

$$n_{mesh} = \frac{2\alpha d}{3d^{1/5}}$$

where d is the width of the periodic box along that dimension. Alternatively, the user may choose to explicitly set values for these parameters. (Note that some Platforms may choose to use a larger value of n_{mesh} than that given by this equation. For example, some FFT implementations require the mesh size to be a multiple of certain small prime numbers, so a Platform might round it up to the nearest permitted value. It is guaranteed that n_{mesh} will never be smaller than the value given above.)

The comments in the previous section regarding the interpretation of δ for Ewald summation also apply to PME, but even more so. The behavior of the error for PME is more complicated than for simple Ewald summation, and while the above formulas will usually produce an average relative error in the forces less than or similar to δ, this is not a rigorous guarantee. PME is also more sensitive to numerical round-off error than Ewald summation. For Platforms that do calculations in single precision, making δ too small (typically below about $5 \cdot 10^{-5}$) can actually cause the error to increase.

19.7 GBSAOBCForce

19.7.1 Generalized Born Term

GBSAOBCForce consists of two energy terms: a Generalized Born Approximation term to represent the electrostatic interaction between the solute and solvent, and a surface area term to represent the free energy cost of solvating a neutral molecule. The Generalized Born energy is given by*[16]*

$$E = -\frac{1}{2}\left(\frac{1}{\epsilon_{solute}} - \frac{1}{\epsilon_{solvent}}\right)\sum_{i,j}\frac{q_i q_j}{f_{\text{GB}}(d_{ij}, R_i, R_j)}$$

where the indices *i* and *j* run over all particles, ϵ_{solute} and $\epsilon_{solvent}$ are the dielectric constants of the solute and solvent respectively, q_i is the charge of particle *i*, and d_{ij} is the distance between particles *i* and *j*. $f_{\text{GB}}(d_{ij}, R_i, R_j)$ is defined as

$$f_{\text{GB}}(d_{ij}, R_i, R_j) = \left[d_{ij}^2 + R_i R_j \exp\left(\frac{-d_{ij}^2}{4R_i R_j}\right)\right]^{1/2}$$

R_i is the Born radius of particle i, which calculated as

$$R_i = \frac{1}{\rho_i^{-1} - r_i^{-1}\tanh\left(\alpha\Psi_i - \beta\Psi_i^2 + \gamma\Psi_i^3\right)}$$

where α, β, and γ are the GB^{OBC}II parameters α = 1, β = 0.8, and γ = 4.85. ρ_i is the adjusted atomic radius of particle i, which is calculated from the atomic radius r_i as $\rho_i = r_i - 0.009$ nm. Ψ_i is calculated as an integral over the van der Waals spheres of all particles outside particle i:

$$\Psi_i = \frac{\rho_i}{4\pi}\int_{\text{VDW}} \theta\left(|\mathbf{r}| - \rho_i\right)\frac{1}{|\mathbf{r}|^4}d^3\mathbf{r}$$

where $\theta(r)$ is a step function that excludes the interior of particle i from the integral.

19.7.2 Surface Area Term

The surface area term is given by[37][38]

$$E = E_{SA} \cdot 4\pi \sum_i (r_i + r_{solvent})^2 \left(\frac{r_i}{R_i}\right)^6$$

where r_i is the atomic radius of particle i, r_i is its atomic radius, and $r_{solvent}$ is the solvent radius, which is taken to be 0.14 nm. The default value for the energy scale E_{SA} is 2.25936 kJ/mol/nm^2.

19.8 AndersenThermostat

AndersenThermostat couples the system to a heat bath by randomly selecting a subset of particles at the start of each time step, then setting their velocities to new values chosen from a Boltzmann distribution. This represents the effect of random collisions between particles in the system and particles in the heat bath.[39]

The probability that a given particle will experience a collision in a given time step is

$$P = 1 - e^{-f\Delta t}$$

where f is the collision frequency and Δt is the step size. Each component of its velocity is then set to

$$v_i = \sqrt{\frac{k_B T}{m}}R$$

where T is the thermostat temperature, m is the particle mass, and R is a random number chosen from a normal distribution with mean of zero and variance of one.

19.9 MonteCarloBarostat

MonteCarloBarostat models the effect of constant pressure by allowing the size of the periodic box to vary with time.[40][41] At regular intervals, it attempts a Monte Carlo step by scaling the box vectors and the coordinates of each molecule's center by a factor s. The scale factor s is chosen to change the volume of the periodic box from V to V+ΔV:

$$s = \left(\frac{V + \Delta V}{V}\right)^{1/3}$$

The change in volume is chosen randomly as

$$\Delta V = A \cdot r$$

where A is a scale factor and r is a random number uniformly distributed between -1 and 1. The step is accepted or rejected based on the weight function

$$\Delta W = \Delta E + P\Delta V - Nk_B T \ln\left(\frac{V + \Delta V}{V}\right)$$

where ΔE is the change in potential energy resulting from the step, P is the pressure being applied to the system, N is the number of molecules in the system, k_B is Boltzmann's constant, and T is the system temperature. In particular, if $\Delta W \leq 0$ the step is always accepted. If $\Delta W > 0$, the step is accepted with probability $\exp(-\Delta W/k_B T)$.

This algorithm tends to be more efficient than deterministic barostats such as the Berendsen or Parrinello-Rahman algorithms, since it does not require an expensive virial calculation at every time step. Each Monte Carlo step involves two energy evaluations, but this can be done much less often than every time step. It also does not require you to specify the compressibility of the system, which usually is not known in advance.

The scale factor A that determines the size of the steps is chosen automatically to produce an acceptance rate of approximately 50%. It is initially set to 1% of the periodic box volume. The acceptance rate is then monitored, and if it varies too much from 50% then A is modified accordingly.

Each Monte Carlo step modifies particle positions by scaling the centroid of each molecule, then applying the resulting displacement to each particle in the molecule. This ensures that each molecule is translated as a unit, so bond lengths and constrained distances are unaffected.

MonteCarloBarostat assumes the simulation is being run at constant temperature as well as pressure, and the simulation temperature affects the step acceptance probability. It does not itself perform temperature regulation, however. You must use another mechanism along with it to maintain the temperature, such as LangevinIntegrator or AndersenThermostat.

19.10 MonteCarloAnisotropicBarostat

MonteCarloAnisotropicBarostat is very similar to MonteCarloBarostat, but instead of scaling the entire periodic box uniformly, each Monte Carlo step scales only one axis of the box. This allows the box to change shape, and is useful for simulating anisotropic systems whose compressibility is different along different directions. It also allows a different pressure to be specified for each axis.

You can specify that the barostat should only be applied to certain axes of the box, keeping the other axes fixed. This is useful, for example, when doing constant surface area simulations of membranes.

19.11 MonteCarloMembraneBarostat

MonteCarloMembraneBarostat is very similar to MonteCarloBarostat, but it is specialized for simulations of membranes. It assumes the membrane lies in the XY plane. In addition to applying a uniform pressure to regulate the volume of the periodic box, it also applies a uniform surface tension to regulate the cross sectional area of the periodic box in the XY plane. The weight function for deciding whether to accept a step is

$$\Delta W = \Delta E + P\Delta V - S\Delta A - Nk_B T \ln\left(\frac{V + \Delta V}{V}\right)$$

where S is the surface tension and ΔA is the change in cross sectional area. Notice that pressure and surface tension are defined with opposite senses: a larger pressure tends to make the box smaller, but a larger surface tension tends to make the box larger.

MonteCarloMembraneBarostat offers some additional options to customize the behavior of the periodic box:

- The X and Y axes can be either
  - isotropic (they are always scaled by the same amount, so their ratio remains fixed)
  - anisotropic (they can change size independently)
- The Z axis can be either
  - free (its size changes independently of the X and Y axes)
  - fixed (its size does not change)
  - inversely varying with the X and Y axes (so the total box volume does not change)

19.12 CMMotionRemover

CMMotionRemover prevents the system from drifting in space by periodically removing all center of mass motion. At the start of every *n*'th time step (where *n* is set by the user), it calculates the total center of mass velocity of the system:

$$\mathbf{v}_{\text{CM}} = \frac{\sum_i m_i \mathbf{v}_i}{\sum_i m_i}$$

where m_i and $\mathbf{v}_i$ are the mass and velocity of particle *i*. It then subtracts $\mathbf{v}_{\text{CM}}$ from the velocity of every particle.

CHAPTER

TWENTY

CUSTOM FORCES

In addition to the standard forces described in the previous chapter, OpenMM provides a number of "custom" force classes. These classes provide detailed control over the mathematical form of the force by allowing the user to specify one or more arbitrary algebraic expressions. The details of how to write these custom expressions are described in section *20.11*.

20.1 CustomBondForce

CustomBondForce is similar to HarmonicBondForce in that it represents an interaction between certain pairs of particles as a function of the distance between them, but it allows the precise form of the interaction to be specified by the user. That is, the interaction energy of each bond is given by

$$E = f(r)$$

where $f(r)$ is a user defined mathematical expression.

In addition to depending on the inter-particle distance r, the energy may also depend on an arbitrary set of user defined parameters. Parameters may be specified in two ways:

- Global parameters have a single, fixed value.
- Per-bond parameters are defined by specifying a value for each bond.

20.2 CustomAngleForce

CustomAngleForce is similar to HarmonicAngleForce in that it represents an interaction between sets of three particles as a function of the angle between them, but it allows the precise form of the interaction to be specified by the user. That is, the interaction energy of each angle is given by

$$E = f(\theta)$$

where $f(\theta)$ is a user defined mathematical expression.

In addition to depending on the angle θ, the energy may also depend on an arbitrary set of user defined parameters. Parameters may be specified in two ways:

- Global parameters have a single, fixed value.
- Per-angle parameters are defined by specifying a value for each angle.

20.3 CustomTorsionForce

CustomTorsionForce is similar to PeriodicTorsionForce in that it represents an interaction between sets of four particles as a function of the dihedral angle between them, but it allows the precise form of the interaction to be specified by the user. That is, the interaction energy of each angle is given by

$$E = f(\theta)$$

where $f(\theta)$ is a user defined mathematical expression. The angle θ is guaranteed to be in the range [-π, π]. Like PeriodicTorsionForce, it is defined to be zero when the first and last particles are on the same side of the bond formed by the middle two particles (the *cis* configuration).

In addition to depending on the angle θ, the energy may also depend on an arbitrary set of user defined parameters. Parameters may be specified in two ways:

- Global parameters have a single, fixed value.
- Per-torsion parameters are defined by specifying a value for each torsion.

20.4 CustomNonbondedForce

CustomNonbondedForce is similar to NonbondedForce in that it represents a pairwise interaction between all particles in the System, but it allows the precise form of the interaction to be specified by the user. That is, the interaction energy between each pair of particles is given by

$$E = f(r)$$

where $f(r)$ is a user defined mathematical expression.

In addition to depending on the inter-particle distance *r*, the energy may also depend on an arbitrary set of user defined parameters. Parameters may be specified in two ways:

- Global parameters have a single, fixed value.
- Per-particle parameters are defined by specifying a value for each particle.

A CustomNonbondedForce can optionally be restricted to only a subset of particle pairs in the System. This is done by defining "interaction groups". See the API documentation for details.

When using a cutoff, a switching function can optionally be applied to make the energy go smoothly to 0 at the cutoff distance. When $r_{switch} < r < r_{cutoff}$, the energy is multiplied by

$$S = 1 - 6x^5 + 15x^4 - 10x^3$$

where $x = (r - r_{switch})/(r_{cutoff} - r_{switch})$. This function decreases smoothly from 1 at $r = r_{switch}$ to 0 at $r = r_{cutoff}$, and has continuous first and second derivatives at both ends.

When using periodic boundary conditions, CustomNonbondedForce can optionally add a term (known as a *long range truncation correction*) to the energy that approximately represents the contribution from all interactions beyond the cutoff distance:*[33]*

$$E_{cor} = \frac{2\pi N^2}{V} \left\langle \int_{r_{cutoff}}^{\infty} E(r) r^2 dr \right\rangle$$

where *N* is the number of particles in the system, *V* is the volume of the periodic box, and ⟨...⟩ represents an average over all pairs of particles in the system. When a switching function is in use, there is an additional contribution to the

correction given by

$$E'_{cor} = \frac{2\pi N^2}{V} \left\langle \int_{r_{switch}}^{r_{cutoff}} E(r)(1 - S(r))r^2 dr \right\rangle$$

The long range dispersion correction is primarily useful when running simulations at constant pressure, since it produces a more accurate variation in system energy with respect to volume.

20.5 CustomExternalForce

CustomExternalForce represents a force that is applied independently to each particle as a function of its position. That is, the energy of each particle is given by

$$E = f(x, y, z)$$

where $f(x, y, z)$ is a user defined mathematical expression.

In addition to depending on the particle's (x, y, z) coordinates, the energy may also depend on an arbitrary set of user defined parameters. Parameters may be specified in two ways:

- Global parameters have a single, fixed value.
- Per-particle parameters are defined by specifying a value for each particle.

20.6 CustomCompoundBondForce

CustomCompoundBondForce supports a wide variety of bonded interactions. It defines a "bond" as a single energy term that depends on the positions of a fixed set of particles. The number of particles involved in a bond, and how the energy depends on their positions, is configurable. It may depend on the positions of individual particles, the distances between pairs of particles, the angles formed by sets of three particles, and the dihedral angles formed by sets of four particles. That is, the interaction energy of each bond is given by

$$E = f(\{x_i\}, \{r_i\}, \{\theta_i\}, \{\phi_i\})$$

where $f(...)$ is a user defined mathematical expression. It may depend on an arbitrary set of positions $\{x_i\}$, distances $\{r_i\}$, angles $\{\theta_i\}$, and dihedral angles $\{\phi_i\}$.

Each distance, angle, or dihedral is defined by specifying a sequence of particles chosen from among the particles that make up the bond. A distance variable is defined by two particles, and equals the distance between them. An angle variable is defined by three particles, and equals the angle between them. A dihedral variable is defined by four particles, and equals the angle between the first and last particles about the axis formed by the middle two particles. It is equal to zero when the first and last particles are on the same side of the axis.

In addition to depending on positions, distances, angles, and dihedrals, the energy may also depend on an arbitrary set of user defined parameters. Parameters may be specified in two ways:

- Global parameters have a single, fixed value.
- Per-bond parameters are defined by specifying a value for each bond.

20.7 CustomCentroidBondForce

CustomCentroidBondForce is very similar to CustomCompoundBondForce, but instead of creating bonds between individual particles, the bonds are between the centers of groups of particles. This is useful for purposes such as restraining the distance between two molecules or pinning the center of mass of a single molecule.

The first step in computing this force is to calculate the center position of each defined group of particles. This is calculated as a weighted average of the positions of all the particles in the group, with the weights being user defined. The computation then proceeds exactly as with CustomCompoundBondForce, but the energy of each "bond" is now calculated based on the centers of a set of groups, rather than on the positions of individual particles.

This class supports all the same function types and features as CustomCompoundBondForce. In fact, any interaction that could be implemented with CustomCompoundBondForce can also be implemented with this class, simply by defining each group to contain only a single atom.

20.8 CustomManyParticleForce

CustomManyParticleForce is similar to CustomNonbondedForce in that it represents a custom nonbonded interaction between particles, but it allows the interaction to depend on more than two particles. This allows it to represent a wide range of non-pairwise interactions. It is defined by specifying the number of particles N involved in the interaction and how the energy depends on their positions. More specifically, it takes a user specified energy function

$$E = f(\{x_i\}, \{r_i\}, \{\theta_i\}, \{\phi_i\})$$

that may depend on an arbitrary set of positions $\{x_i\}$, distances $\{r_i\}$, angles $\{\theta_i\}$, and dihedral angles $\{\phi_i\}$ from a particular set of N particles.

Each distance, angle, or dihedral is defined by specifying a sequence of particles chosen from among the particles in the set. A distance variable is defined by two particles, and equals the distance between them. An angle variable is defined by three particles, and equals the angle between them. A dihedral variable is defined by four particles, and equals the angle between the first and last particles about the axis formed by the middle two particles. It is equal to zero when the first and last particles are on the same side of the axis.

In addition to depending on positions, distances, angles, and dihedrals, the energy may also depend on an arbitrary set of user defined parameters. Parameters may be specified in two ways:

- Global parameters have a single, fixed value.
- Per-particle parameters are defined by specifying a value for each particle.

The energy function is evaluated one or more times for every unique set of N particles in the system. The exact number of times depends on the *permutation mode*. A set of N particles has $N!$ possible permutations. In `SinglePermutation` mode, the function is evaluated for a single arbitrarily chosen one of those permutations. In `UniqueCentralParticle` mode, the function is evaluated for N of those permutations, once with each particle as the "central particle".

The number of times the energy function is evaluated can be further restricted by specifying *type filters*. Each particle may have a "type" assigned to it, and then each of the N particles involved in an interaction may be restricted to only a specified set of types. This provides a great deal of flexibility in controlling which particles interact with each other.

20.9 CustomGBForce

CustomGBForce implements complex, multiple stage nonbonded interactions between particles. It is designed primarily for implementing Generalized Born implicit solvation models, although it is not strictly limited to that purpose.

The interaction is specified as a series of computations, each defined by an arbitrary algebraic expression. These computations consist of some number of per-particle *computed values*, followed by one or more *energy terms*. A computed value is a scalar value that is computed for each particle in the system. It may depend on an arbitrary set of global and per-particle parameters, and well as on other computed values that have been calculated before it. Once all computed values have been calculated, the energy terms and their derivatives are evaluated to determine the system energy and particle forces. The energy terms may depend on global parameters, per-particle parameters, and per-particle computed values.

Computed values can be calculated in two different ways:

- *Single particle* values are calculated by evaluating a user defined expression for each particle:

  $$value_i = f(...)$$

  where $f(...)$ may depend only on properties of particle *i* (its coordinates and parameters, as well as other computed values that have already been calculated).

- *Particle pair* values are calculated as a sum over pairs of particles:

  $$value_i = \sum_{j \neq i} f(r, ...)$$

  where the sum is over all other particles in the System, and $f(r, ...)$ is a function of the distance *r* between particles *i* and *j*, as well as their parameters and computed values.

Energy terms may similarly be calculated per-particle or per-particle-pair.

- *Single particle* energy terms are calculated by evaluating a user defined expression for each particle:

  $$E = f(...)$$

  where $f(...)$ may depend only on properties of that particle (its coordinates, parameters, and computed values).

- *Particle pair* energy terms are calculated by evaluating a user defined expression once for every pair of particles in the System:

  $$E = \sum_{i,j} f(r, ...)$$

  where the sum is over all particle pairs $i < j$, and $f(r, ...)$ is a function of the distance *r* between particles *i* and *j*, as well as their parameters and computed values.

Note that energy terms are assumed to be symmetric with respect to the two interacting particles, and therefore are evaluated only once per pair. In contrast, expressions for computed values need not be symmetric and therefore are calculated twice for each pair: once when calculating the value for the first particle, and again when calculating the value for the second particle.

Be aware that, although this class is extremely general in the computations it can define, particular Platforms may only support more restricted types of computations. In particular, all currently existing Platforms require that the first computed value *must* be a particle pair computation, and all computed values after the first *must* be single particle computations. This is sufficient for most Generalized Born models, but might not permit some other types of calculations to be implemented.

20.10 CustomHbondForce

CustomHbondForce supports a wide variety of energy functions used to represent hydrogen bonding. It computes interactions between "donor" particle groups and "acceptor" particle groups, where each group may include up to three particles. Typically a donor group consists of a hydrogen atom and the atoms it is bonded to, and an acceptor group consists of a negatively charged atom and the atoms it is bonded to. The interaction energy between each donor group and each acceptor group is given by

$$E = f(\{r_i\}, \{\theta_i\}, \{\phi_i\})$$

where $f(...)$ is a user defined mathematical expression. It may depend on an arbitrary set of distances $\{r_i\}$, angles $\{\theta_i\}$, and dihedral angles $\{\phi_i\}$.

Each distance, angle, or dihedral is defined by specifying a sequence of particles chosen from the interacting donor and acceptor groups (up to six atoms to choose from, since each group may contain up to three atoms). A distance variable is defined by two particles, and equals the distance between them. An angle variable is defined by three particles, and equals the angle between them. A dihedral variable is defined by four particles, and equals the angle between the first and last particles about the axis formed by the middle two particles. It is equal to zero when the first and last particles are on the same side of the axis.

In addition to depending on distances, angles, and dihedrals, the energy may also depend on an arbitrary set of user defined parameters. Parameters may be specified in three ways:

- Global parameters have a single, fixed value.
- Per-donor parameters are defined by specifying a value for each donor group.
- Per-acceptor parameters are defined by specifying a value for each acceptor group.

20.11 Writing Custom Expressions

The custom forces described in this chapter involve user defined algebraic expressions. These expressions are specified as character strings, and may involve a variety of standard operators and mathematical functions.

The following operators are supported: + (add), - (subtract), * (multiply), / (divide), and ^ (power). Parentheses "(" and ")" may be used for grouping.

The following standard functions are supported: sqrt, exp, log, sin, cos, sec, csc, tan, cot, asin, acos, atan, sinh, cosh, tanh, erf, erfc, min, max, abs, floor, ceil, step, delta, select. step(x) = 0 if x < 0, 1 otherwise. delta(x) = 1 if x is 0, 0 otherwise. select(x,y,z) = z if x = 0, y otherwise. Some custom forces allow additional functions to be defined from tabulated values.

Numbers may be given in either decimal or exponential form. All of the following are valid numbers: 5, -3.1, 1e6, and 3.12e-2.

The variables that may appear in expressions are specified in the API documentation for each force class. In addition, an expression may be followed by definitions for intermediate values that appear in the expression. A semicolon ";" is used as a delimiter between value definitions. For example, the expression

```
a^2+a*b+b^2; a=a1+a2; b=b1+b2
```

is exactly equivalent to

```
(a1+a2)^2+(a1+a2)*(b1+b2)+(b1+b2)^2
```

The definition of an intermediate value may itself involve other intermediate values. All uses of a value must appear *before* that value's definition.

CHAPTER

TWENTYONE

INTEGRATORS

21.1 VerletIntegrator

VerletIntegrator implements the leap-frog Verlet integration method. The positions and velocities stored in the context are offset from each other by half a time step. In each step, they are updated as follows:

$$\mathbf{v}_i(t+\Delta t/2) = \mathbf{v}_i(t-\Delta t/2) + \mathbf{f}_i(t)\Delta t/m_i$$

$$\mathbf{r}_i(t+\Delta t) = \mathbf{r}_i(t) + \mathbf{v}_i(t+\Delta t/2)\Delta t$$

where $\mathbf{v}_i$ is the velocity of particle *i*, $\mathbf{r}_i$ is its position, $\mathbf{f}_i$ is the force acting on it, m_i is its mass, and Δt is the time step.

Because the positions are always half a time step later than the velocities, care must be used when calculating the energy of the system. In particular, the potential energy and kinetic energy in a State correspond to different times, and you cannot simply add them to get the total energy of the system. Instead, it is better to retrieve States after two successive time steps, calculate the on-step velocities as

$$\mathbf{v}_i(t) = \frac{\mathbf{v}_i\left(t-\Delta t/2\right) + \mathbf{v}_i\left(t+\Delta t/2\right)}{2}$$

then use those velocities to calculate the kinetic energy at time *t*.

21.2 LangevinIntegrator

LangevinIntegrator simulates a system in contact with a heat bath by integrating the Langevin equation of motion:

$$m_i\frac{d\mathbf{v}_i}{dt} = \mathbf{f}_i - \gamma m_i \mathbf{v}_i + \mathbf{R}_i$$

where $\mathbf{v}_i$ is the velocity of particle *i*, $\mathbf{f}_i$ is the force acting on it, m_i is its mass, γ is the friction coefficient, and $\mathbf{R}_i$ is an uncorrelated random force whose components are chosen from a normal distribution with mean zero and variance $2m_i\gamma k_B T$, where *T* is the temperature of the heat bath.

The integration is done using a leap-frog method similar to VerletIntegrator. *[42]* The same comments about the offset between positions and velocities apply to this integrator as to that one.

21.3 BrownianIntegrator

BrownianIntegrator simulates a system in contact with a heat bath by integrating the Brownian equation of motion:

$$\frac{d\mathbf{r}_i}{dt} = \frac{1}{\gamma m_i}\mathbf{f}_i + \mathbf{R}_i$$

where $\mathbf{r}_i$ is the position of particle i, $\mathbf{f}_i$ is the force acting on it, γ is the friction coefficient, and $\mathbf{R}_i$ is an uncorrelated random force whose components are chosen from a normal distribution with mean zero and variance $2k_BT/m_i\gamma$, where T is the temperature of the heat bath.

The Brownian equation of motion is derived from the Langevin equation of motion in the limit of large γ. In that case, the velocity of a particle is determined entirely by the instantaneous force acting on it, and kinetic energy ceases to have much meaning, since it disappears as soon as the applied force is removed.

21.4 VariableVerletIntegrator

This is very similar to VerletIntegrator, but instead of using the same step size for every time step, it continuously adjusts the step size to keep the integration error below a user-specified tolerance. It compares the positions generated by Verlet integration with those that would be generated by an explicit Euler integrator, and takes the difference between them as an estimate of the integration error:

$$error = (\Delta t)^2 \sum_i \frac{|\mathbf{f}_i|}{m_i}$$

where $\mathbf{f}_i$ is the force acting on particle i and m_i is its mass. (In practice, the error made by the Euler integrator is usually larger than that made by the Verlet integrator, so this tends to overestimate the true error. Even so, it can provide a useful mechanism for step size control.)

It then selects the value of Δt that makes the error exactly equal the specified error tolerance:

$$\Delta t = \sqrt{\frac{\delta}{\sum_i \frac{|\mathbf{f}_i|}{m_i}}}$$

where δ is the error tolerance. This is the largest step that may be taken consistent with the user-specified accuracy requirement.

(Note that the integrator may sometimes choose to use a smaller value for Δt than given above. For example, it might restrict how much the step size can grow from one step to the next, or keep the step size constant rather than increasing it by a very small amount. This behavior is not specified and may vary between Platforms. It is required, however, that Δt never be larger than the value given above.)

A variable time step integrator is generally superior to a fixed time step one in both stability and efficiency. It can take larger steps on average, but will automatically reduce the step size to preserve accuracy and avoid instability when unusually large forces occur. Conversely, when each uses the same step size on average, the variable time step one will usually be more accurate since the time steps are concentrated in the most difficult areas of the trajectory.

Unlike a fixed step size Verlet integrator, variable step size Verlet is not symplectic. This means that for a given average step size, it will not conserve energy as precisely over long time periods, even though each local region of the trajectory is more accurate. For this reason, it is most appropriate when precise energy conservation is not important, such as when simulating a system at constant temperature. For constant energy simulations that must maintain the energy accurately over long time periods, the fixed step size Verlet may be more appropriate.

21.5 VariableLangevinIntegrator

This is similar to LangevinIntegrator, but it continuously adjusts the step size using the same method as VariableVerletIntegrator. It is usually preferred over the fixed step size Langevin integrator for the reasons given above. Furthermore, because Langevin dynamics involves a random force, it can never be symplectic and therefore the fixed step size Verlet integrator's advantages do not apply to the Langevin integrator.

21.6 CustomIntegrator

CustomIntegrator is a very flexible class that can be used to implement a wide range of integration methods. This includes both deterministic and stochastic integrators; Metropolized integrators; multiple time step integrators; and algorithms that must integrate additional quantities along with the particle positions and momenta.

The algorithm is specified as a series of computations that are executed in order to perform a single time step. Each computation computes the value (or values) of a *variable*. There are two types of variables: *global variables* have a single value, while *per-DOF variables* have a separate value for every degree of freedom (that is, every x, y, or z component of a particle). CustomIntegrator defines lots of variables you can compute and/or use in computing other variables. Some examples include the step size (global), the particle positions (per-DOF), and the force acting on each particle (per-DOF). In addition, you can define as many variables as you want for your own use.

The actual computations are defined by mathematical expressions as described in section *20.11*. Several types of computations are supported:

- *Global*: the expression is evaluated once, and the result is stored into a global variable.
- *Per-DOF*: the expression is evaluated once for every degree of freedom, and the results are stored into a per-DOF variable.
- *Sum*: the expression is evaluated once for every degree of freedom. The results for all degrees of freedom are added together, and the sum is stored into a global variable.

There also are other, more specialized types of computations that do not involve mathematical expressions. For example, there are computations that apply distance constraints, modifying the particle positions or velocities accordingly.

CustomIntegrator is a very powerful tool, and this description only gives a vague idea of the scope of its capabilities. For full details and examples, consult the API documentation.

CHAPTER

TWENTYTWO

OTHER FEATURES

22.1 Periodic Boundary Conditions

Many Force objects support periodic boundary conditions. They act as if space were tiled with infinitely repeating copies of the system, then compute the forces acting on a single copy based on the infinite periodic copies. In most (but not all) cases, they apply a cutoff so that each particle only interacts with a single copy of each other particle.

OpenMM supports triclinic periodic boxes. This means the periodicity is defined by three vectors, $\mathbf{a}$, $\mathbf{b}$, and $\mathbf{c}$. Given a particle position, the infinite periodic copies of that particle are generated by adding vectors of the form $i\mathbf{a}+j\mathbf{b}+k\mathbf{c}$, where i, j, and k are arbitrary integers.

The periodic box vectors must be chosen to satisfy certain requirements. Roughly speaking, $\mathbf{a}$, $\mathbf{b}$, and $\mathbf{c}$ need to "mostly" correspond to the x, y, and z axes. They must have the form

$$\mathbf{a} = (a_x, 0, 0)$$
$$\mathbf{b} = (b_x, b_y, 0)$$
$$\mathbf{c} = (c_x, c_y, c_z)$$

It is always possible to put the box vectors into this form by rotating the system until $\mathbf{a}$ is parallel to x and $\mathbf{b}$ lies in the xy plane.

Furthermore, they must obey the following constraints:

$$a_x > 0, b_y > 0, c_z > 0$$
$$a_x \geq 2|b_x|$$
$$a_x \geq 2|c_x|$$
$$b_y \geq 2|c_y|$$

This effectively requires the box vectors to be specified in a particular reduced form. By forming combinations of box vectors (a process known as "lattice reduction"), it is always possible to put them in this form without changing the periodic system they represent.

These requirements have an important consequence: the periodic unit cell can always be treated as an axis-aligned rectangular box of size (a_x, b_y, c_z). The remaining non-zero elements of the box vectors cause the repeating copies of the system to be staggered relative to each other, but they do not affect the shape or size of each copy. The volume of the unit cell is simply given by $a_x b_y c_z$.

22.2 LocalEnergyMinimizer

This provides an implementation of the L-BFGS optimization algorithm. *[43]* Given a Context specifying initial particle positions, it searches for a nearby set of positions that represent a local minimum of the potential energy.

Distance constraints are enforced during minimization by adding a harmonic restraining force to the potential function. The strength of the restraining force is steadily increased until the minimum energy configuration satisfies all constraints to within the tolerance specified by the Context's Integrator.

22.3 XMLSerializer

This provides the ability to "serialize" a System, Force, Integrator, or State object to a portable XML format, then reconstruct it again later. When serializing a System, the XML data contains a complete copy of the entire system definition, including all Forces that have been added to it.

Here are some examples of uses for this class:

1. A model building utility could generate a System in memory, then serialize it to a file on disk. Other programs that perform simulation or analysis could then reconstruct the model by simply loading the XML file.
2. When running simulations on a cluster, all model construction could be done on a single node. The Systems and Integrators could then be encoded as XML, allowing them to be easily transmitted to other nodes.

XMLSerializer is a templatized class that, in principle, can be used to serialize any type of object. At present, however, only System, Force, Integrator, and State are supported.

22.4 Force Groups

It is possible to split the Force objects in a System into groups. Those groups can then be evaluated independently of each other. Some Force classes also provide finer grained control over grouping. For example, NonbondedForce allows direct space computations to be in one group and reciprocal space computations in a different group.

The most important use of force groups is for implementing multiple time step algorithms with CustomIntegrator. For example, you might evaluate the slowly changing nonbonded interactions less frequently than the quickly changing bonded ones. It also is useful if you want the ability to query a subset of the forces acting on the system.

22.5 Virtual Sites

A virtual site is a particle whose position is computed directly from the positions of other particles, not by integrating the equations of motion. An important example is the "extra sites" present in 4 and 5 site water models. These particles are massless, and therefore cannot be integrated. Instead, their positions are computed from the positions of the massive particles in the water molecule.

Virtual sites are specified by creating a VirtualSite object, then telling the System to use it for a particular particle. The VirtualSite defines the rules for computing its position. It is an abstract class with subclasses for specific types of rules. They are:

- TwoParticleAverageSite: The virtual site location is computed as a weighted average of the positions of two particles:

$$\mathbf{r} = w_1\mathbf{r}_1 + w_2\mathbf{r}_2$$

- ThreeParticleAverageSite: The virtual site location is computed as a weighted average of the positions of three particles:

$$\mathbf{r} = w_1\mathbf{r}_1 + w_2\mathbf{r}_2 + w_3\mathbf{r}_3$$

- OutOfPlaneSite: The virtual site location is computed as a weighted average of the positions of three particles and the cross product of their relative displacements:

$$\mathbf{r} = \mathbf{r}_1 + w_{12}\mathbf{r}_{12} + w_{13}\mathbf{r}_{13} + w_{cross}\left(\mathbf{r}_{12} \times \mathbf{r}_{13}\right)$$

  where $\mathbf{r}_{12} = \mathbf{r}_2 - \mathbf{r}_1$ and $\mathbf{r}_{13} = \mathbf{r}_3 - \mathbf{r}_1$. This allows the virtual site to be located outside the plane of the three particles.

- LocalCoordinatesSite: The locations of three other particles are used to compute a local coordinate system, and the virtual site is placed at a fixed location in that coordinate system. The origin of the coordinate system and the directions of its x and y axes are each specified as a weighted sum of the locations of the three particles:

$$\begin{aligned}\mathbf{o} &= w_1^o\mathbf{r}_1 + w_2^o\mathbf{r}_2 + w_3^o\mathbf{r}_3\\ \mathbf{dx} &= w_1^x\mathbf{r}_1 + w_2^x\mathbf{r}_2 + w_3^x\mathbf{r}_3\\ \mathbf{dy} &= w_1^y\mathbf{r}_1 + w_2^y\mathbf{r}_2 + w_3^y\mathbf{r}_3\\ \mathbf{dz} &= \mathbf{dx} \times \mathbf{dy}\end{aligned}$$

  These vectors are then used to construct a set of orthonormal coordinate axes as follows:

$$\begin{aligned}\hat{\mathbf{x}} &= \mathbf{dx}/|\mathbf{dx}|\\ \hat{\mathbf{z}} &= \mathbf{dz}/|\mathbf{dz}|\\ \hat{\mathbf{y}} &= \hat{\mathbf{z}} \times \hat{\mathbf{x}}\end{aligned}$$

  Finally, the position of the virtual site is set to

$$\mathbf{r} = \mathbf{o} + p_1\hat{\mathbf{x}} + p_2\hat{\mathbf{y}} + p_3\hat{\mathbf{z}}$$

22.6 Random Numbers with Stochastic Integrators and Forces

OpenMM includes many stochastic integrators and forces that make extensive use of random numbers. It is impossible to generate truly random numbers on a computer like you would with a dice roll or coin flip in real life—instead programs rely on pseudo-random number generators (PRNGs) that take some sort of initial "seed" value and steps through a sequence of seemingly random numbers.

The exact implementation of the PRNGs is not important (in fact, each platform may have its own PRNG whose performance is optimized for that hardware). What *is* important, however, is that the PRNG must generate a uniform distribution of random numbers between 0 and 1. Random numbers drawn from this distribution can be manipulated to yield random integers in a desired range or even a random number from a different type of probability distribution function (e.g., a normal distribution).

What this means is that the random numbers used by integrators and forces within OpenMM cannot have any discernible pattern to them. Patterns can be induced in PRNGs in two principal ways:

1. The PRNG uses a bad algorithm with a short period.
2. Two PRNGs are started using the same seed

All PRNG algorithms in common use are periodic—that is their sequence of random numbers repeats after a given *period*, defined by the number of "unique" random numbers in the repeating pattern. As long as this period is longer than the total number of random numbers your application requires (preferably by orders of magnitude), the first problem described above is avoided. All PRNGs employed by OpenMM have periods far longer than any current simulation can cycle through.

Point two is far more common in biomolecular simulation, and can result in very strange artifacts that may be difficult to detect. For example, with Langevin dynamics, two simulations that use the same sequence of random numbers appear to synchronize in their global movements.*[44][45]* It is therefore very important that the stochastic forces and integrators in OpenMM generate unique sequences of pseudo-random numbers not only within a single simulation, but between two different simulations of the same system as well (including any restarts of previous simulations).

Every stochastic force and integrator that does (or could) make use of random numbers has two instance methods attached to it: `getRandomNumberSeed()` and `setRandomNumberSeed(int seed)()`. If you set a unique random seed for two different simulations (or different forces/integrators if applicable), OpenMM guarantees that the generated sequences of random numbers will be different (by contrast, no guarantee is made that the same seed will result in identical random number sequences).

Since breaking simulations up into pieces and/or running multiple replicates of a system to obtain more complete statistics is common practice, a new strategy has been employed for OpenMM versions 6.3 and later with the aim of trying to ensure that each simulation will be started with a unique random seed. A random seed value of 0 (the default) will cause a unique random seed to be generated when a new `Context` is instantiated.

Prior to the introduction of this feature, deserializing a serialized `System` XML file would result in each stochastic force or integrator being assigned the same random seed as the original instance that was serialized. If you use a `System` XML file generated by a version of OpenMM older than 6.3 to start a new simulation, you should manually set the random number seed of each stochastic force or integrator to 0 (or another unique value).

BIBLIOGRAPHY

[1] P.A. Kollman, R. Dixon, W. Cornell, T. Fox, C. Chipot, and A. Pohorille. *Computer Simulation of Biomolecular Systems.*, pages 83–96. volume 3. Elsevier, 1997.

[2] J. Wang, P. Cieplak, and P.A. Kollman. How well does a restrained electrostatic potential (RESP) model perform in calculating conformational energies of organic and biological molecules? *Journal of Computational Chemistry*, 21:1049–1074, 2000.

[3] V. Hornak, R. Abel, A. Okur, B. Strockbine, A. Roitberg, and C. Simmerling. Comparison of multiple Amber force fields and development of improved protein backbone parameters. *Proteins*, 65:712–725, 2006.

[4] K. Lindorff-Larsen, S. Piana, K. Palmo, P. Maragakis, J. Klepeis, R.O. Dror, and D.E. Shaw. Improved side-chain torsion potentials for the Amber ff99SB protein force field. *Proteins*, 78:1950–1958, 2010.

[5] D.W. Li and R. Brüschweiler. NMR-based protein potentials. *Angewandte Chemie International Edition*, 49:6778–6780, 2010.

[6] C. Duan, Y.; Wu, S. Chowdhury, M.C. Lee, G. Xiong, W. Zhang, R. Yang, P. Cieplak, R. Luo, and T. Lee. A point-charge force field for molecular mechanics simulations of proteins based on condensed-phase quantum mechanical calculations. *Journal of Computational Chemistry*, 24:1999–2012, 2003.

[7] P. Ren and Jay W. Ponder. A consistent treatment of inter- and intramolecular polarization in molecular mechanics calculations. *Journal of Computational Chemistry*, 23:1497–1506, 2002.

[8] Yue Shi, Zhen Xia, Jiajing Zhang, Robert Best, Chuanjie Wu, Jay W. Ponder, and Pengyu Ren. Polarizable atomic multipole-based AMOEBA force field for proteins. *Journal of Chemical Theory and Computation*, 9(9):4046–4063, 2013.

[9] Pedro E. M. Lopes, Jing Huang, Jihyun Shim, Yun Luo, Hui Li, Benoît Roux, and Alexander D. MacKerell. Polarizable force field for peptides and proteins based on the classical Drude oscillator. *Journal of Chemical Theory and Computation*, 9(12):5430–5449, 2013.

[10] William L. Jorgensen, Jayaraman Chandrasekhar, Jeffry D. Madura, Roger W. Impey, and Michael L. Klein. Comparison of simple potential functions for simulating liquid water. *Journal of Chemical Physics*, 79:926–935, 1983.

[11] Lee-Ping Wang, Todd J. Martinez, and Vijay S. Pande. Building force fields: an automatic, systematic, and reproducible approach. *Journal of Physical Chemistry Letters*, 5:1885–1891, 2014.

[12] Hans W. Horn, William C. Swope, Jed W. Pitera, Jeffry D. Madura, Thomas J. Dick, Greg L. Hura, and Teresa Head-Gordon. Development of an improved four-site water model for biomolecular simulations: TIP4P-Ew. *Journal of Chemical Physics*, 120:9665–9678, 2004.

[13] Michael W. Mahoney and William L. Jorgensen. A five-site model for liquid water and the reproduction of the density anomaly by rigid, nonpolarizable potential functions. *Journal of Chemical Physics*, 112:8910–8922, 2000.

[14] H. J. C. Berendsen, J. R. Grigera, and T. P. Straatsma. The missing term in effective pair potentials. *Journal of Physical Chemistry*, 91:6269–6271, 1987.

[15] Guillaume Lamoureux, Edward Harder, Igor V. Vorobyov, Benoit Roux, and Alexander D. MacKerell Jr. A polarizable model of water for molecular dynamics simulations of biomolecules. *Chemical Physics Letters*, 418(1-3):245–249, 2006.

[16] Alexey Onufriev, Donald Bashford, and David A. Case. Exploring protein native states and large-scale conformational changes with a modified generalized Born model. *Proteins*, 55(22):383–394, 2004.

[17] Michael J. Schnieders and Jay W. Ponder. Polarizable atomic multipole solutes in a generalized Kirkwood continuum. *Journal of Chemical Theory and Computation*, 3:2083–2097, 2007.

[18] Jay W. Ponder. TINKER - Software Tools for Molecular Design, 4.2. 2004.

[19] Gregory D. Hawkins, Christopher J. Cramer, and Donald G. Truhlar. Pairwise solute descreening of solute charges from a dielectric medium. *Chemical Physics Letters*, 246(1-2):122–129, 1995.

[20] John Mongan, Carlos Simmerling, J. Andrew McCammon, David A. Case, and Alexey Onufriev. Generalized Born model with a simple, robust molecular volume correction. *Journal of Chemical Theory and Computation*, 3(1):156–169, 2007.

[21] Hai Nguyen, Daniel R. Roe, and Carlos Simmerling. Improved generalized Born solvent model parameters for protein simulations. *Journal of Chemical Theory and Computation*, 9(4):2020–2034, 2013.

[22] J Srinivasan, M. W. Trevathan, P. Beroza, and D. A. Case. Application of a pairwise generalized Born model to proteins and nucleic acids: inclusion of salt effects. *Theor. Chem. Acc.*, 101:426–434, 1999.

[23] Andrew C. Simmonett, Frank C. Pickard, Yihan Shao, Thomas E. Cheatham, and Bernard R. Brooks. Efficient treatment of induced dipoles. *Journal of Chemical Physics*, 143(7):074115, 2015.

[24] M. Tuckerman, Bruce J. Berne, and Glenn J. Martyna. Reversible multiple time scale molecular dynamics. *Journal of Chemical Physics*, 97(3):1990–2001, 1992.

[25] Donald Hamelberg, Cesar Augusto F. de Oliveira, and J. Andrew McCammon. Sampling of slow diffusive conformational transitions with accelerated molecular dynamics. *Journal of Chemical Physics*, 127(15):155102, 2007.

[26] I. R. Craig and David E. Manolopoulos. Quantum statistics and classical mechanics: real time correlation functions from ring polymer molecular dynamics. *Journal of Chemical Physics*, 121:3368–3373, 2004.

[27] M. Parrinello and A. Rahman. Study of an F center in molten KCl. *Journal of Chemical Physics*, 80(2):860–867, 1984.

[28] Thomas E. Markland and David E. Manolopoulos. An efficient ring polymer contraction scheme for imaginary time path integral simulations. *Journal of Chemical Physics*, 2008.

[29] Randall W. Hall and B. J. Berne. Nonergodicity in path integral molecular dynamics. *Journal of Chemical Physics*, 1984.

[30] M. Ceriotti, M. Parrinello, Thomas E. Markland, and David E. Manolopoulos. Efficient stochastic thermostatting of path integral molecular dynamics. *Journal of Chemical Physics*, 2010.

[31] Guillaume Lamoureux and Benoit Roux. Modeling induced polarization with classical Drude oscillators: theory and molecular dynamics simulation algorithm. *Journal of Chemical Physics*, 119(6):3025–3039, 2003.

[32] B. T. Thole. Molecular polarizabilities calculated with a modified dipole interaction. *Chemical Physics*, 59(3):341–350, 1981.

[33] Michael R. Shirts, David L. Mobley, John D. Chodera, and Vijay S. Pande. Accurate and efficient corrections for missing dispersion interactions in molecular simulations. *Journal of Physical Chemistry B*, 111:13052–13063, 2007.

[34] Ilario G. Tironi, René Sperb, Paul E. Smith, and Wilfred F. van Gunsteren. A generalized reaction field method for molecular dynamics simulations. *Journal of Chemical Physics*, 102(13):5451–5459, 1995.

[35] Abdulnour Y. Toukmaji and John A. Board Jr. Ewald summation techniques in perspective: a survey. *Computer Physics Communications*, 95:73–92, 1996.

[36] Ulrich Essmann, Lalith Perera, Max L. Berkowitz, Tom Darden, Hsing Lee, and Lee G. Pedersen. A smooth particle mesh Ewald method. *Journal of Chemical Physics*, 103(19):8577–8593, 1995.

[37] Michael Schaefer, Christian Bartels, and Martin Karplus. Solution conformations and thermodynamics of structured peptides: molecular dynamics simulation with an implicit solvation model. *Journal of Molecular Biology*, 284(3):835–848, 1998.

[38] Jay W. Ponder. Personal communication.

[39] Hans C. Andersen. Molecular dynamics simulations at constant pressure and/or temperature. *Journal of Chemical Physics*, 72(4):2384–2393, 1980.

[40] Kim-Hung Chow and David M. Ferguson. Isothermal-isobaric molecular dynamics simulations with Monte Carlo volume sampling. *Computer Physics Communications*, 91:283–289, 1995.

[41] Johan Åqvist, Petra Wennerström, Martin Nervall, Sinisa Bjelic, and Bjørn O. Brandsdal. Molecular dynamics simulations of water and biomolecules with a Monte Carlo constant pressure algorithm. *Chemical Physics Letters*, 384:288–294, 2004.

[42] Jesús A. Izaguirre, Chris R. Sweet, and Vijay S. Pande. Multiscale dynamics of macromolecules using Normal Mode Langevin. *Pacific Symposium on Biocomputing*, 15:240–251, 2010.

[43] Dong C. Liu and Jorge Nocedal. On the limited memory BFGS method for large scale optimization. *Mathematical Programming*, 45:503–528, 1989.

[44] Blas P. Uberuaga, Marian Anghel, and Arthur F. Voter. Synchronization of trajectories in canonical molecular-dynamics simulations: observation, explanation, and exploitation. *Journal of Chemical Physics*, 120(14):6363–6374, 2004.

[45] Daniel J. Sindhikara, Seonah Kim, Arthur F. Voter, and Adrian E. Roitberg. Bad Seeds Sprout Perilous Dynamics: Stochastic Thermostat Induced Trajectory Synchronization in Biomolecules. *Journal of Chemical Theory and Computation*, 5(6):1624–1631, April 2009.
